# Three-dimensional bioprinted glioblastoma microenvironments model cellular dependencies and immune interactions

**DOI:** 10.1038/s41422-020-0338-1

**Published:** 2020-06-04

**Authors:** Min Tang, Qi Xie, Ryan C. Gimple, Zheng Zhong, Trevor Tam, Jing Tian, Reilly L. Kidwell, Qiulian Wu, Briana C. Prager, Zhixin Qiu, Aaron Yu, Zhe Zhu, Pinar Mesci, Hui Jing, Jacob Schimelman, Pengrui Wang, Derrick Lee, Michael H. Lorenzini, Deobrat Dixit, Linjie Zhao, Shruti Bhargava, Tyler E. Miller, Xueyi Wan, Jing Tang, Bingjie Sun, Benjamin F. Cravatt, Alysson R. Muotri, Shaochen Chen, Jeremy N. Rich

**Affiliations:** 1 Department of NanoEngineering, University of California San Diego, 9500 Gilman Drive, La Jolla, CA 92093 USA; 2 Division of Regenerative Medicine, Department of Medicine, University of California, San Diego, La Jolla, CA 92037 USA; 3 Sanford Consortium for Regenerative Medicine, 2880 Torrey Pines Scenic Drive, La Jolla, CA 92037 USA; 4 School of Life Sciences, Westlake University, Hangzhou, Zhejiang 310024 China; 5 Key Laboratory of Growth Regulation and Translation Research of Zhejiang Province, School of Life Sciences, Westlake University, Hangzhou, Zhejiang 310024 China; 6 Institute of Basic Medical Sciences, Westlake Institute for Advanced Study, Hangzhou, Zhejiang 310024 China; 7 Department of Pathology, Case Western University, Cleveland, OH USA; 8 Department of Cellular and Molecular Medicine, Cleveland Clinic Lerner Research Institute, Cleveland, OH USA; 9 Department of Cellular & Molecular Medicine, School of Medicine, University of California San Diego, La Jolla, CA 92093 USA; 10 Department of Pediatrics/Rady Children’s Hospital San Diego, School of Medicine, University of California San Diego, La Jolla, CA 92093 USA; 11 The Department of Chemistry and The Skaggs Institute for Chemical Biology, The Scripps Research Institute, La Jolla, CA 92037 USA; 12 Materials Science and Engineering Program, University of California San Diego, 9500 Gilman Drive, La Jolla, CA 92093 USA; 13 Department of Pathology and Center for Cancer Research, Massachusetts General Hospital and Harvard Medical School, Boston, MA 02114 USA; 14 Division of Biological Sciences, University of California San Diego, La Jolla, CA 92093 USA; 15 Kavli Institute for Brain and Mind, University of California San Diego, La Jolla, CA 92093 USA; 16 Center for Academic Research and Training in Anthropogeny (CARTA), La Jolla, CA 92093 USA; 17 Department of Bioengineering, University of California San Diego, 9500 Gilman Drive, La Jolla, CA 92093 USA; 18 Department of Neurosciences, School of Medicine, University of California San Diego, La Jolla, CA 92037 USA

**Keywords:** Cancer stem cells, CNS cancer, Cancer microenvironment, Cancer stem cells

## Abstract

Brain tumors are dynamic complex ecosystems with multiple cell types. To model the brain tumor microenvironment in a reproducible and scalable system, we developed a rapid three-dimensional (3D) bioprinting method to construct clinically relevant biomimetic tissue models. In recurrent glioblastoma, macrophages/microglia prominently contribute to the tumor mass. To parse the function of macrophages in 3D, we compared the growth of glioblastoma stem cells (GSCs) alone or with astrocytes and neural precursor cells in a hyaluronic acid-rich hydrogel, with or without macrophage. Bioprinted constructs integrating macrophage recapitulate patient-derived transcriptional profiles predictive of patient survival, maintenance of stemness, invasion, and drug resistance. Whole-genome CRISPR screening with bioprinted complex systems identified unique molecular dependencies in GSCs, relative to sphere culture. Multicellular bioprinted models serve as a scalable and physiologic platform to interrogate drug sensitivity, cellular crosstalk, invasion, context-specific functional dependencies, as well as immunologic interactions in a species-matched neural environment.

## Introduction

Brain tumors are complex tissues with multicomponent interactions between multiple cell types.^1^ Precision medicine efforts based solely on genomic alterations and molecular circuitries driving neoplastic cells have translated into relatively limited benefit in clinical practice for brain cancers, including glioblastoma, the most prevalent and lethal primary intrinsic brain tumor. Crosstalk between neoplastic cells and the surrounding stroma contributes to tumor initiation, progression, and metastasis. However, most cancer research studies investigate cancer cells in isolation, cultured in non-physiologic adherent conditions containing species-mismatched serum. Massive efforts have interrogated functional dependencies of cancer cell lines.^2–5^ While these studies provide valuable insights into cancer cell dependencies, they lack the capacity to investigate interactions of cancer cells with stromal cells or the microenvironment in an appropriate physiological context. Patient-derived xenografts (PDXs) and genetically engineered mouse models are informative and can better recapitulate the genomic and transcriptomic profiles of patient brain tumors than two-dimensional (2D) culture. However, challenges with engraftment, the low throughput nature of animal experiments, and the lack of normal human cellular interactions, limit their broad applications in clinical settings. In tumors with significant immune cell involvement, such as glioblastoma, PDXs are limited as immunocompromised animals prevent investigation of immune cells in cancer biology.^6^

Methods to construct self-organizing three-dimensional (3D) co-culture systems, termed organoids, have been developed to interrogate physiological and pathophysiological processes.^7,8^ In cancer research, organoid systems serve as models of colorectal cancer,^9,10^ breast cancer,^11,12^ hepatocellular and cholangiocarcinomas,^13^ pancreatic cancers,^14^ and glioblastomas,^15^ among others.^16,17^ In glioblastoma, we first described organoid systems that recapitulate tumor architecture, microenvironmental gradients, and tumor cellular heterogeneity.^15^ Additional glioblastoma models utilize human-embryonic stem cell (hESC)-derived cerebral organoids to investigate interactions between glioblastoma stem cells (GSCs) and normal brain components including infiltration, microenvironmental stimuli, and response to therapies.^18^ However, organoid modeling is labor intensive, relatively low throughput, and highly variable in terms of cellular composition and structure due to the process of self-assembly.

Further development of tissue engineering approaches informs new 3D culture systems with improved scalability and capacity to tune specific biological parameters, including cellular composition and extracellular matrix stiffness.^19^ The development of physiologically relevant brain tumor microenvironments^20^ requires careful consideration of the biophysical and biochemical properties of the matrix and cellular composition of specific tumor types, which can be achieved with recent advances in 3D bioprinting and biomaterials designed specifically for the bioprinting process.^21–24^ Biocompatible scaffolds for tumor microenvironments include the naturally occurring extracellular matrix products chitosan-alginate (CA)^25^ and hyaluronic acid (HA)-based hydrogels,^26,27^ but also synthetic polymers, including poly lactide-co-glycolide (PLGA),^28^ and polyethylene-glycol (PEG),^26^ or polyacrylamide hydrogels.^29^ 3D printing with biocompatible materials is emerging to advance the fields of regenerative medicine and tissue modeling,^21^ with notable relevance and applicability to cancer research.^22^ 3D bioprinting models microenvironmental interactions and drug sensitivities,^18^ reciprocal interactions with macrophages,^23^ and patient-specific screening tools in microfluidics-based systems.^24^ Among many 3D printing technologies, digital light processing (DLP)-based 3D bioprinting provides superior scalability and printing speed in addition to versatility and reproducibility.^30^ Several biomimetic tissue models have been developed using this technology, creating tissue-specific architecture and cellular composition that could be used for functional analyses, metastasis studies, and drug screening.^31,32^

Here, we employ a rapid 3D bioprinting system and photocrosslinkable native ECM derivatives to create a biomimetic 3D cancer microenvironment for the highly lethal brain tumor, glioblastoma. The model is comprised of patient-derived GSCs, macrophages, astrocytes, and neural stem cells (NSCs) in a HA-rich hydrogel. One major microenvironmental feature of glioblastoma is the prominent infiltration of tumor masses by macrophage and microglia. In progressive or recurrent glioblastoma, macrophage and microglia account for a substantial fraction of the tumor bulk. Using genetic depletion, co-implantation, and pharmacologic depletion, macrophage/microglia have been shown to be functionally important for glioblastoma growth, but each of these approaches may have broader effects beyond direct tumor cell-macrophage interactions. Using our rapid 3D bioprinting platform, we can interrogate functional dependencies and multicellular interactions in a physiologically relevant manner.

## Results

### DLP-based rapid 3D bioprinting generates glioblastoma tissue models

**Fig. 1:**
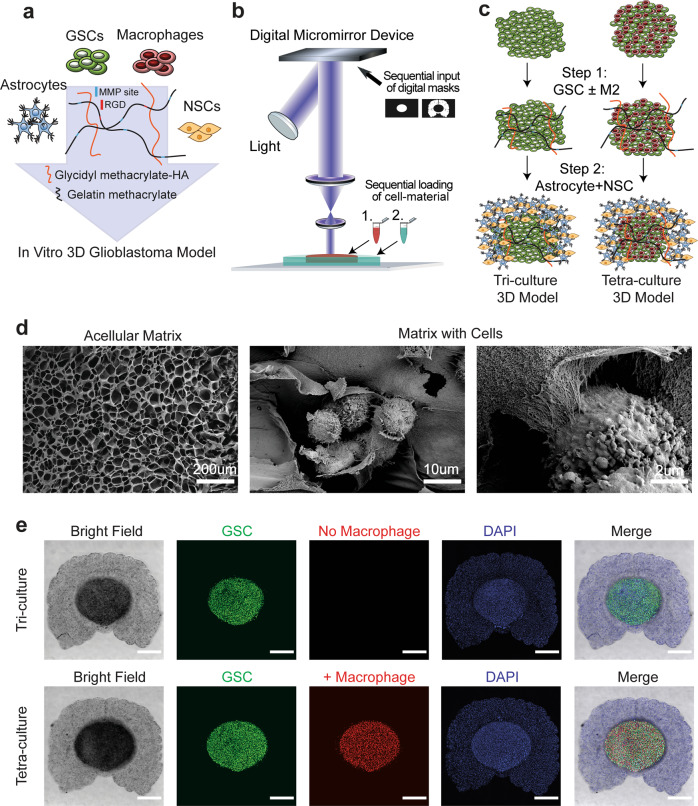
3D bioprinting enables generation of glioblastoma tri-culture and tetra-culture tissue environment model. **a** Schematic diagram of in vitro 3D glioblastoma model containing GSCs, macrophages, astrocytes, and neural stem cells (NSCs). **b** Schematic diagram of digital micromirror device (DMD) chip-based 3D bioprinting system used to produce the 3D glioblastoma model. **c** Diagram of tri-culture (left) and tetra-culture (right) model system. **d** (Left) Scanning electron microscope (SEM) images of acellular glycidyl methylacrylate-hyaluronic acid and gelatin methacrylate extracellular matrix. (Center and Right) SEM images of the cells encapsulated in the extracellular matrix. Scale bars, 200 μm (left), 10 μm (center), and 2 μm (right). **e** Brightfield and immunofluorescence images of the tri-culture and tetra-culture 3D glioblastoma models. GSCs are labeled with green fluorescent protein (GFP) while macrophages are labeled with mCherry. Nuclei are stained with DAPI. Scale bars, 1 mm.

Brain tumors are composed of numerous distinct populations of malignant and supporting stromal cells, and these complex cellular interactions are essential for tumor survival, growth, and progression. Glioblastomas display high levels of intratumoral heterogeneity, with contributions from astrocytes, neurons, NPCs, macrophage/microglia, and vascular components. To move beyond serum-free sphere culture-based models, we utilized a DLP-based rapid 3D bioprinting system to generate 3D tri-culture or tetra-culture glioblastoma tissue models, with a background “normal brain” made up of NPCs and astrocytes and a tumor mass generated by GSCs, with or without macrophage, using brain-specific extracellular matrix (ECM) materials (Fig. 1a). Leveraging this system with exquisite control of cellular constituents in specific locations, we selected macrophage for additional study, as we hypothesized that DLP-based 3D bioprinting could enable precise spatial arrangement of cells and matrix, and selection of any cell type. The key components of the bioprinting system were a digital micromirror device (DMD) chip and a motorized stage where prepolymer cell-material mixtures were sequentially loaded. The DMD chip with approximately 2 × 10^6^ micromirrors controlled the light projection of the brain-shaped patterns onto the printing materials (Fig. 1b). The elliptical pattern corresponded to the core region and the coronal slice pattern corresponded to the peripheral region. Each pattern was printed with 20 s of light exposure. In the 3D tri-culture model, a central tumor core composed of GSCs was surrounded by a less dense population of astrocytes and NPCs. In the 3D tetra-culture model, we mixed M2 macrophages with GSCs within the central core to mimic the immune cell infiltrated tumor mass (Fig. 1c).

The ECM composition of the glioblastoma microenvironment was modeled with gelatin methacrylate (GelMA) and glycidyl methacrylate-HA (GMHA) hydrogels. Cells were encapsulated into a material mixture of 4% GelMA (at 95% degree of methacrylation) and 0.25% GMHA (at 38% degree of methacrylation), which generated a hydrogel matrix that resembled glioblastoma tissue (Supplementary information, Fig. S1a, b). GelMA has good biocompatibility and serves as a stiffness modulator that provided desirable mechanical properties and little intervention in biochemical cues. HA is the most abundant ECM component in healthy brain tissue and promotes glioblastoma progression, including regulating glioblastoma invasion through the receptor for hyaluronan-mediated motility (RHAMM) and CD44, as well as other mechanical and topographical cues.^33^ We used a physiologically relevant concentration of HA (0.25%) determined from clinical analysis of a diverse population of biopsy specimens from patients with different brain tumors.^34^ While a range of molecular weight HAs are present in the brain, low molecular weight HA promotes GSC stemness and resistance.^33^ Thus, in this study, low molecular weight HA (200 kDa) was used to synthesize GMHA to model the pro-invasive brain tumor microenvironment. The mechanical properties of the model were characterized by the compressive modulus and pore sizes. The stiffness of the acellular hydrogel remained stable over a week of incubation at 37 °C (data not shown). The stiffness of cell-encapsulated tumor core was 2.8 ± 0.6 kPa, while the less populated peripheral region containing NPCs and astrocytes was 0.9 ± 0.2 kPa.

The peripheral region stiffness was designed to match that of healthy brain tissue reported to be ~1 kPa. Glioblastoma displays enhanced migration and proliferation in stiffer materials.^33^ The stiffness of the tumor core was modulated with the light exposure time during printing to have higher modulus than the healthy region. The hydrogel had a porosity of 53% and an average pore size of 85 μm. With these microscale features, small molecules, such as drug molecules, freely diffuse through the matrix. Cells closely interacted with other cells and the matrix (Fig. 1d). At a macro scale, the model had a thickness of 1 mm, and 4.4 mm by 3.6 mm in width and length, which allowed gradients of oxygen and nutrition diffusion to be formed within the tissue. Cells were precisely printed into two prearranged regions to provide more physiologically relevant features: a non-neoplastic peripheral region composed of NPCs and astrocytes surrounding a tumor core composed of either GSCs alone or GSCs with macrophage (Fig. 1e). Following optimization for cell density (Supplementary information, Fig. S2a, b), the tumor core in the 3D tri-culture consisted of 2.5 × 10^7^ GSCs/mL, while the tetra-culture tumor core contained 2.5 × 10^7^ GSCs/mL and 1.25 × 10^7^ macrophages/mL.

### 3D bioprinted models recapitulate glioblastoma transcriptional profiles

**Fig. 2:**
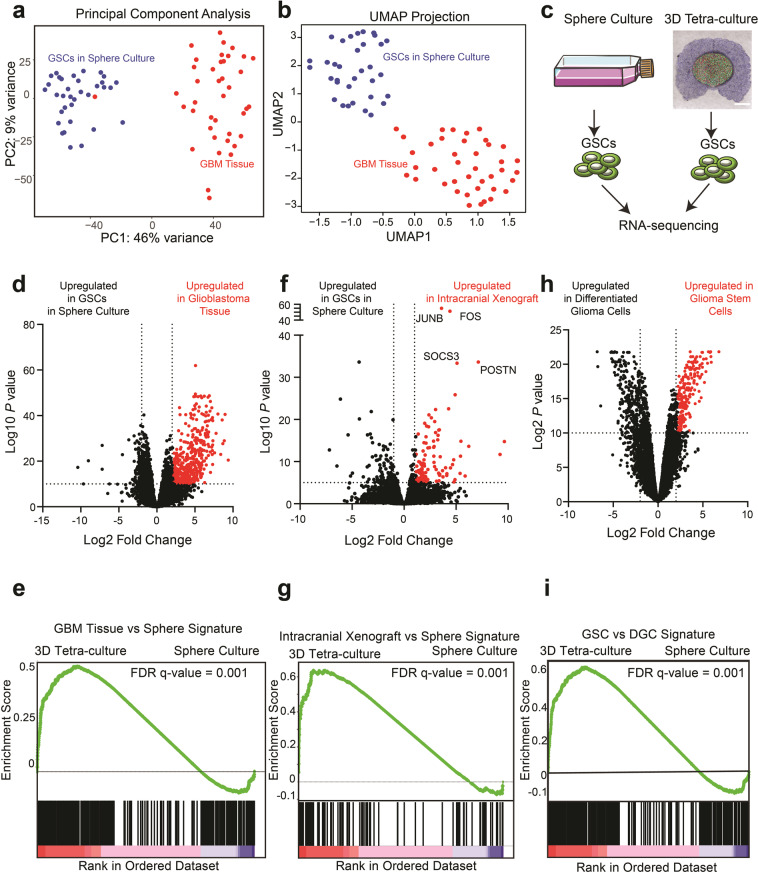
3D tetra-culture models better recapitulate transcriptional signatures found in glioblastoma tissues than standard sphere culture. **a** PCA of the global transcriptional landscape of glioma stem cells in culture (GSCs in culture, *n* = 40) vs primary glioblastoma surgical resection tissues (GBM Tissue, *n* = 34) as defined by RNA-seq. The top 5000 differential genes were used for the analysis. Data was derived from Mack et al.^37^ **b** UMAP of the global transcriptional landscape of glioma stem cells in culture (GSCs in culture, *n* = 40) vs primary glioblastoma surgical resection tissues (GBM Tissue, *n* = 34) as defined by RNA-seq. Analysis parameters include: Sample size of local neighborhood, number of neighbors = 40; learning rate = 0.5; Initialization of low dimensional embedding = random; metrics for computation of distance in high dimensional space = manhattan. Data was derived from Mack et al.^37^ **c** Schematic diagram of experimental approach for GSC RNA-seq experiments. **d** Volcano plot of transcriptional landscape profiled by RNA-seq comparing GSCs in sphere culture (*n* = 40) vs glioblastoma primary surgical resection tissues (*n* = 34). The x-axis depicts the log transformed fold change, while the y-axis shows the log transformed *P* value adjusted for multiple test correction. **e** Gene set enrichment analysis (GSEA) of the glioblastoma tissue vs cell culture signature as defined in **d** when applied to RNA-seq data comparing the 3D tetra-culture system with sphere cell culture. **f** Volcano plot of transcriptional landscape profiled by RNA-seq comparing GSCs in sphere culture (*n* = 2 biological samples with 2 technical replicates each) vs matched orthotopic intracranial xenograft specimens (*n* = 2 biological samples with 2 technical replicates each). The x-axis depicts the log transformed fold change, while the y-axis shows the log transformed *P* value adjusted for multiple test correction. Data was derived from Miller et al.^36^ **g** GSEA of the glioblastoma tissue vs cell culture signature as defined in **f** when applied to RNA-seq data comparing the 3D tetra-culture system with sphere cell culture. **h** Volcano plot of transcriptional landscape profiled by RNA-seq comparing GSCs in sphere culture (*n* = 3 biological samples with 3 technical replicates each) vs differentiated glioma cells (DGCs) in sphere culture (*n* = 3 biological samples with 3 technical replicates each). The x-axis depicts the log transformed fold change, while the y-axis shows the log transformed *P* value adjusted for multiple test correction. Data was derived from Suva et al.^76^ **i** GSEA of the glioblastoma tissue vs cell culture signature as defined in **h** when applied to RNA-seq data comparing the 3D tetra-culture system with sphere cell culture.

Traditionally grown cell lines have been extensively characterized in glioblastoma, revealing that these conditions fail to replicate patient tumors in cellular phenotypes (e.g., invasion) or transcriptional profiles.^35^ While patient-derived glioblastoma cells grown under serum-free conditions enrich for stem-like tumor cells (GSCs) that form spheres and more closely replicate transcriptional profiles and invasive potential than standard culture conditions, we previously demonstrated that spheres display differential transcriptional profiles and cellular dependencies in an RNA interference screen compared to in vivo xenografts.^36^ Based on this background, we interrogated the transcriptional profiles from a large cohort of patient-derived GSCs grown in serum-free, sphere cell culture that we recently reported.^37^ GSCs grown as spheres were transcriptionally distinct from primary glioblastoma surgical resection tissue specimens, when compared through either principal component analysis (PCA) or Uniform Manifold Approximation and Projection (UMAP) (Fig. 2a, b). To determine whether the 3D bioprinted culture systems more closely resemble primary glioblastoma tumors, we performed global transcriptional profiling through RNA extraction followed by next-generation sequencing (RNA-seq) on GSCs isolated from the bioprinted models and on GSCs in sphere culture (Fig. 2c). Upregulation of a core set of glioblastoma tissue-specific genes defined a “Glioblastoma Tissue” gene signature (Fig. 2d). When compared to GSCs grown in sphere culture, the tetra-culture bioprinted model displayed upregulation of the glioblastoma tissue-specific gene set (Fig. 2e), suggesting that the bioprinted model recapitulates transcriptional states present in patient-derived glioblastoma tissues. GSCs in 3D tetra-culture displayed upregulation of genes specifically expressed in orthotopic intracranial xenografts (Fig. 2f, g) and, to a lesser extent, genes specifically expressed in subcutaneous flank xenografts (Supplementary information, Fig. S2c) compared to sphere culture. Additionally, signatures that distinguish GSCs from their differentiated counterparts were upregulated in the tetra-culture system compared to sphere culture (Fig. 2h, i), suggesting that the physiologic tissue environment promotes stem-like transcriptional states.

**Fig. 3:**
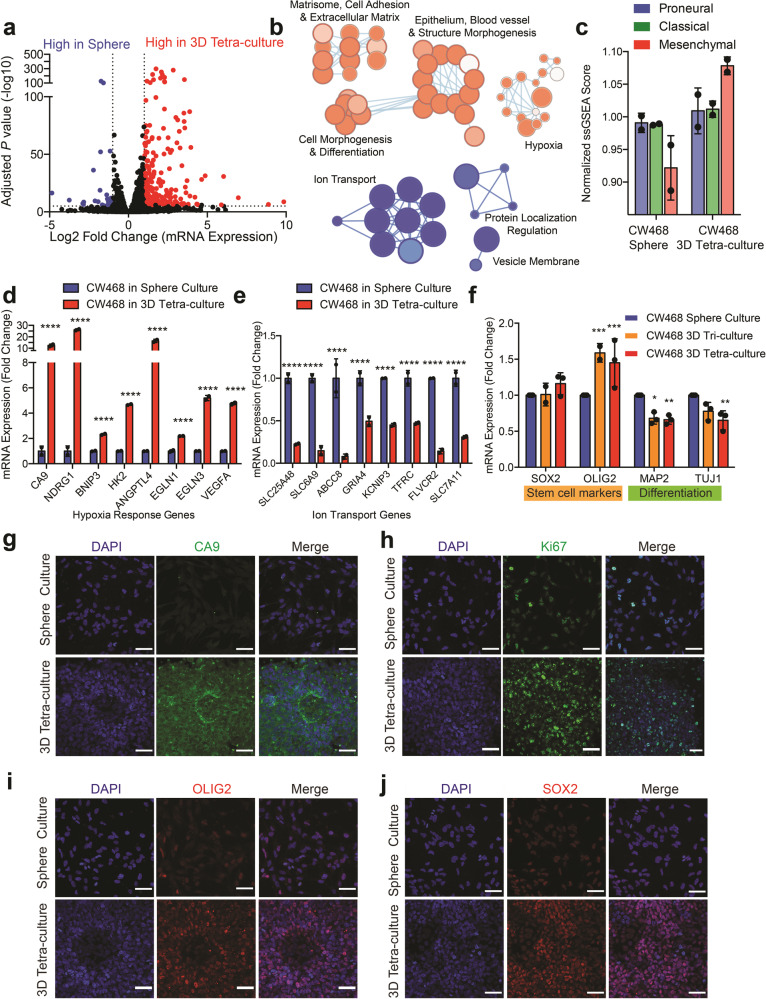
GSCs grown in 3D tetra-culture models upregulate transcriptional signatures of cellular interaction, hypoxia, and cancer stem cells. **a** Volcano plot of transcriptional landscape profiled by RNA-seq comparing the CW468 GSC grown in standard sphere culture vs GSCs in the 3D tetra-culture model. The x-axis depicts the log transformed fold change, while the y-axis shows the log transformed *P* value adjusted for multiple test correction. *n* = 2 technical replicates per condition. **b** Pathway gene set enrichment connectivity diagram displaying pathways enriched among gene sets upregulated (red) and downregulated (blue) in GSCs in the 3D tetra-culture system vs standard sphere culture. **c** Normalized single sample gene set enrichment analysis (ssGSEA) scores of glioblastoma transcriptional subtypes as previously defined^81^ for the CW468 GSC when grown in in standard sphere culture vs GSCs in the 3D tetra-culture model. Bars are centered at the mean value and error bars represent standard deviation. **d** mRNA expression of representative genes in hypoxia response pathways between standard sphere culture vs GSCs in the 3D tetra-culture model as defined by RNA-seq. *P* values were calculated using DESEQ2^75^ with a Wald test with Benjamini and Hochberg correction. ****P < 1e−5. Bars are centered at the mean value and error bars represent standard deviation. **e** mRNA expression of representative genes in ion transport pathways between standard sphere culture vs GSCs in the 3D tetra-culture model as defined by RNA-seq. *P* values were calculated using DESEQ2^75^ with a Wald test with Benjamini and Hochberg correction. ****P < 1e−5. Bars are centered at the mean value and error bars represent standard deviation. **f** mRNA expression of stem cell and differentiation markers between standard sphere culture vs GSCs in the 3D tetra-culture model as defined by quantitative PCR (qPCR). Three technical replicates were used and ordinary two-way ANOVA with Dunnett multiple comparison test was used for statistical analysis, *P < 0.05; **P < 0.01; ***P < 0.001. Bars indicate mean, with error bars showing standard deviation. **g** Immunofluorescence staining of CA9 in cells grown in standard sphere culture (top) vs GSCs in the 3D tetra-culture model (bottom). Scale bars, 50 μm. **h** Immunofluorescence staining of Ki67 in cells grown in standard sphere culture (top) vs GSCs in the 3D tetra-culture model (bottom). Scale bars, 50 μm. **i** Immunofluorescence staining of OLIG2 in cells grown in standard sphere culture (top) vs GSCs in the 3D tetra-culture model (bottom). Scale bars, 50 μm. **j** Immunofluorescence staining of SOX2 in cells grown in standard sphere culture (top) vs GSCs in the 3D tetra-culture model (bottom). Scale bars, 50 μm.

We further interrogated the gene expression profiles that distinguish GSCs grown in sphere culture from the 3D tetra-culture bioprinted models (Fig. 3a). While cells grown in sphere culture displayed enrichment for gene sets involved in ion transport, protein localization, and vesicle membrane function, cells in the tetra-culture 3D model displayed transcriptional upregulation of cell adhesion, extracellular matrix, cell and structure morphogenesis, angiogenesis, and hypoxia signatures (Fig. 3b; Supplementary information, Fig. S3a–c). Furthermore, the tetra-culture model displayed an increase in the mesenchymal glioblastoma signature (Fig. 3c; Supplementary information, Fig. S3b). Hypoxia response genes, CA9, NDRG1, ANGPTL4, and EGLN family members, were upregulated in the tetra-culture system, while various ion transporters, including SLC25A48 and SLC6A9, were downregulated (Fig. 3d, e). By qPCR, GSCs isolated from either 3D system 10 days after printing displayed elevated levels of the stemness marker OLIG2 and decreased levels of the differentiation markers MAP2 and TUJ1 compared to their sphere counterparts grown in parallel (Fig. 3f). Additionally, GSC levels of MAP2 and TUJ1 were decreased to a greater degree in tetra-culture (i.e., with macrophage) compared to tri-culture. We further evaluated the protein expression of stemness, hypoxia, and proliferative markers in the tetra-culture system compared to sphere culture. The hypoxia marker CA9 was upregulated in the tetra-culture model compared to sphere culture (Fig. 3g). The heightened hypoxia level more closely resembled pathologic in vivo conditions, in which the tumor core had a higher hypoxia expression compared to the peripheral region of neurons and astrocytes. In the 3D culture model, cells also showed increased levels of the proliferative marker Ki67 and increased protein expression of the stemness markers OLIG2 and SOX2 (Fig. 3h–j).

### Macrophages promote hypoxic and invasive signatures in bioprinted models

**Fig. 4:**
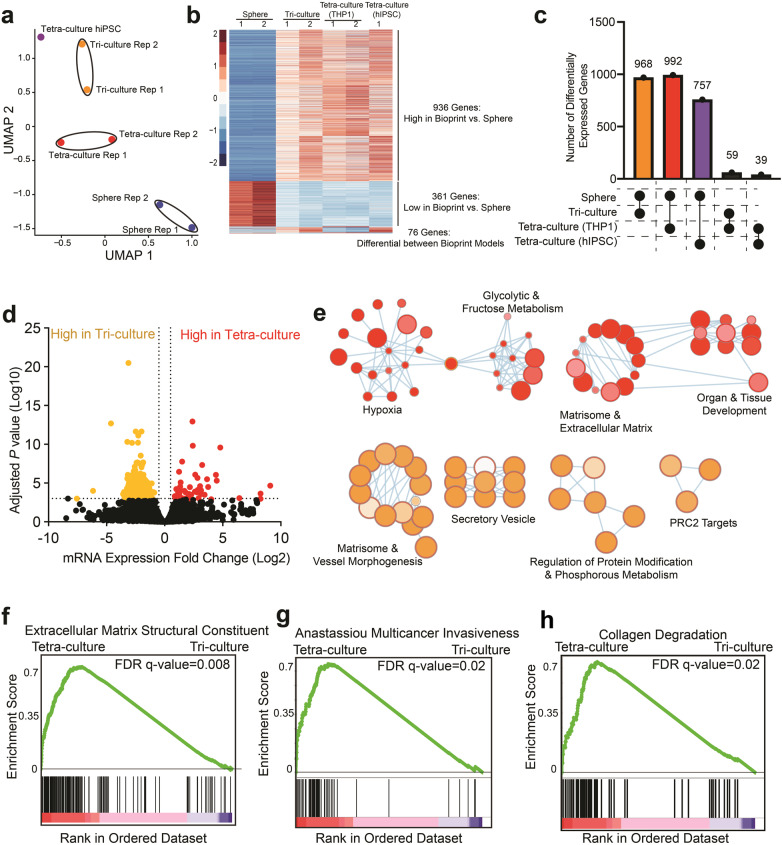
Addition of macrophages activates extracellular matrix and invasiveness signatures. **a** UMAP analysis of RNA-seq data from GSCs grown in (1) sphere culture, (2) tri-culture, (3) tetra-culture with THP1-derived macrophage, and (4) tetra-culture with hiPSC-derived macrophages. **b** Heatmap displaying mRNA expression of differentially expressed genes between conditions. **c** Upset plot showing the number of differentially expressed genes between conditions. For conditions containing sphere cultured cells, genes were considered differentially expressed if the log2 fold change of mRNA expression was greater than 0.5 (or < −0.5) with an adjusted *P* value of 1e−0. For other conditions, genes were considered differentially expressed if the log2 fold change of mRNA expression was greater than 0.5 (or < −0.5) with an adjusted *P* value of 1e−5. **d** Volcano plot of transcriptional landscapes profiled by RNA-seq comparing the CW468 GSC grown in tetra-culture containing THP1-derived macrophages vs GSCs in the tri-culture model. The x-axis depicts the log transformed fold change, while the y-axis shows the log transformed *P* value adjusted for multiple test correction. *n* = 2 technical replicates per condition. **e** Pathway gene set enrichment connectivity diagram displaying pathways enriched among gene sets upregulated (red) and downregulated (orange) in GSCs in the 3D tetra-culture system vs tri-culture system. **f** GSEA of the extracellular matrix structural constituent pathway between tetra-culture and tri-culture models. FDR *q* value = 0.008. **g** GSEA of the Anastassiou multicancer invasiveness pathway between tetra-culture and tri-culture models. FDR *q* value = 0.02. **h** Gene set enrichment analysis (GSEA) of the collagen degradation pathway between tetra-culture and tri-culture models. FDR *q* value = 0.02.

To understand the relative contributions of each cell type incorporated into bioprinted models, we performed RNA-seq on GSCs derived from tri-cultures and tetra-cultures. Given that THP1-derived macrophages display distinct expression profiles as primary macrophages, we built tetra-cultures containing THP1-derived macrophage, human induced pluripotent stem cell (hiPSC)-derived macrophage generated from an established protocol,^38^ and primary human volunteer-derived macrophage. Both hiPSC-derived macrophage and primary macrophage integrated into the tetra-culture models. UMAP clustering revealed that the transcriptional outputs of sphere cultured GSCs are distinct from that of GSCs in bioprinted models (Fig. 4a, b). Concordantly, we detected differentially expressed genes between sphere cultured cells and any of the bioprinted models (757–968 differentially expressed genes), while there were fewer genes that distinguished the bioprinted models (39–59 differentially expressed genes) (Fig. 4c). Bioprinted models were characterized by activation of invasion, extracellular matrix, cell surface interaction, and hypoxia signatures, while GSCs in sphere culture expressed cell cycle, DNA replication, RNA processing, and mitochondrial translation signatures (Supplementary information, Fig. S4). Multiple genes in the hypoxia, biological adhesion and extracellular matrix, and the mesenchymal glioblastoma subtype signature were consistently upregulated across bioprinted models (Supplementary information, Fig. S5a–d). When grown in bioprinted models, GSCs transitioned from an initial proneural/classical transcriptional subtype to a mesenchymal state (Supplementary information, Fig. S5e). These findings were validated by qPCR (Supplementary information, Fig. S5f).

We next investigated differentially expressed pathways between bioprinted models to interrogate the contributions of cellular components. Tri-culture-derived GSCs upregulated extracellular matrix and biological adhesion pathways compared to GSCs in sphere culture (Supplementary information, Fig. S6a–e). Addition of macrophage further increased activation of hypoxia and glycolytic metabolism signatures, with enrichment for invasiveness signatures (Fig. 4d–h). Tetra-cultures constructed with hiPSC-derived macrophage expressed higher levels of extracellular matrix and wound healing and platelet activation signatures and decreased levels of neuron and glial development and differentiation pathways compared to tetra-cultures containing THP1-derived macrophages (Supplementary information, Fig. S7a, b). Incorporation of primary human macrophages did not affect levels of Ki67 or SOX2 compared to use of THP1-derived cells (Supplementary information, Fig. S7c, d). Consistent with our previous findings, use of hiPSC-derived macrophages reduced GSC expression of MAP2 and TUJ1 differentiation markers and increased expression of CA9 and NDRG1 hypoxia markers (Supplementary information, Fig. S7e). Taken together, GSCs upregulate extracellular matrix interaction signatures in response to growth in a bioprinted model. The addition of macrophage further accentuates these gene activation signatures and increases activation of hypoxia and pro-invasive transcriptional profiles.

### 3D bioprinted tissues model complex cellular interactions and migration

**Fig. 5:**
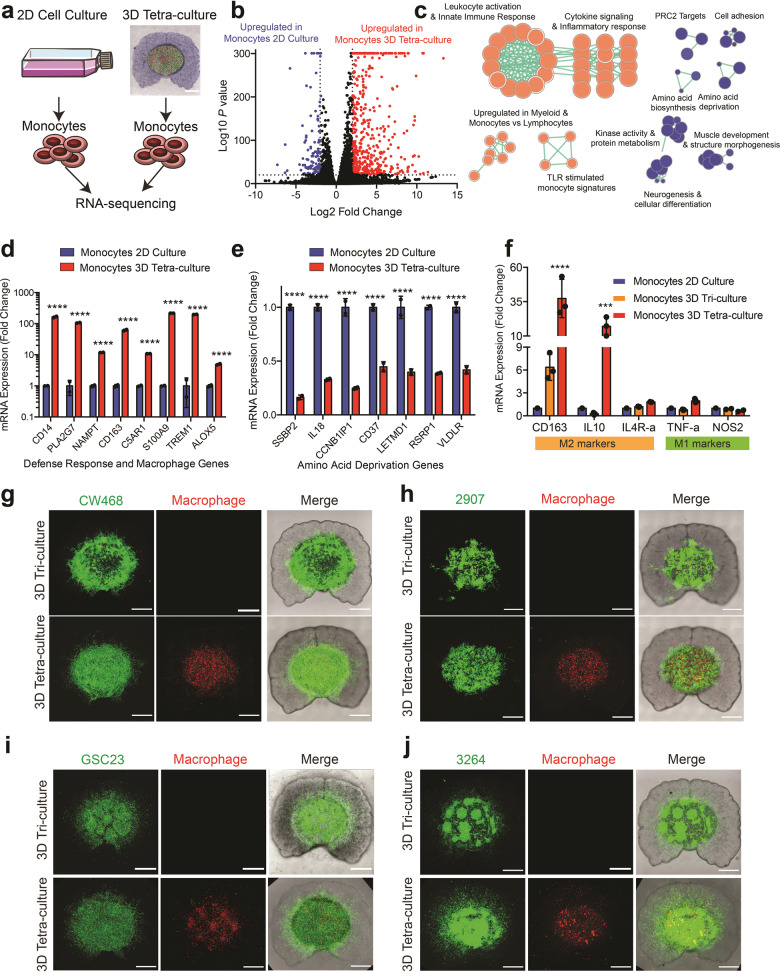
Macrophages grown in 3D tetra-culture models upregulate immune activation signatures, increase M2 polarization, and promote GSC invasion. **a** Schematic diagram of experimental approach for macrophage RNA-seq experiments. **b** Volcano plot of transcriptional landscape profiled by RNA-seq comparing macrophages grown in standard sphere culture vs macrophages in the 3D tetra-culture model. The x-axis depicts the log transformed fold change, while the y-axis shows the log transformed *P* value adjusted for multiple test correction. **c** Pathway gene set enrichment connectivity diagram displaying pathways enriched among gene sets upregulated (red) and downregulated (blue) in macrophages in the 3D tetra-culture system vs standard sphere culture. **d** mRNA expression of representative genes in defense response and macrophage function pathways between standard sphere culture vs macrophages in the 3D tetra-culture model as defined by RNA-seq. *P* values were calculated using DESEQ2^75^ with a Wald test with Benjamini and Hochberg correction. ****P < 1e−20. Bars are centered at the mean value and error bars represent standard deviation. **e** mRNA expression of representative genes in amino acid deprivation pathways between standard sphere culture vs macrophages in the 3D tetra-culture model as defined by RNA-seq. *P* values were calculated using DESEQ2^75^ with a Wald test with Benjamini and Hochberg correction. ****P < 1e−20. Bars are centered at the mean value and error bars represent standard deviation. **f** mRNA expression of M1 and M2 macrophage polarization markers between standard sphere culture vs macrophages in the 3D tetra-culture model as defined by qPCR. Three technical replicates were used and ordinary two-way ANOVA with Dunnett multiple comparison test was used for statistical analysis, ***P < 0.001; ****P < 0.0001. Bars indicate mean, with error bars showing standard deviation. **g** Fluorescence imaging of CW468 GSCs (green) and macrophages (red) grown in the 3D tri-culture model without macrophages (top) vs the 3D tetra-culture model with macrophages (bottom). Scale bars, 1 mm. **h** Fluorescence imaging of 2907 GSCs (green) and macrophages (red) grown in the 3D tri-culture model without macrophages (top) vs the 3D tetra-culture model with macrophages (bottom). Scale bars, 1 mm. **i** Fluorescence imaging of GSC23 GSCs (green) and macrophages (red) grown in the 3D tri-culture model without macrophages (top) vs the 3D tetra-culture model with macrophages (bottom). Scale bars, 1 mm. **j** Fluorescence imaging of 3264 GSCs (green) and macrophages (red) grown in the 3D tri-culture model without macrophages (top) vs the 3D tetra-culture model with macrophages (bottom). Scale bars, 1 mm.

Interactions between malignant cells and stromal components shape tumor tissue with each cell type impacting the other tissue components. To understand these changes, we investigated how macrophage responded to the 3D brain tumor microenvironment by isolating THP1-derived macrophages from 3D bioprinted constructs and performing RNA-seq (Fig. 5a, b). For the 3D printed tissue, macrophage were mixed with GSCs at a 1:2 ratio to form the tumor core, while the periphery was formed by astrocytes and NPCs using the same composition described previously. The transcriptional output of macrophage grown in traditional culture displayed enrichment for PRC2 complex targets, amino acid biosynthesis, protein metabolism signatures and ribosomal pathways, while macrophage exposed to GSCs in the bioprinted construct showed elevation of pathways involved in leukocyte activation and innate immune response, cytokine signaling and inflammatory responses, and TLR-stimulated signatures (Fig. 5c; Supplementary information, Fig. S8a–d). Defense response genes, including CH14, PLA2G7, and ALOX5, were upregulated in macrophage derived from the tetra-culture system, while genes involved in amino acid restriction, including IL18, CD37, and VLDLR, were downregulated (Fig. 5d, e). M2 macrophage-related markers were upregulated in the 3D tetra-cultures, with CD163 increased by 37-fold and IL-10 increased by 17-fold compared to traditional suspension culture, as measured by qPCR. M1-related markers, including TNF-α and NOS2, did not increase, demonstrating that the 3D printed microenvironment preferentially polarized macrophage towards the M2 phenotype (Fig. 5f). This is consistent with the M2 polarization of macrophage in glioblastoma tumors.^39,40^ Gene expression signatures defining peripherally-derived tumor-associated macrophage in glioma ^41,42^ were selectively enriched in macrophage derived from tetra-culture models compared to those grown in 2D culture (Supplementary information, Fig. S9). Collectively, macrophage grown in our 3D bioprinted tetra-culture model expressed gene expression signatures consistent with patient-derived tumor-associated macrophage.

We interrogated the functional consequences of the addition of immune components to the 3D bioprinted model. In four patient-derived GSCs spanning three major glioblastoma transcriptional subtypes (proneural, classical, and mesenchymal), the addition of THP1-derived M2 macrophage increased GSC invasion into the surrounding brain-like parenchyma (Fig. 5g–j). Consistent with our gene expression analyses, M2 macrophage increased the area of invasion by 20% for CW468, 60% for GSC23, 41% for GSC3264, and 30% for GSC2907. Collectively, these results support the tetra-culture model as an effective tool to study cancer cell invasion and the mechanisms by which cellular interactions impinge upon these processes.

As numerous stromal compartments, including neural progenitor cells, astrocytes, and neurons,^43–45^ interact with glioblastoma cells within patient tumors, we interrogated the effects of the bioprinted model on neuronal and oligodendrocyte differentiation of the non-neoplastic NPCs. In 2D culture, most NPCs expressed the proliferative NPC marker SOX2. The high expression and frequency of SOX2 was retained in tri-cultures and tetra-cultures containing macrophage derived from THP1 cells or primary human macrophage (Supplementary information, Fig. S10a). In 2D culture, NPCs expressed the neuronal marker TUBB3, but retained a progenitor-like cellular morphology. In bioprinted models, NPCs adopted a neuronal morphology with the appearance of elongated cellular projections (Supplementary information, Fig. S10b). Expression of MAP2 was reduced in NPCs in bioprinted models compared to 2D culture (Supplementary information, Fig. S11a). OLIG2 staining revealed oligodendrocyte-like cells in tri-cultures (Supplementary information, Fig. S11b). Taken together, NPCs partially differentiate in our bioprinted system, but are unlikely to form mature functional neurons or oligodendrocytes.

### The 3D bioprinted model serves as a platform for drug response modeling

**Fig. 6:**
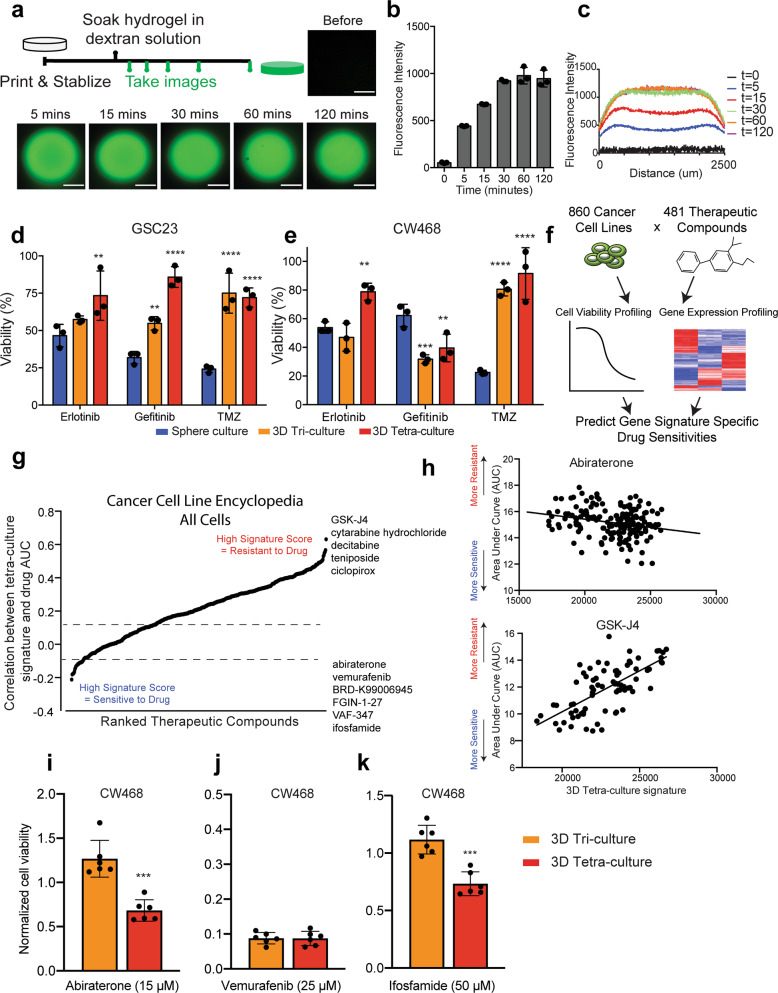
3D bioprinting enables a drug discovery platform and microenvironmental interactions contribute to drug resistance. **a** (Top) Schematic diagram of drug diffusion experiment. (Bottom) Images of FITC-dextran diffusion through the 3D hydrogel over a time course. Scale bars, 1 mm. **b** Average intensity of FITC-dextran signal through the 3D tetra-culture model over a time course. Three replicates were used. Bars indicate mean with error bars showing standard deviation. Ordinary one-way ANOVA with Tukey correction for multiple comparisons was used for statistical analysis. **c** Spatial intensity of FITC-dextran signal through the 3D tetra-culture model over a time course. **d** Cell viability of the GSC23 GSC following treatment with the EGFR inhibitors, erlotinib and gefitinib, and the alkylating agent temozolomide (TMZ) in standard sphere culture conditions, the 3D tri-culture model, and the 3D tetra-culture model. Three replicates were used, ordinary two-way ANOVA with Dunnett multiple test correction was used for statistical analysis. Bars indicate mean, while error bars show standard deviation. **P < 0.01; ****P < 0.0001. **e** Cell viability of the CW468 GSC following treatment with the EGFR inhibitors, erlotinib and gefitinib, and the alkylating agent TMZ in standard sphere culture conditions, the 3D tri-culture model, and the 3D tetra-culture model. Three replicates were used, ordinary two-way ANOVA with Dunnett multiple test correction was used for statistical analysis. Bars indicate mean, while error bars show standard deviation. **P < 0.01; ***P < 0.001; ****P < 0.0001. **f** Schematic diagram of process to determine drug sensitivity based on the 3D tetra-culture gene expression signature from the CCLE and CTRP datasets.^49–51^ **g** Therapeutic efficacy prediction of drugs in all cancer cells in the CTRP dataset based on differentially expressed genes between the 3D tetra-culture model and GSCs grown in sphere culture as defined by RNA-seq. **h** Correlation of (top) abiraterone and (bottom) GSK-J4 sensitivities based on the 3D tetra-culture signature expression across all cancer cell lines in the CCLE dataset. Compounds are ranked based on the correlation between the tetra-culture gene expression signature and compound area under the curve (AUC). **i** Normalized cell viability of GSCs in tri-culture and tetra-culture models following treatment with 15 μM of abiraterone. ***P < 0.001. Bar shows mean of six technical replicates and error bars indicate standard deviation. Unpaired two-tailed *t-*test was used for statistical analysis. **j** Normalized cell viability of GSCs in tri-culture and tetra-culture models following treatment with 25 μM of vemurafenib. ns, *P* > 0.05. Bar shows mean of six technical replicates and error bars indicate standard deviation. Unpaired two-tailed *t-*test was used for statistical analysis. **k** Normalized cell viability of GSCs in tri-culture and tetra-culture models following treatment with 50 μM of ifosfamide. ***P < 0.001. Bar shows mean of six technical replicates and error bars indicate standard deviation. Unpaired two-tailed *t-*test was used for statistical analysis.

We next investigated the ability of our 3D bioprinted constructs to model drug responses and the capacity for cellular interactions within the 3D bioprinted constructs to affect drug sensitivity of GSCs. Fluorescent dextran molecules (4 kDa) modeled drug penetration into 3D bioprinted models.^31,46^ Dextran molecules rapidly entered bioprinted constructs when the hydrogel was soaked in a dextran solution, with rapid increases in average fluorescence intensity measured from the hydrogel. The fluorescence intensity plateaued after 30 min of incubation and displayed a uniform spatial intensity across the hydrogel, demonstrating that drug compounds can effectively permeate the 3D bioprinted model (Fig. 6a–c).

EGFR is commonly amplified, overexpressed, or mutated in glioblastoma, so we evaluated the treatment efficacy of two EGFR inhibitors, erlotinib and gefitinib, and the glioblastoma standard-of-care alkylating agent temozolomide in our models. 3D tri-cultures and tetra-cultures were cultured for 5 days before drug treatment. Despite activated EGFR in glioblastomas, EGFR inhibitors have shown little benefit for glioblastoma patients. GSC23 in either 3D model displayed enhanced resistance to EGFR inhibitors and temozolomide compared to sphere culture. Inclusion of M2 macrophage further increased resistance of GSC23 to EGFR inhibitors (Fig. 6d; Supplementary information, Fig. S12). CW468 cultured in 3D models displayed enhanced resistance to erlotinib and temozolomide treatment, in contrast to gefitinib (Fig. 6e; Supplementary information, Fig. S12), despite maintaining high EGFR mRNA and protein expression in tetra-cultures (Supplementary information, Fig. S13). Both erlotinib and gefitinib displayed on target effects and reduced EGFR activity as measured by phosphorylation of the EGFR-Y1173 residue in both sphere culture and in tetra-cultures (Supplementary information, Fig. S14).

Glioblastomas are highly lethal cancers for which current therapy is palliative.^47,48^ Therefore, we explored the potential utility of 3D bioprinted systems to inform drug responses in glioblastoma. Overlaying gene expression data from the 3D tetra-culture model with drug sensitivity and gene expression data from the Cancer Cell Line Encyclopedia (CCLE) and the Cancer Therapeutic Response Platform (CTRP) enabled prediction of drug sensitivity and resistance in our 3D tetra-culture model based on transcriptional signatures (Fig. 6f).^49–51^ Consistent with our studies of erlotinib, gefitinib, and temozolomide, high expression of genes upregulated in GSCs in the 3D tetra-culture model was predicted to be associated with drug resistance for the majority of compounds across all cancer cell lines tested (Fig. 6g) or when restricted to brain cancer cell lines (Supplementary information, Fig. S15a). Drugs predicted to be ineffective included GSK-J4 (JMJD3/KDM6B inhibitor), cytarabine (nucleotide antimetabolite), and decitabine (DNA methyltransferase inhibitor), while drugs predicted to be effective included abiraterone (CYP17A1 inhibitor), vemurafenib and PLX-4720 (RAF inhibitors), ML334 (NRF2 activator), and ifosfamide (akylating agent) (Fig. 6g–j). The drug sensitivity predictions were similar, but not entirely overlapping, when a glioblastoma orthotopic xenograft expression signature was used (Supplementary information, Fig. S15b). Investigation of the Library of Integrated Network-Based Cellular Signatures (LINCS) dataset^52^ showed that compounds predicted to recapitulate the 3D tetra-culture signature included hypoxia inducible factor activators, caspase activators, and HDAC inhibitors, while RAF inhibitors and immunosuppressive agents may impair expression of this gene signature (Supplementary information, Fig. S15c). These findings suggest that interactions with the local microenvironment affect GSC sensitivity to therapeutic compounds and that the 3D bioprinted tissue model can interrogate these context-dependent effects. Further, as the tetra-culture model expresses genes associated with poor sensitivity to a variety of therapeutic compounds, this system may be a more realistic model for drug discovery in glioblastoma. To validate these predictions, we treated GSCs with three of the predicted compounds, abiraterone, vemurafenib, and ifosfamide in tri-culture and tetra-culture bioprinted models. When treated at the sphere culture IC_50_ value (Supplementary information, Fig. S15d–f), GSCs in tetra-culture displayed enhanced sensitivity to abiraterone and ifosfamide compared to GSCs in tri-culture, while sensitivity to vemurafenib was unchanged (Fig. 6i–k). This suggests that abiraterone and ifosfamide may be effective in targeting tetra-culture derived GSCs. Further validating these findings in an in vivo subcutaneous glioblastoma xenograft model, ifosfamide therapy reduced tumor growth compared to vehicle (Supplementary information, Figs. S16a–c).

### 3D bioprinted tissues uncover novel context-dependent essential pathways and serve as a platform for CRISPR screening

**Fig. 7:**
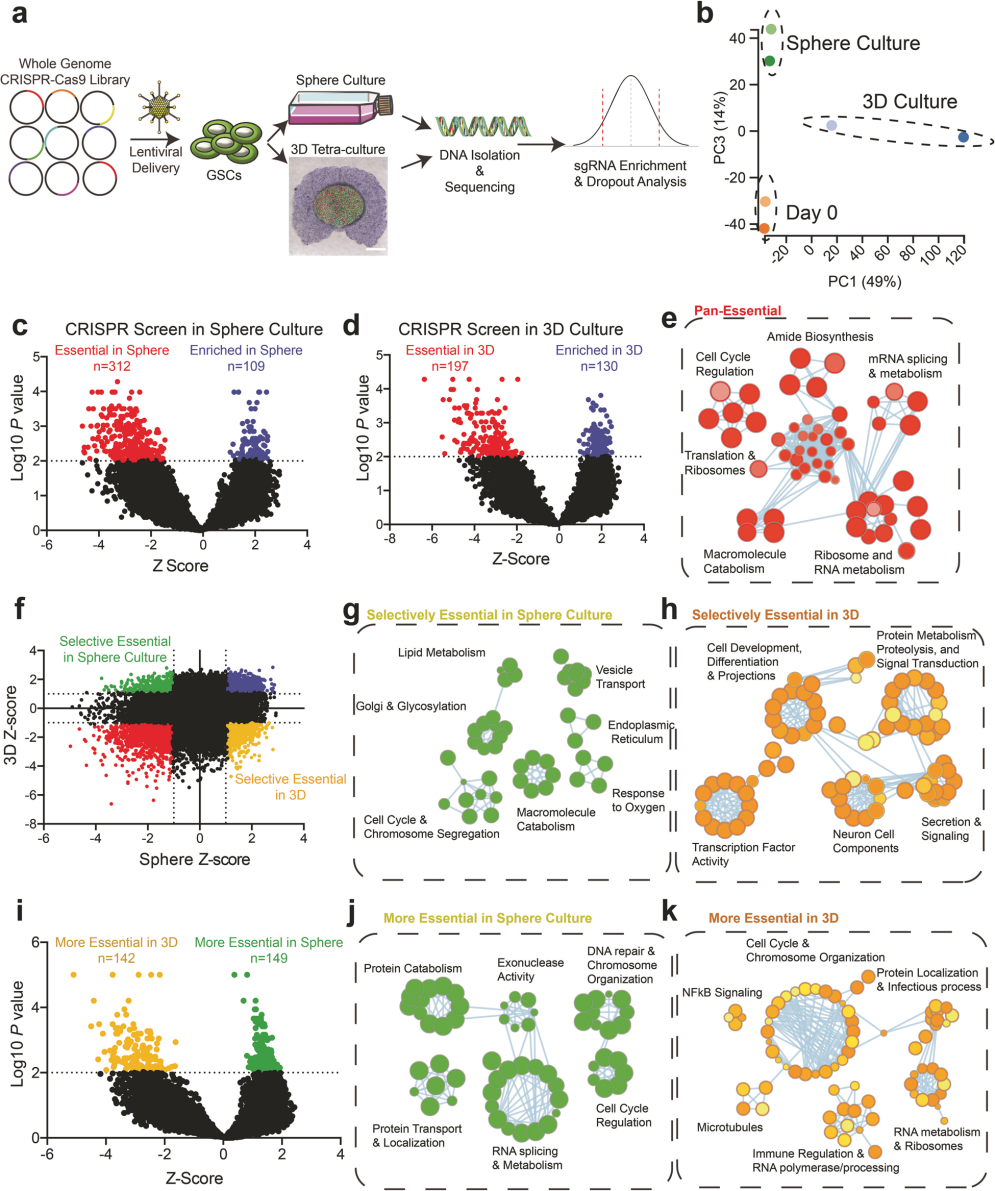
Whole-genome CRISPR-Cas9 screen reveals context-specific functional dependencies. **a** Schematic diagram of whole-genome CRISPR-Cas9 loss-of-function screening strategy in standard sphere culture conditions and the 3D tetra-culture model. **b** PCA of functional dependencies defined by whole genome CRISPR-Cas9 screening as defined in (**a**). **c** Volcano plot demonstrating genes that enhance (blue) or inhibit (red) cell proliferation in sphere culture when inactivated by a specific sgRNA in a whole genome CRISPR-Cas9 loss-of-function screen. The x-axis displays the Z-score and the y-axis displays the *P* value as calculated by the MAGECK-VISPR algorithm. **d** Volcano plot demonstrating genes that enhance (blue) or inhibit (red) cell proliferation in the 3D tetra-culture model when inactivated by a specific sgRNA in a whole genome CRISPR-Cas9 loss-of-function screen. The x-axis displays the Z-score and the y-axis displays the *P* value as calculated by the MAGECK-VISPR algorithm.^83^ **e** Pathway gene set enrichment connectivity diagram displaying pathways enriched among functional dependency genes common to both sphere culture and 3D culture in the tetra-culture model. **f** Plot comparing the functional dependency Z-scores between sphere culture and 3D culture in the tetra-culture model. **g** Pathway gene set enrichment connectivity diagram displaying pathways enriched among functional dependency genes that are specific to sphere culture, as defined in **f**. **h** Pathway gene set enrichment connectivity diagram displaying pathways enriched among functional dependency genes that are specific to growth in the 3D tetra-culture, as defined in **f**. **i** Volcano plot displaying differential functional dependency scores between sphere culture and the 3D tetra-culture system as defined by MAGECK-VISPR.^83^ **j** Pathway gene set enrichment connectivity diagram displaying pathways enriched among functional dependency genes that are more essential in sphere culture compared to in the 3D tetra-culture system, as defined in **i**. **k** Pathway gene set enrichment connectivity diagram displaying pathways enriched among functional dependency genes that are more essential in the 3D tetra-culture system compared to in sphere culture, as defined in **i**.

Given widespread therapeutic resistance in glioblastoma, we leveraged the 3D bioprinted construct as a discovery platform for glioblastoma dependencies. Parallel whole-genome CRISPR-Cas9 loss-of-function screening was performed in GSCs in sphere culture as well as in the 3D tetra-culture system (Fig. 7a; Supplementary information, Fig. S17). Functional dependencies segregated GSCs based on their method of growth (Fig. 7b; Supplementary information, Fig. S17f). Guide RNAs were enriched (indicating that the targeted gene enhances viability when deleted) or depleted (indicating that the targeted gene reduces cell viability when deleted) in each platform (Fig. 7c, d). Genes essential in each context, as well as pan-essential genes common to both platforms, included core pathways involved in translation, ribosome functions, and RNA processing, cell cycle regulation, protein localization, and chromosomes and DNA repair (Fig. 7e; Supplementary information, Fig. S17g, h). Gene hits were stratified to identify context-specific dependencies (Fig. 7f). Genes selectively essential in sphere culture were enriched for cell cycle, endoplasmic reticulum, golgi and glycosylation, lipid metabolism, and response to oxygen pathways. GSCs grown in the 3D tetra-culture model were more dependent on transcription factor activity, cell development and differentiation, NF-κB signaling, and immune regulation pathways (Fig. 7g–k). Thus, the 3D bioprinted model allowed for interrogation of functional dependencies of brain tumor cells in physiological settings and in combination with stromal fractions and revealed a more complex functional dependency network than that observed in sphere culture.

**Fig. 8:**
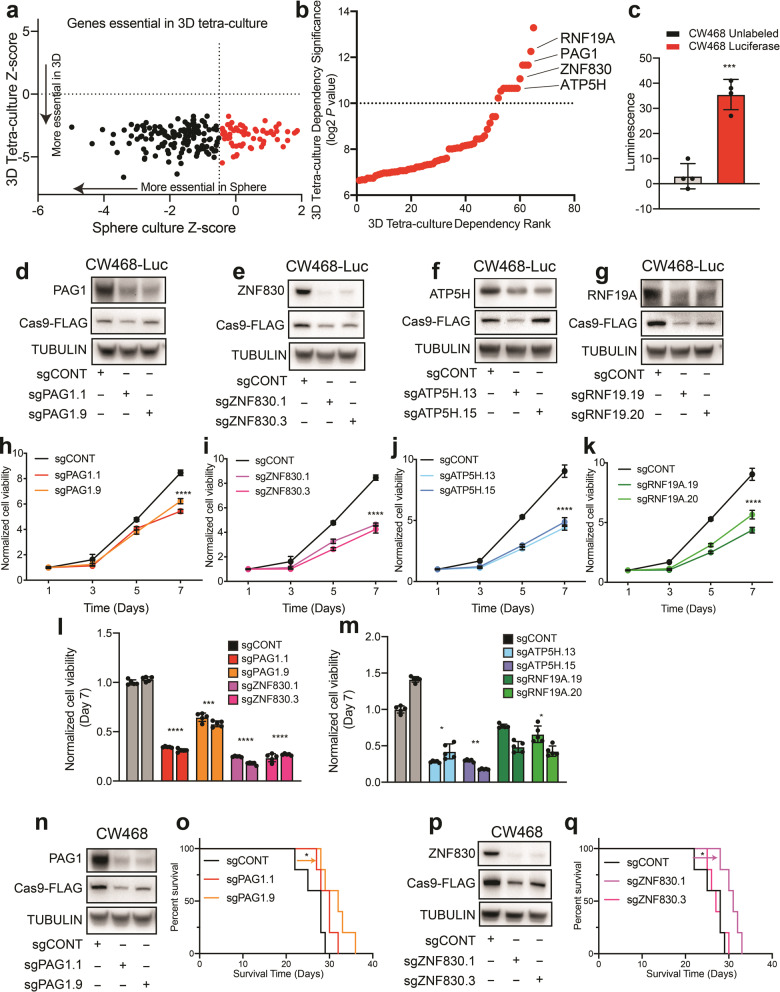
PAG1 and ZNF830 are potential therapeutic targets in glioblastoma. **a** 3D tetra-culture specific target identification approach. Graph showing gene dependency z-score in sphere culture (x-axis) vs tetra-culture (y-axis). Red color indicates genes with a sphere culture z-score of > −0.5 and a tetra-culture z-score of < −0.5. **b** Red genes from (**a**) ranked based on the dependency significance in tetra-culture models (−log2 of the *P* value). **c** Luminescent signal in GSCs transfected with a luciferase expression vector (red) or un-transfected cells following treatment with luciferin reagent for 10 min. ***P < 0.001. Unpaired, two-tailed *t* test was used for statistical analysis. **d** Western blot for PAG1 and FLAG-tagged Cas9 following treatment with two independent sgRNAs targeting PAG1 in luciferase-expressing CW468 cells or a non-targeting control (sgCONT). Tubulin was used as a loading control. **e** Western blot for ZNF830 and FLAG-tagged Cas9 following treatment with two independent sgRNAs targeting ZNF830 in luciferase expressing CW468 cells or a sgCONT. Tubulin was used as a loading control. **f** Western blot for ATP5H (ATP5PD) and FLAG-tagged Cas9 following treatment with two independent sgRNAs targeting ATP5H in luciferase expressing CW468 cells or a sgCONT. Tubulin was used as a loading control. **g** Western blot for RNF19A and FLAG-tagged Cas9 following treatment with two independent sgRNAs targeting RNF19A in luciferase expressing CW468 cells or a sgCONT. Tubulin was used as a loading control. **h** Cell viability of CW468 luciferase expressing GSCs in sphere culture following treatment with two independent sgRNAs targeting PAG1 or a sgCONT. ****P < 0.0001. Two-way repeated measures ANOVA with Dunnett multiple comparison testing was used for statistical analysis. **i** Cell viability of CW468 luciferase expressing GSCs in sphere culture following treatment with two independent sgRNAs targeting ZNF830 or a sgCONT. ****P < 0.0001. Two-way repeated measures ANOVA with Dunnett multiple comparison testing was used for statistical analysis. **j** Cell viability of CW468 luciferase-expressing GSCs in sphere culture following treatment with two independent sgRNAs targeting ATP5H or a sgCONT. ****P < 0.0001. Two-way repeated measures ANOVA with Dunnett multiple comparison testing was used for statistical analysis. **k** Cell viability of CW468-luciferase expressing GSCs in sphere culture following treatment with two independent sgRNAs targeting RNF19A or a sgCONT. ****P < 0.0001. Two-way repeated measures ANOVA with Dunnett multiple comparison testing was used for statistical analysis. **l** Cell viability of CW468 luciferase expressing GSCs in 3D tetra-culture models after editing with two independent sgRNAs targeting PAG1, ZNF830, or a non-targeting sgRNA after seven days. ****P < 0.0001. Bars show mean and standard deviation of two biological replicates with 5 technical replicates. Ordinary one-way ANOVA with Dunnett multiple comparison correction was used for statistical analysis. **m** Cell viability of CW468 luciferase expressing GSCs in 3D tetra-culture models after editing with two independent sgRNAs targeting ATP5H, RNF19A, or a non-targeting sgRNA after seven days. *P < 0.05; **P < 0.01. Bars show mean and standard deviation of two biological replicates with 5 technical replicates. Ordinary one-way ANOVA with Dunnett multiple comparison correction was used for statistical analysis. **n** Western blot for PAG1 and FLAG-tagged Cas9 following treatment with two independent sgRNAs targeting PAG1 in CW468 GSCs or a sgCONT. Tubulin was used as a loading control. **o** Kaplan–Meier plot showing mouse survival following orthotopic implantation of GSCs edited with one of two sgRNAs targeting PAG1 or a sgCONT. sgPAG1.1 vs sgCONT, *P* = 0.071. sgPAG1.9 vs sgCONT = 0.023. Log-rank test was used for statistical analysis. **p** Western blot for ZNF830 and FLAG-tagged Cas9 following treatment with two independent sgRNAs targeting ZNF830 in CW468 GSCs or a sgCONT. Tubulin was used as a loading control. **q** Kaplan–Meier plot showing mouse survival following orthotopic implantation of GSCs edited with one of two sgRNAs targeting ZNF830 or a sgCONT. sgZNF830.1 vs sgCONT, *P* = 0.011. sgZNF830.3 vs sgCONT, *P* > 0.05. Log-rank test was used for statistical analysis.

To further validate 3D bioprinted-specific dependencies, we stratified our whole-genome CRISPR screening results, selecting genes predicted to be essential in 3D tetra-culture (Fig. 8a, b). Individual gene knockout in luciferase-labeled GSCs of PAG1, ZNF830, ATP5H, and RNF19A with two independent sgRNAs reduced GSC viability in both sphere culture and 3D tetra-culture models (Fig. 8c–m). Additionally, knockout of PAG1 or ZNF830 in GSCs delayed the onset of neurological signs in orthotopic glioblastoma xenografts compared to GSCs treated with a non-targeting sgRNA (Fig. 8n–q). PAG1 and ZNF830 are upregulated at the mRNA level in glioblastomas compared to normal brain tissue and high expression is associated with poor patient prognosis in primary glioblastomas from the Chinese Glioma Genome Atlas (CGGA) dataset, highlighting the clinical relevance of these factors in glioblastoma (Supplementary information, Fig. S18a–d). Taken together, this screening approach has identified novel candidates for future investigation and potential therapeutic development.

### 3D bioprinted cultures express transcriptional signatures associated with poor glioblastoma patient prognosis

**Fig. 9:**
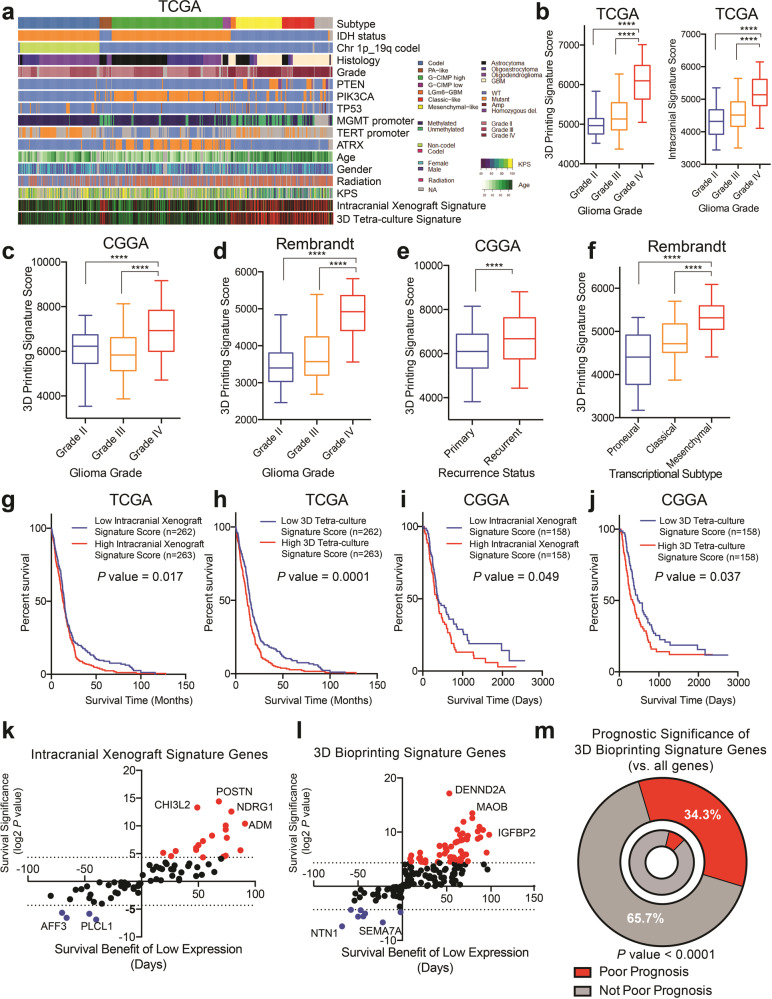
3D bioprinting contributes to upregulation of genes with poor prognostic significance in glioblastoma. **a** Heatmap displaying mRNA expression signatures of intracranial xenografts (vs sphere cell culture) and 3D bioprinted tetra-cultures (vs sphere cell culture) as defined by the TCGA glioma HG-U133A microarray. Various clinical metrics, patient information and information on tumor genetics are also displayed. **b** mRNA expression signature of (Left) 3D bioprinted tetra-cultures (vs sphere cell culture) and (Right) intracranial xenografts (vs sphere cell culture) in TCGA glioma HG-U133A microarray. Grade II (*n* = 226), Grade III (*n* = 244), Grade IV (*n* = 150). The box-and-whisker plot indicates the lower quartile, median, and upper quartile. Error bars represent the 5%–95% confidence interval. Ordinary one-way ANOVA with Tukey multiple comparison test was used for statistical analysis, ****P < 0.0001. **c** mRNA expression signature of 3D bioprinted tetra-cultures (vs sphere cell culture) in CGGA. Grade II (*n* = 188), Grade III (*n* = 255), Grade IV (*n* = 249). The box-and-whisker plot indicates the lower quartile, median, and upper quartile. Error bars represent the 5%–95% confidence interval. Ordinary one-way ANOVA with Tukey multiple comparison test was used for statistical analysis, ****P < 0.0001. **d** mRNA expression signature of 3D bioprinted tetra-cultures (vs sphere cell culture) in the Rembrandt glioma dataset. Grade II (*n* = 98), Grade III (*n* = 85), Grade IV (*n* = 130). The box-and-whisker plot indicates the lower quartile, median, and upper quartile. Error bars represent the 5%–95% confidence interval. Ordinary one-way ANOVA with Tukey multiple comparison test was used for statistical analysis, ****P < 0.0001. **e** mRNA expression signature of 3D bioprinted tetra-cultures (vs sphere cell culture) in the Chinese Glioma Genome Atlas (CGGA). Data presented is restricted to glioblastomas (grade IV glioma). Primary (*n* = 422), Recurrent (*n* = 271). The box-and-whisker plot indicates the lower quartile, median, and upper quartile. Error bars represent the 5%–95% confidence interval. Ordinary one-way ANOVA with Tukey multiple comparison test was used for statistical analysis, ****P < 0.0001. **f** mRNA expression signature of 3D bioprinted tetra-cultures (vs sphere cell culture) in the Rembrandt glioma dataset. Data presented is restricted to glioblastomas (grade IV glioma). Proneural (*n* = 41), Mesenchymal (*n* = 44), Classical IV (*n* = 45). The box-and-whisker plot indicates the lower quartile, median, and upper quartile. Error bars represent the 5%–95% confidence interval. Ordinary one-way ANOVA with Tukey multiple comparison test was used for statistical analysis, ****P < 0.0001. **g** Kaplan–Meier survival analysis of glioblastoma patients in the TCGA dataset based on the mRNA expression signature of intracranial xenografts (vs sphere cell culture). Patients were grouped into “high” or “low” signature expression groups based on the median signature expression score. Low (*n* = 262), high (*n* = 263). Log rank analysis was used for statistical analysis, *P* = 0.017. **h** Kaplan–Meier survival analysis of glioblastoma patients in the TCGA dataset based on the mRNA expression signature of 3D bioprinted tetra-cultures (vs sphere cell culture). Patients were grouped into “high” or “low” signature expression groups based on the median signature expression score. Low (*n* = 262), high (*n* = 263). Log rank analysis was used for statistical analysis, *P* = 0.0001. **i** Kaplan–Meier survival analysis of glioblastoma patients in the CGGA dataset based on the mRNA expression signature of intracranial xenografts (vs sphere cell culture). Patients in the top 1/3 of the expression signature score were grouped into the “high” group, while those in the bottom 1/3 of the expression signature score were grouped into the “low” group. Low (*n* = 158), high (*n* = 158). Log rank analysis was used for statistical analysis, *P* = 0.017. **j** Kaplan–Meier survival analysis of glioblastoma patients in the CGGA dataset based on the mRNA expression signature of 3D bioprinted tetra-cultures (vs sphere cell culture). Patients in the top 1/3 of the expression signature score were grouped into the “high” group, while those in the bottom 1/3 of the expression signature score were grouped into the “low” group. Low (*n* = 158), high (*n* = 158). Log rank analysis was used for statistical analysis, *P* = 0.0001. **k** Plot showing genes in the intracranial xenograft signature ranked by (x-axis) the mean survival difference between the “high” expressing group and the “low” expressing group and (y-axis) the statistical significance of the survival difference as calculated by the log-rank test. Patients were grouped into “high” or “low” signature expression groups based on the median gene expression. **l** Plot showing genes in the 3D bioprinted tetra-cultures (vs sphere cell culture) signature ranked by (x-axis) the mean survival difference between the “high” expressing group and the “low” expressing group and (y-axis) the statistical significance of the survival difference as calculated by the log-rank test. Patients were grouped into “high” or “low” signature expression groups based on the median gene expression. **m** The outer pie chart displays the fraction of genes with prognostic significance in the 3D bioprinted tetra-cultures gene signature as calculated by the log-rank test. Patients were grouped into “high” or “low” signature expression groups based on the median gene expression. The inner pie chart displays the number of total prognostically significant genes as a fraction of all genes. The Chi-squared test was used for statistical analysis, *P* < 0.0001.

To determine the clinical relevance of the 3D bioprinted construct, we investigated the transcriptional profiles relative to glioblastoma patients. Signatures of genes upregulated either in intracranial orthotopic xenografts or in 3D tetra-culture compared to sphere culture were elevated in glioblastomas compared to low-grade gliomas in The Cancer Genome Atlas (TCGA), CGGA, and the Rembrandt dataset (Fig. 9a–d). The 3D tetra-culture gene signature was elevated in recurrent glioblastomas compared to primary tumors (Fig. 9e) and in the mesenchymal subtype compared to classical or proneural glioblastomas (Fig. 9f). In the TCGA and CGGA datasets, the orthotopic xenograft signature and the 3D tetra-culture signature were associated with poor glioblastoma patient prognosis (Fig. 9g–j). Many genes with individual poor prognostic significance were upregulated in the intracranial xenograft signature, including CHI3L2, POSTN, and NDRG1 (Fig. 9k), while DENND2A, MAOB, and IGFBP2 were upregulated in the 3D bioprinted cultures (Fig. 9l). Genes with poor prognostic significance were enriched among all genes in the 3D tetra-culture signature, when compared to a background of all genes (Fig. 9m). Thus, 3D bioprinting enabled investigation of gene pathways associated with more aggressive glioblastomas, suggesting that this model can serve as a more realistic therapeutic discovery platform for the most lethal classes of glioblastoma.

## Discussion

To improve modeling of a highly lethal brain cancer for which current therapies are limited, we utilized a DLP-based 3D bioprinting system to model glioblastoma, the most common and highly lethal type of brain tumor. Studies have reported using 3D printing to create coculture models of glioblastoma cells with other stromal cells or fabricate HA-based hydrogel to mimic brain ECM.^23,24,53^ However, most prior models focus on only one aspect of the in vivo situation or used non-human cells, which reduced their capacity to be applied to actual clinical settings. To the best of our knowledge, this is the first report of a human cell-based 3D glioblastoma model that recapitulates the complex tumor microenvironment with inclusion of normal brain, immune components, stromal components, and essential mechanical and biochemical cues from the extracellular matrix.

The tumor microenvironment provides essential signals to guide tumor growth and survival; however, these cues are inefficiently modeled in standard 2D culture, even in the absence of serum. Hypoxic signaling contributes to glioblastoma aggressiveness by remodeling GSC phenotypes.^54,55^ Our 3D tetra-culture brain tumor model expressed hypoxia response signatures, allowing for investigation of hypoxic signaling in a physiologic environment, unlike standard cell culture systems. Critical growth factor signaling elements are provided from neurons,^43–45,56,57^ NPCs,^58^ ECM components,^59,60^ and immune fractions, including macrophages.^61,62^ The perivascular niche provides a variety of signals including Wnts,^63^ ephrins,^64^ and osteopontins^65^ to promote glioblastoma invasion, growth, and maintenance of GSCs. Future studies will be required to integrate vascular components into the 3D printed model system to further study these important components of the brain tumor microenvironment. The 3D tetra-culture tissue environment presented here enables controlled, reproducible, and scalable interrogation of these various cellular interactions that drive brain tumor biology. While microenvironmental components supply critical niche factors to sustain the tumor ecosystem, stromal elements are also actively remodeled by malignant cells.^66^ Here, we observed the role of immune cells in glioblastoma growth, including changes in gene expression, invasive behaviors, and response to treatments. Reciprocally, we also find that the 3D glioblastoma microenvironment promoted polarization of macrophages towards a protumoral M2 macrophage phenotype, highlighting this bidirectional crosstalk.

The bioprinting approach generates a spatially separated tumor region and surrounding non-neoplastic neural tissue with defined cell density which allows the cells to interact in a more realistic manner, providing a highly reproducible platform for the interrogation of cell-cell interactions with several key advantages. First, this 3D glioblastoma tissue model allows for investigation of tumor–immune interactions in a fully human species-matched system, which is not possible in xenograft or genetically engineered mouse model. This may facilitate understanding of human-specific immune interactions and advance the field of neuro-oncoimmunology by providing insights into immunotherapy efficacy. Second, combining tumoral and non-neoplastic neural components within one model will propel drug discovery efforts by enabling measurements of therapeutic efficacy, toxicities, and therapeutic index. The scalability and reproducibility of this 3D bioprinted model also allows for more high-throughput compound screening efforts. Our findings suggest that the 3D bioprinted model displays transcriptional signatures closer to patient-derived glioblastoma tissue, and that local stromal interactions present within our model promotes broad therapeutic resistance, enabling compound discovery efforts in a challenging environment. Third, the 3D bioprinted model is amenable to large-scale whole-genome CRISPR-Cas9-based screening methods to uncover novel functional dependencies in a physiologic setting. This model extends previous approaches by characterizing context-dependent target essentiality in cancer cells and allowing for investigation of multivalent stromal cell dependencies.

In conclusion, we report a controlled, reproducible, and scalable 3D engineered glioblastoma tissue construct that serves as a more physiologically accurate brain tumor model, facilitates interrogation of the multicellular interactions that drive brain tumor biology, and acts as a platform for discovery of novel functional dependencies.

## Materials and methods

### GelMA and GMHA synthesis and characterization

GelMA and GMHA were synthesized using Type A, gel strength 300 gelatin from porcine skin (Sigma Aldrich Cat #: G2500) and 200,000 Da hyaluronic acid (Lifecore), respectively, as described previously.^67,68^ Briefly, for the GelMA synthesis of 95% degree of methacrylation, 10% (w/v) gelatin was dissolved in 0.25 M 3:7 carbonate-bicarbonate buffer solution (pH ~9) at 50 °C. Methacrylic anhydride was added dropwise at a volume of 0.1 mL/(gram gelatin). The reaction was left to run for 1 h at 50 °C. After synthesis, the solutions were dialyzed, frozen overnight at −80 °C, and lyophilized. Freeze-dried GelMA and GMHA were stored at −80 °C and reconstituted immediately before printing to stock solutions of 20% (w/vol) and 4% (w/vol), respectively. All materials were sterilized by syringe filters before mixing with cells (Millipore). The degree of methacrylation of GelMA and GMHA were quantified using proton NMR (Bruker, 600 MHz).

### Cell culture

Xenografted tumors were dissociated using a papain dissociation system according to the manufacturer’s instructions. GSCs were then cultured in Neurobasal medium supplemented with 2% B27, 1% L-glutamine, 1% sodium pyruvate, 1% penicillin/streptomycin, 10 ng/mL basic human fibroblast growth factor (bFGF), and 10 ng/mL human epidermal growth factor (EGF) for at least 6 h to recover expression of surface antigens. GSC phenotypes were validated by expression of stem cell markers (SOX2 and OLIG2) functional assays of self-renewal (serial neurosphere passage), and tumor propagation using in vivo limiting dilution.

THP-1 monocytes were cultured in RPMI 1640 (Gibco) medium supplemented with 10% heat-inactivated fetal bovine serum (FBS, Invitrogen) and 1% penicillin/streptomycin. To obtain monocyte-derived M2 macrophage, THP-1 monocytes were first seeded in 6-well plates at a density of 5 × 10^5^ cells/mL (3 mL/well). Polarization to M2 macrophage was induced by (1) incubating cells in 200 ng/mL phorbol 12-myristate 13-acetate (PMA, Sigma Aldrich) for 48 h, (2) replacing with THP1 complete medium for 24 h, and then (3) incubating in 20 ng/mL interleukin 4 (IL4, Peprotech) and 20 ng/mL interleukin 13 (IL13, Peprotech) for 48 h. hNP1 neural progenitor cells (Neuromics) were cultured on Matrigel-coated plates using the complete NBM medium for GSCs. Human astrocytes (ThermoFisher) were cultured with astrocyte medium (ScienCell) supplemented with 1% penicillin/streptomycin.

### 3D bioprinting process

Before printing, GSCs, hNP1s, and astrocytes were digested by Accutase (Stemcell Technology), and macrophages were digested with TrypLE (ThermoFisher). For the 3D tetra-culture samples, the cell suspension solution for the tumor core consisted of 2.5 × 10^7^ cells/mL GSCs and 1.25 × 10^7^ cells/mL macrophages (GSCs:M2 = 2:1). For the 3D tri-culture samples, the core cell suspension solution consisted of 2.5 × 10^7^ cells/mL GSCs only (Supplementary information, Fig. S2a, b). The cell suspension solution for the peripheral region for both models consisted of 1 × 10^7^ cells/mL hNP1s and 1 × 10^7^ cells/mL astrocytes. All cell suspensions were aliquoted into 0.5 ml Eppendorf tubes and stored on ice before use. The prepolymer solution for bioprinting was prepared with 8% (w/v) GelMA, 0.5% (w/v) GMHA, and 0.6% (w/v) lithium phenyl(2,4,6-trimethylbenzoyl)phosphinate (LAP) (Tokyo Chemical Industry). Prepolymer solution was kept at 37 °C in dark before use. Cell suspension was mixed with prepolymer solution at 1:1 ratio immediately before printing to maximize viability.

The two-step bioprinting process utilized a customized light-based 3D printing system. Components of the system included a digital micromirror device (DMD) chip (Texas Instruments), a motion controller (Newport), a light source (Hamamatsu), a printing stage, and a computer with software to coordinate all the other components. The thickness of the printed samples was precisely controlled by the motion controller and the stage. Cell-material mixture was loaded onto the printing stage, and the corresponding digital mask was input onto the DMD chip. Light was turned on for an optimized amount of exposure time (20 s for the core and 15 s for the periphery). The bioprinted 3D tri-culture/tetra-culture samples were then rinsed with DPBS and cultured in maintenance medium at 37 °C with 5% CO_2_. Maintenance medium was made of 50% of complete NBM medium, 25% of THP1 medium, and 25% of astrocyte medium.

### hiPSC-derived macrophage generation

hiPSC-derived macrophage differentiation protocol was adapted from Yanagimachi et al.^69^ and modified from Mesci et al.^38^ Briefly, iPSC cell lines were generated as previously described, by reprogramming fibroblast from a healthy donor.^70^ The iPSC colonies were plated on Matrigel-coated (BD Biosciences) plates and maintained in mTESR media (Stem Cell Technologies). The protocol of myeloid cell lineage consisted of 4 sequential steps. In the first step, primitive streak cells were induced by BMP4 addition, which in step 2, were differentiated into hemangioblast-like hematopoietic precursors (VEGF (80 ng/mL, Peprotech), SCF (100 ng/mL, Gemini) and basic fibroblast growth factor (bFGF), (25 ng/mL, Life Technologies)). Then, in the third step, the hematopoietic precursors were pushed towards myeloid differentiation (FLT-3 ligand (50 ng/mL, HumanZyme), IL-3 (50 ng/mL, Gemini), SCF (50 ng/mL, Gemini), Thrombopoietin, TPO (5 ng/mL), M-CSF (50 ng/mL)) and finally into the monocytic lineage in step 4 [FLT3-ligand (50 ng/mL), M-CSF (50 ng/mL), GM-CSF (25 ng/mL)]. Cells produced in suspension in step 4 were recovered, sorted by using anti-CD14 magnetic microbeads (MACS, Miltenyi) and then integrated into 3D bioprinted models as described above.

### Isolation and generation of primary human macrophages

Human blood was obtained from healthy volunteers from the Scripps Research Institute Normal Blood Donor service. Mononuclear cells were isolated by gradient centrifugation using Lymphoprep (#07851 STEMCELL), washed with PBS, and treated with red blood cell lysis buffer. Cells were plated to adhere monocytes and cultured in 10% heat inactivated FBS in RPMI with HEPES, GlutaMAX, 1 mM Sodium Pyruvate, and Pen/Strep with 50 ng/mL M-CSF for 6 days as described by Ogasawara et al.^71^ Unpolarized M0 macrophages were collected and integrated into 3D bioprinted models as described above.

### Mechanical testing

Compressive modulus of the 3D printed constructs was measured with a MicroSquisher (CellScale). Pillars with 1 mm in diameter and 1 mm in height were printed with same conditions used for the tissue models and incubated overnight at 37 °C. Both acellular and cell-encapsulated constructs were tested. The MicroSquisher utilized stainless steel beams and platens to compress the constructs at 10% displacement of their height. Customized MATLAB scripts were used to calculate the modulus from the force and displacement data collected by MicroSquisher.

### SEM

Surface patterns of the materials and cell-material interactions on micron-scale were imaged with a scanning electron microscope (Zeiss Sigma 500). Acellular samples were snap-frozen in liquid nitrogen and immediately transferred to the freeze drier to dry overnight. Cell-encapsulated samples were dried based on a chemical dehydration protocol. Briefly, samples were fixed using 2.5% glutaraldehyde solution for 1 h at room temperature and then overnight at 4 °C. On the next day, the samples were rinsed with DPBS for three times and soaked in 70% ethanol, 90% ethanol, and 95% ethanol subsequently, each for 15 min. Then the solution was replaced with 100% ethanol for 10 min, and the step was repeated two more times. Hexamethyldisilazane (HDMS) was mixed with 100% ethanol at 1:2 ratio and 2:1 ratio. Samples were first transferred to HDMS:EtOH (1:2) for 15 min, then HDMS:EtOH (2:1) for 15 min. Then the solution was replaced with 100% HDMS for 15 min, and the step was repeated two more times. The samples were left uncovered in chemical hood overnight to dry. The freeze-dried or chemically dried samples were coated with iridium by a sputter coater (Emitech) prior to SEM imaging.

### Immunofluorescence staining and image acquisition of tumor model

3D bioprinted samples and sphere cultured cells were fixed with 4% paraformaldehyde (PFA; Wako) for 30 min and 15 min, respectively, at room temperature. All samples were blocked and permeabilized using 5% (w/v) bovine serum albumin (BSA, Gemini Bio-Products) solution with 0.1% Triton X-100 (Promega) for 1 h at room temperature on a shaker. Samples were then incubated with the respective primary antibody (listed below) overnight at 4 °C. On the next day, samples were rinsed by DPBS with 0.05% Tween 20 (PBST) for three times on the shaker. Samples were incubated with fluorophore-conjugated goat anti-rabbit or goat anti mouse secondary antibodies (1:200; Biotium) and Hoechst 33342 (1:1000; Life Technologies) counterstain in DPBS with 2% (w/v) BSA for 1 h at room temperature in dark. After incubation, samples were rinsed three times in PBST and stored in DPBS with 0.05% sodium azide (Alfa Aesar) at 4 °C before imaging. Fluorescence images of 3D samples and their sphere cultured counterparts were taken with a confocal microscope (Leica SP8) using consistent settings for each antibody (Supplementary Information, Table S1).

Fluorescence images of EGFP- or mCherry-abeled cells in the 3D samples were also acquired using the confocal microscope. Tile scan merging was completed by the automated program on the Leica microscope and the z-stack projection was completed by ImageJ. Quantification of the migration was based on the fluorescence images processed by ImageJ.

### RNA isolation and RT-PCR

EGFP-labeled GSCs and mCherry-labeled THP1s were isolated from 3D printed tri-culture and tetra-culture samples using flow cytometry (BD FACSAria II). Cells isolated from 3D and sphere cultured cells were treated with TRIzol reagent (Life Technologies) before RNA extraction. Total RNA of each sample was extracted using Direct-zol RNA MiniPrep Kit (Zymo) and immediately stored at −80 °C. To perform RT-PCR, cDNA was first obtained by RNA reverse transcription using the ProtoScript® First Strand cDNA Synthesis Kit (New England BioLabs) with input RNA of 200 ng per sample. The primers were purchased from Integrated DNA Technologies. RT-PCR was performed using PowerUp SYBR Green master mix (Applied Biosystems) and detected with Quantstudio 3 RT-PCR system. Gene expression was determined by the threshold cycle (Ct) values normalized against the housekeeping gene (Supplementary information, Table S2).

### RNA-seq and data analysis

RNA was purified as described above and subjected to RNA-seq. Paired-end FASTQ sequencing reads were trimmed using Trim Galore version 0.6.2 (https://www.bioinformatics.babraham.ac.uk/projects/trim_galore/) using cutadapt version 2.3. Transcript quantification was performed using Salmon^72^ version 0.13.1 in the quasi-mapping mode from transcripts derived from human Gencode release 30 (GRCh38.12).^73^ Salmon “quant” files were converted using Tximport^74^ (https://bioconductor.org/packages/release/bioc/html/tximport.html) and differential expression analysis was performed using DESeq2^75^ in the R programming language. Data from GSCs and primary glioblastoma surgical resection tissues were derived from Mack et al.^37^ and were processed using the same analysis pipeline. Data from matched GSCs grown in serum-free sphere culture and orthotopic intracranial xenografts were derived from Miller et al.^36^ and were processed using the same analysis pipeline. Processed data from matched GSCs and differentiated tumor cells were derived from Suva et al.^76^ and differentially expressed genes were calculated using the Limma-Voom algorithm in the Limma package^77^ in the R programming language.

PCA was performed within the DESeq2 package using the top 5000 differentially expressed genes. UMAP analysis was performed using the UMAPR package (https://github.com/ropenscilabs/umapr) and uwot (https://cran.r-project.org/web/packages/uwot/index.html). For comparisons of glioblastoma tissue samples with GSCs grown in standard sphere culture, analysis parameters include: sample size of local neighborhood, number of neighbors = 40; learning rate = 0.5; Initialization of low dimensional embedding = random; metrics for computation of distance in high dimensional space = manhattan. For comparisons of GSCs derived from sphere culture or 3D bioprinted models, analysis parameters include: sample size of local neighborhood, number of neighbors = 3; Initialization of low dimensional embedding = random; metrics for computation of distance in high dimensional space = cosine.

Gene set enrichment analysis was performed using the online GSEA webportal (http://software.broadinstitute.org/gsea/msigdb/annotate.jsp) and the GSEA desktop application (http://software.broadinstitute.org/gsea/downloads.jsp).^78,79^ Pathway enrichment bubble plots were generated using the Bader Lab Enrichment Map Application^80^ and Cytoscape (http://www.cytoscape.org). Glioblastoma transcriptional subtypes were calculated using a program written by Wang et al.^81^ and implemented in R. Gene signatures were calculated using the single sample Gene Set Enrichment Analysis Projection (ssGSEAProjection) module on GenePattern (https://cloud.genepattern.org).

### CRISPR editing

CRISPR editing was performed on CW468 GSCs as well as luciferase-labeled CW468 GSCs (CW468-Luc). For unlabeled cells, sgRNAs were cloned into the LentiCRISPRV2 plasmid containing a puromycin selection marker (Addgene Plasmid #52961), while luciferase-labeled cells were edited with sgRNAs cloned into the LentiCRISPRV2 plasmid containing a hygromycin selection marker (Addgene Plasmid #98291). sgRNA sequences were chosen from the Human CRISPR Knockout Pooled Library (Brunello)^82^ (Supplementary information, Table S3).

### Western blot analysis

Cells were collected and lysed in RIPA buffer (50 mM Tris-HCl, pH 7.5; 150 mM NaCl; 0.5% NP-40; 50 mM NaF with protease inhibitors) and incubated on ice for 30 min. Lysates were centrifuged at 4 °C for 10 min at 14,000 rpm, and supernatant was collected. The Pierce BCA protein assay kit (Thermo Scientific) was utilized for determination of protein concentration. Equal amounts of protein samples were mixed with SDS Laemmli loading buffer, boiled for 10 min, and electrophoresed using NuPAGE Bis-Tris Gels, then transferred onto PVDF membranes. TBS-T supplemented with 5% non-fat dry milk was used for blocking for a period of 1 h followed by blotting with primary antibodies at 4 °C for 16 h (Supplementary information, Table S4). Blots were washed 3 times for 5 min each with TBS-T and then incubated with appropriate secondary antibodies in 5% non-fat milk in TBS-T for 1 h. For all western immunoblot experiments, blots were imaged using BioRad Image Lab software and subsequently processed using Adobe Illustrator to create the figures.

### Molecular diffusion assessment

3D printed hydrogels were printed and incubated in DPBS overnight at 37 °C. Fluorescein Isothiocyanate (FITC)-dextran with average molecular weight of 4000 Da was dissolved in DPBS at concentration of 500 µg/mL. DPBS was removed and FITC-dextran solutions were added to the wells with 3D printed hydrogels. Hydrogels were incubated in FITC-dextran solution at 37 °C for 0, 5, 15, 30, 60, and 120 min; rinsed three times with DPBS; and then imaged using a fluorescence microscope. Fluorescence intensities of the hydrogel were measured by ImageJ. The average intensities and the spatial intensities at each time point were calculated in Excel and plotted using PRISM.

### Drug response assessment

3D tri-culture/tetra-culture samples were printed as described above, with regular GSCs substituted with luciferase-labeled GSCs. 3D samples and sphere cultured cells plated on Matrigel-coated slides were treated with drugs after 5 days in culture. Drug effects were evaluated 72 h later for erlotinib and gefitinib. For temozolomide, medium was replaced with fresh medium with temozolomide 72 h after first treatment, and the drug response was evaluated 72 h after second treatment. Luciferase readings were obtained using using the Promega luciferase assay system (E1500) based on the provided protocol and a Tecan Infinite M200 plate reader. Abiraterone (HY-70013), vemurafenib (HY-12057), and ifosfamide (HY-17419), erlotinib (HY-50896), and gefitinib (HY-50895) from MedChemExpress was used to generate dose response curves in vitro.

Sphere culture cell proliferation experiments were conducted by plating cells of interest at a density of 2000 cells per well in a 96-well plate with 6 replicates. Cell Titer Glo (Promega) was used to measure cell viability. Data is presented as mean ± standard deviation.

### Drug sensitivity prediction

Therapeutic sensitivity and gene expression data were accessed through the Cancer Therapeutics Response Portal (https://portals.broadinstitute.org/ctrp/).^49–51^ Gene signature scores were calculated for each cell line in the dataset using the single sample Gene Set Enrichment Analysis Projection (ssGSEAProjection) module on GenePattern (https://cloud.genepattern.org). Gene signature score was then correlated with area under the curve (AUC) values for drug sensitivity for each compound tested. Correlation r-value was plotted and statistical analyses were corrected for multiple test correction.

### CRISPR screening and data analysis

Whole-genome CRISPR-Cas9 loss-of-function screening was performed with the Human CRISPR Knockout Pooled Library (Brunello),^82^ which was a gift from David Root and John Doench (Addgene #73178). The library was used following the instructions on Addgene website (https://www.addgene.org/pooled-library/broadgpp-human-knockout-brunello). Briefly, the library was stably transduced into GSCs by lentiviral infection with a multiplicity of infection (MOI) around 0.3–0.6, after puromycin selection, cells were propagated in either standard sphere cell culture conditions or in a 3D tetra-culture system. After 10 days, genomic DNA was extracted from GSCs and the sequencing library was generated using the protocol on Addgene website (https://media.addgene.org/cms/filer_public/61/16/611619f4-0926-4a07-b5c7-e286a8ecf7f5/broadgpp-sequencing-protocol.pdf). Sequencing quality control was performed using FASTQC (http://www.bioinformatics.babraham.ac.uk/projects/fastqc) and enrichment and dropout were calculated using the MAGECK-VISPR pipeline ^83,84^ using the MAGeCK-MLE pipeline.

### In vivo tumorigenesis assays

Intracranial xenografts experiments were generated by implanting 15,000 patient-derived GSCs (CW468) following treatment with sgRNAs targeting PAG1 or ZNF830 or a sgCONT into the right cerebral cortex of NSG mice (NOD.Cg-Prkdcscid Il2rgtm1Wjl/SzJ, The Jackson Laboratory, Bar Harbor, ME, USA) at a depth of 3.5 mm under a University of California, San Diego Institutional Animal Care and Use Committee (IACUC) approved protocol. All murine experiments were performed under an animal protocol approved by the University of California, San Diego IACUC. Healthy, wild-type male or female mice of NSG background, 4–6 weeks old, were randomly selected and used in this study for intracranial injection. Mice had not undergone prior treatment or procedures. Mice were maintained in 14 h light/10 h dark cycle by animal husbandry staff with no more than 5 mice per cage. Experimental animals were housed together. Housing conditions and animal status were supervised by a veterinarian. Animals were monitored until neurological signs were observed, at which point they were sacrificed. Neurological signs or signs of morbidity included hunched posture, gait changes, lethargy and weight loss. Survival was plotted using Kaplan–Meier curves with statistical analysis using a log-rank test.

Subcutaneous xenografts were established by implanting 2 million luciferase-labeled CW468 GSCs into the right flank of NSG mice and maintained as described above. Two weeks after implantation, treatment was initiated with 80 mg/kg of ifosfamide (HY-17419, MedChemExpress) dissolved in 90% safflower oil (Spectrum Laboratory Products) and 10% DMSO or vehicle alone by 100 μL intraperitoneal injection once per day for 28 days. Luminescence signal was assessed at days 0, 7, 14, 21, and 28 after initiation of treatment using bioluminescence imaging following injection of luciferin reagent intraperitoneally. Tumor size was normalized based on the day 7 time point for each mouse individually.

### Statistical analysis

Statistical analysis parameters are provided in each figure legend. Multiple group comparisons were compared by one-way ANOVA with Tukey’s post-hoc analysis (by GraphPad Prism). *P* < 0.05 was designated as the threshold value for statistical significance. All data were displayed as mean values with error bars representing standard deviation.

## Supplementary information

Supplementary Information

## Data Availability

All raw sequencing data and selected processed data is available on GEO at the accession number GSE147147 (https://www.ncbi.nlm.nih.gov/geo/query/acc.cgi?acc=GSE147147). There are no restrictions on data availability, and all data will be made available upon request directed to the corresponding authors. All biological materials used in this manuscript will be made available upon request to the corresponding authors. Distribution of human patient-derived GSCs may be distributed following completion of a material transfer agreement (MTA) with the appropriate institutions if allowed.

## Code availability

All computational algorithms utilized in the manuscript have been referenced in the corresponding figure legend and described in the methods section. Additional details can be made available upon request.
