# Supplementary material for: Three-dimensional bioprinted glioblastoma microenvironments model cellular dependencies and immune interactions
Source: Cell Res. 2020 Jun 4;30(10):833–53. doi: 10.1038/s41422-020-0338-1 (PMC7608409; doi:10.1038/s41422-020-0338-1)
Supplement: Supplementary file 1 — Supplementary Information [file 41422_2020_338_MOESM1_ESM.pdf]

Figure S1

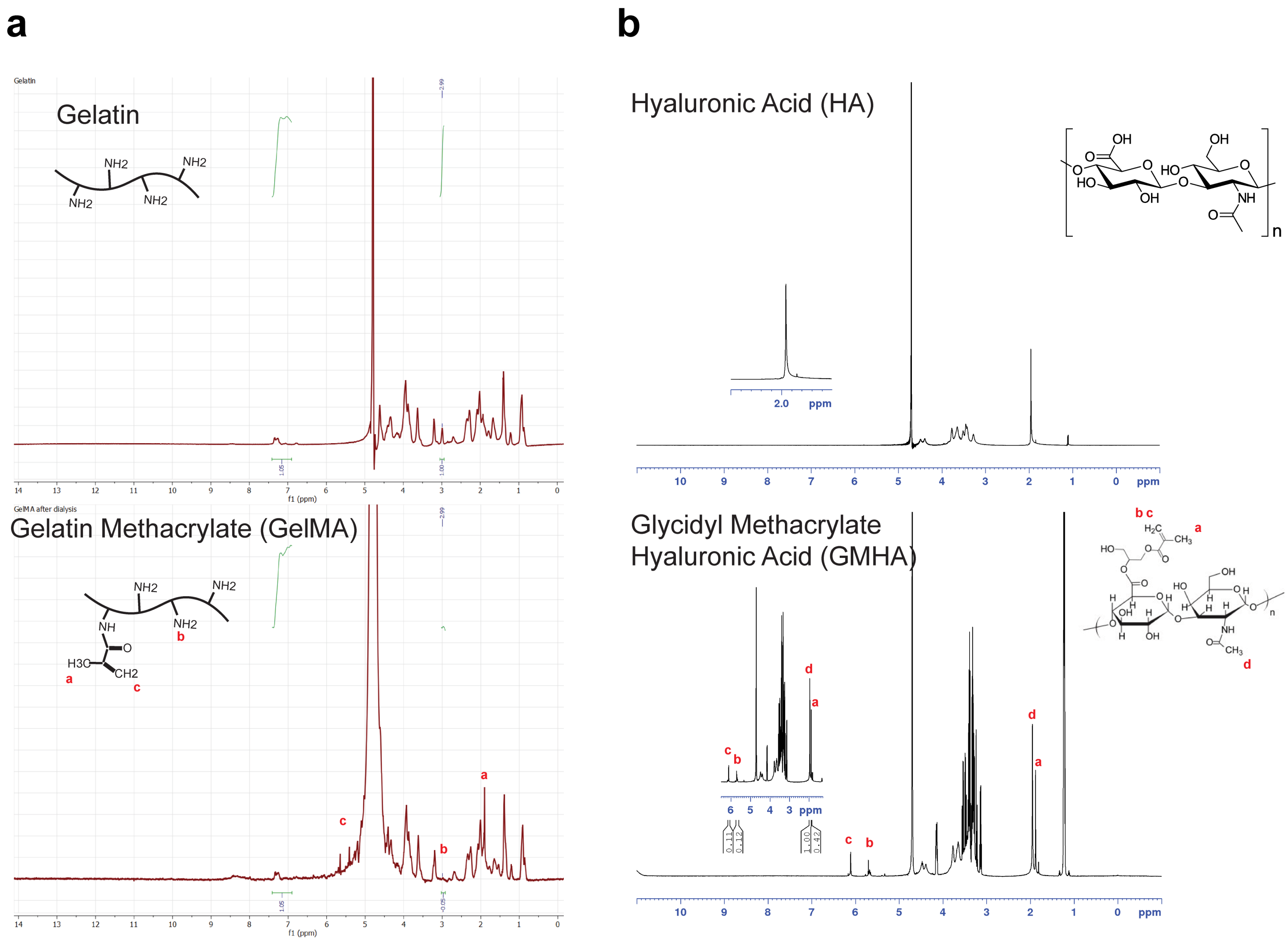
a
b
Gelatin
Gelatin
NH2
GelMA after dialysis
Gelatin Methacrylate (GelMA)
NH
O
H3C
CH2
a
b
c
f1 (ppm)
Hyaluronic Acid (HA)
2.0
ppm
Glycidyl Methacrylate
Hyaluronic Acid (GMHA)
d
ppm

Figure S1: ^{1}H NMR spectra of gelatin, gelatin methacrylate (GelMA), hyaluronic acid (HA), and glycidyl-methacrylate hyaluronic acid (GMHA).

a) The signals of the methyl function of introduced methacrylate, lysine methylene, and acrylic protons are indicated as *a* (1.8ppm), *b* (2.9 ppm), and *c* (5.3 ppm – 5.7 ppm), respectively. Degree of methacrylation is approximated to be around 95% based on the quantification of the lysine methylene signals and the phenylalanine signal (7.1 ppm – 7.4 ppm).

b) The signals of the introduced methacrylate are indicated as *a* (1.8 ppm), *b* (5.6 ppm), and *c* (6.1 ppm) on the spectrum. Degree of methacrylation is approximated to be about 35% based on the intensities of the methacrylate protons compared to intensity of the methyl protons in HA acetamide indicated as *d* (1.9 ppm).

Figure S2

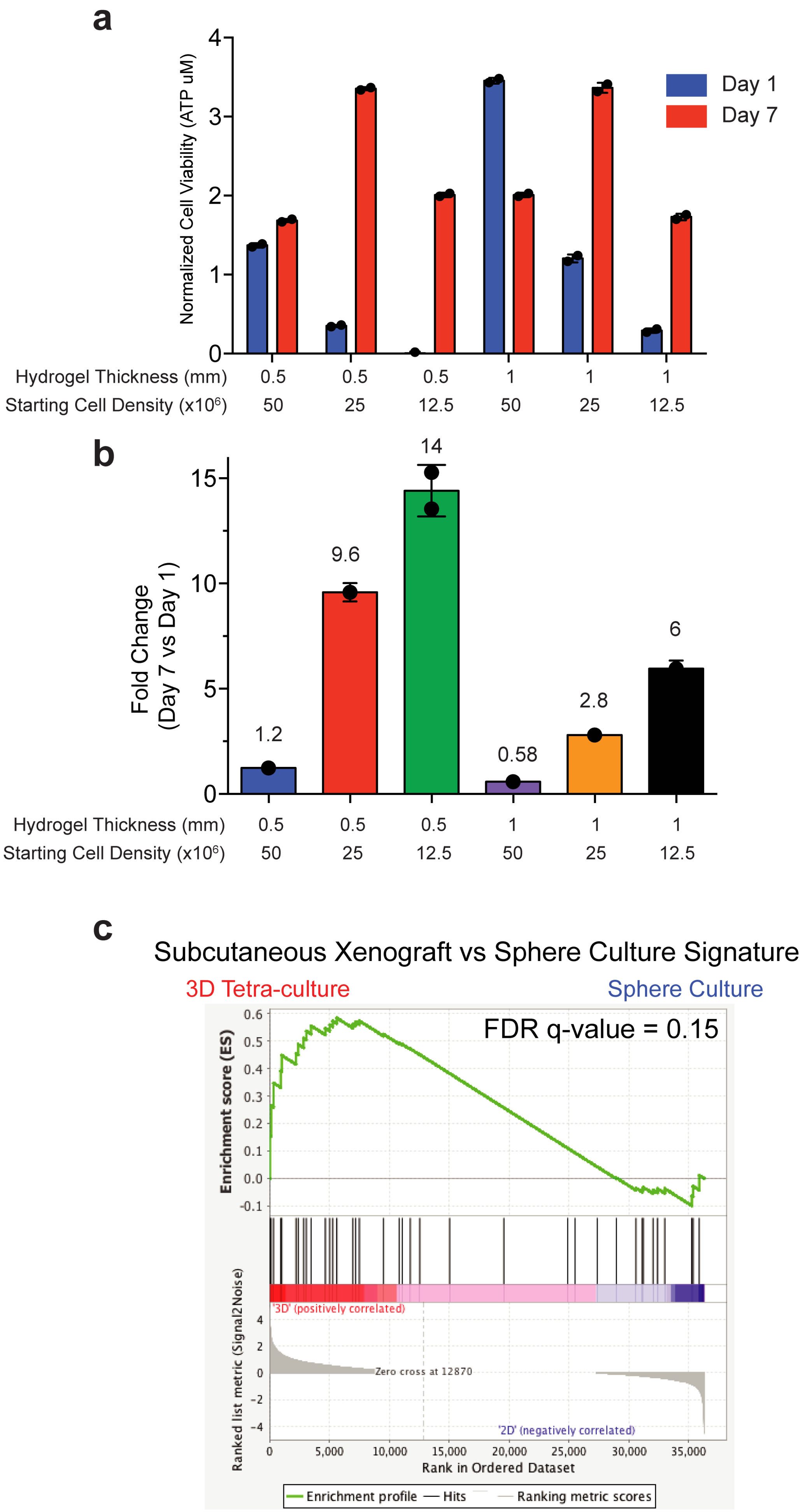

a
Normalized Cell Viability (ATP uM)
Day 1
Day 7
Hydrogel Thickness (mm) 0.5 0.5 0.5 1 1 1
Starting Cell Density (x10^6) 50 25 12.5 50 25 12.5
b
Fold Change (Day 7 vs Day 1)
1.2
9.6
14
0.58
2.8
6
Hydrogel Thickness (mm) 0.5 0.5 0.5 1 1 1
Starting Cell Density (x10^6) 50 25 12.5 50 25 12.5
c
Subcutaneous Xenograft vs Sphere Culture Signature
3D Tetra-culture
Sphere Culture
FDR q-value = 0.15
Enrichment score (ES)
'3D' (positively correlated)
Zero cross at 12870
'2D' (negatively correlated)
Ranked list metric (Signal2Noise)
Rank in Ordered Dataset
Enrichment profile
Hits
Ranking metric scores

Figure S2: Cell growth evaluation in 3D hydrogel and subcutaneous xenograft transcriptional signature.

a) Cell growth in different combinations of hydrogel thickness and starting cell density were evaluated by the amount of ATP present in hydrogel at each time point using CellTiter-Glo luminescent assay. Two replicates were used, bars indicate mean with error bars showing standard deviation.

b) Fold change of cell growth in different combinations of hydrogel thickness and starting cell density. At 500 µm hydrogel thickness, the number of viable cells showed 1.2-fold, 9.6-fold, and 14-fold increase after 7 days in culture, with starting cell density of 50 million/mL, 25 million/mL, and 12.5 million /mL, respectively. At 1 mm hydrogel thickness, the number of viable cells showed 0.58-fold decrease with starting density of 50 million/mL, and 2.8-fold and 6-fold increase with starting cell density of 25 million/mL and 12.5 million/mL, respectively, after 7 days in culture. Two replicates were used, bars indicate mean with error bars showing standard deviation.

c) Gene set enrichment analysis (GSEA) of the glioblastoma subcutaneous xenograft vs cell culture signature when applied to RNA-sequencing data comparing the 3D tetra-culture system with sphere cell culture.

Figure S3

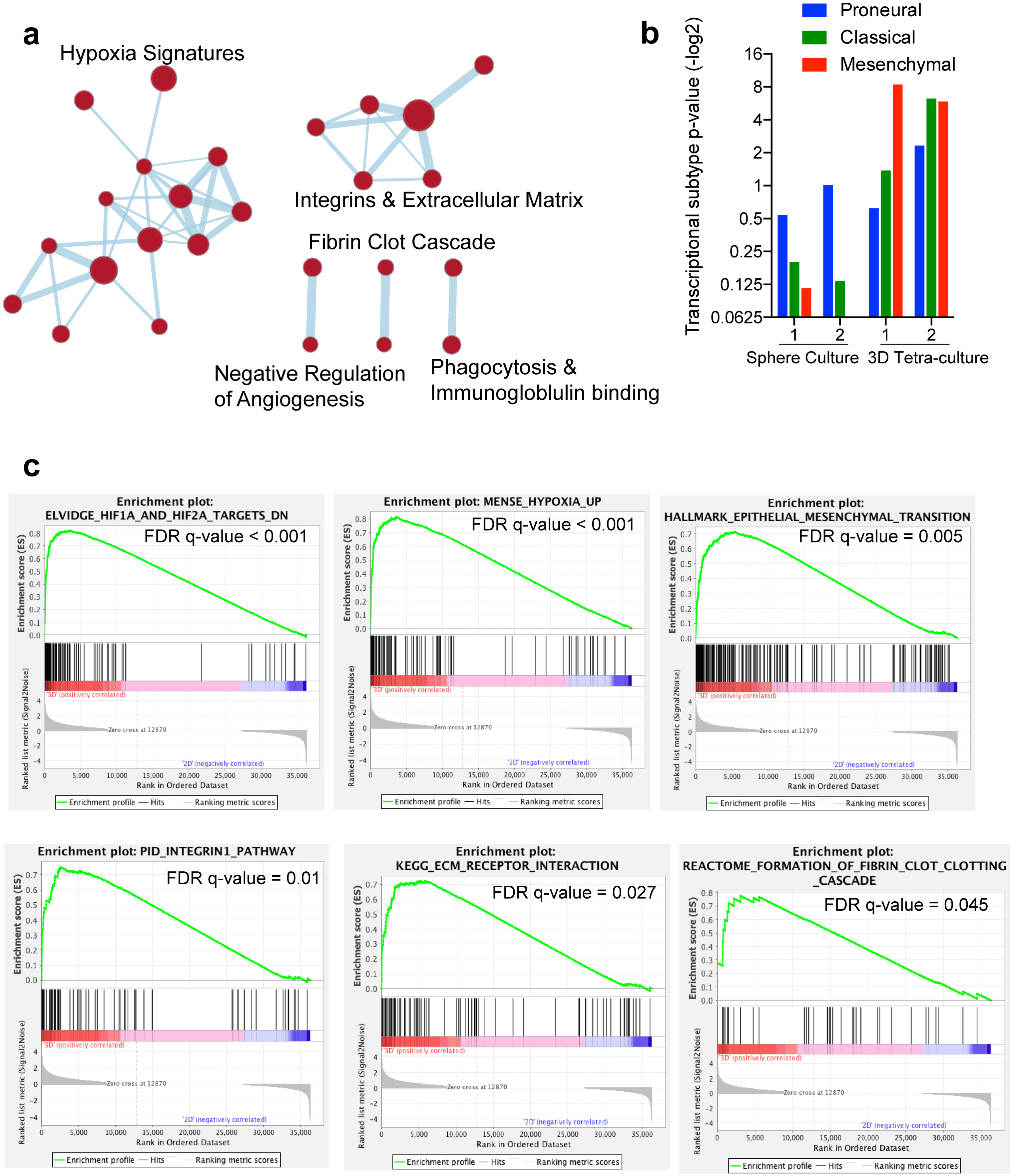
a
Hypoxia Signatures
Integrins & Extracellular Matrix
Fibrin Clot Cascade
Negative Regulation of Angiogenesis
Phagocytosis & Immunogloblulin binding
b
Proneural
Classical
Mesenchymal
Transcriptional subtype p-value (-log2)
16
8
4
2
1
0.5
0.25
0.125
0.0625
1
2
1
2
Sphere Culture
3D Tetra-culture
c
Enrichment plot: ELVIDGE_HIF1A_AND_HIF2A_TARGETS_DN
FDR q-value < 0.001
Enrichment plot: MENSE_HYPOXIA_UP
FDR q-value < 0.001
Enrichment plot: HALLMARK_EPITHELIAL_MESENCHYMAL_TRANSITION
FDR q-value = 0.005
Enrichment plot: PID_INTEGRIN1_PATHWAY
FDR q-value = 0.01
Enrichment plot: KEGG_ECM_RECEPTOR_INTERACTION
FDR q-value = 0.027
Enrichment plot: REACTOME_FORMATION_OF_FIBRIN_CLOT_CLOTTING_CASCADE
FDR q-value = 0.045
Enrichment score (ES)
Ranked list metric (Signal2Noise)
'3D' (positively correlated)
Zero cross at 12870
'2D' (negatively correlated)
Rank in Ordered Dataset
Enrichment profile
Hits
Ranking metric scores

Figure S3: GSCs grown in 3D tetra-culture models upregulate transcriptional signatures of cellular interaction, hypoxia, and cancer stem cells.

a) Pathway gene set enrichment connectivity diagram displaying pathways enriched among gene sets upregulated (red) in GSCs in the 3D tetra-culture system vs. standard sphere culture.

b) Normalized single sample gene set enrichment analysis (ssGSEA) significance values (-log2 of p-value) of glioblastoma transcriptional subtypes for the CW468 GSC when grown in in standard sphere culture vs GSCs in the 3D tetra-culture model. Bar represents the single p-value for each replicate.

c) Gene set enrichment analysis (GSEA) plots showing pathways enriched in GSCs in the 3D tetra-culture system compared with sphere cell culture.

Figure S4

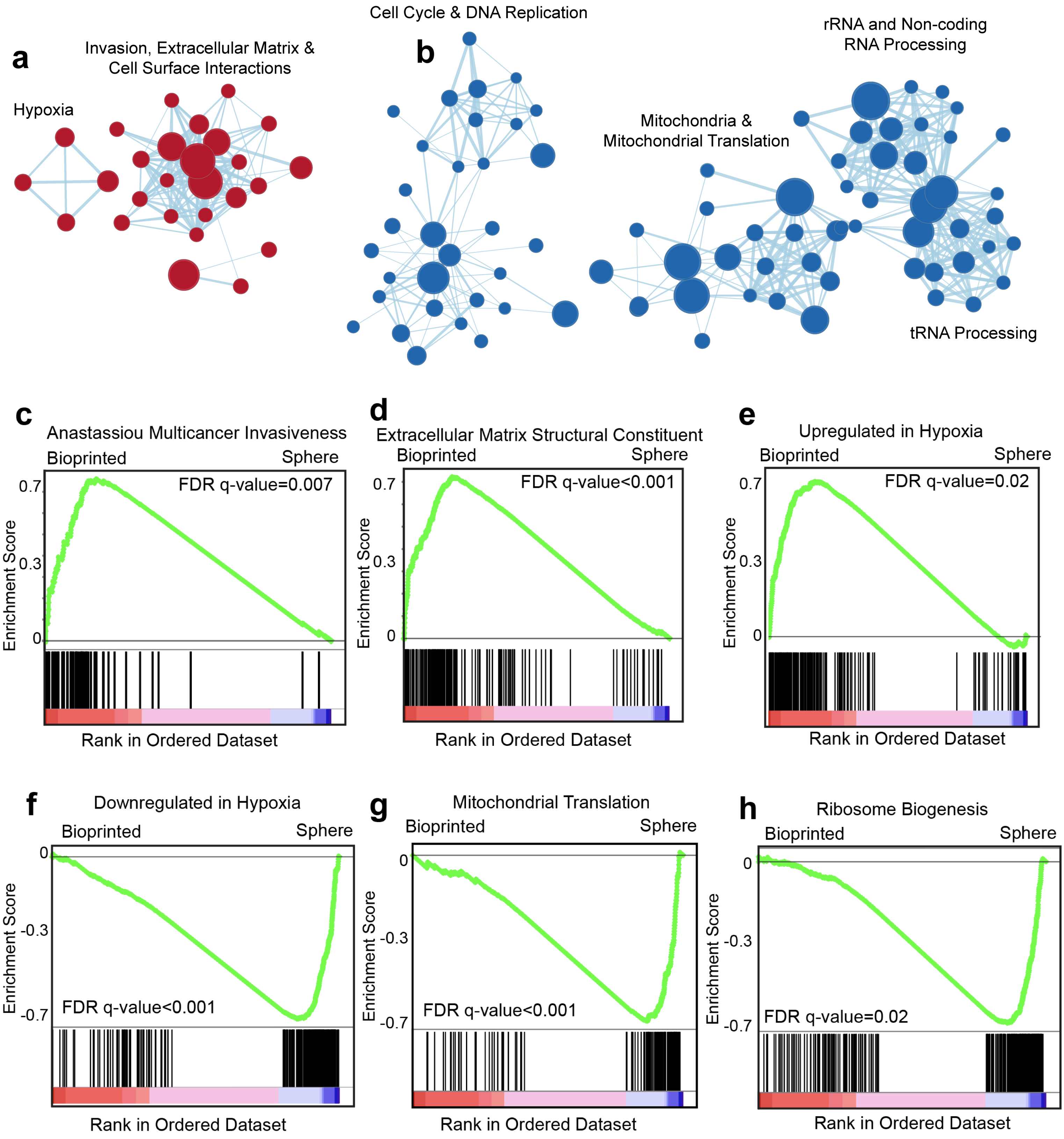
a
Hypoxia
Invasion, Extracellular Matrix & Cell Surface Interactions
b
Cell Cycle & DNA Replication
Mitochondria & Mitochondrial Translation
rRNA and Non-coding RNA Processing
tRNA Processing
c
Anastassiou Multicancer Invasiveness
Bioprinted
Sphere
FDR q-value=0.007
Enrichment Score
0.7
0.3
0
Rank in Ordered Dataset
d
Extracellular Matrix Structural Constituent
Bioprinted
Sphere
FDR q-value<0.001
Enrichment Score
0.7
0.3
0
Rank in Ordered Dataset
e
Upregulated in Hypoxia
Bioprinted
Sphere
FDR q-value=0.02
Enrichment Score
0.7
0.3
0
Rank in Ordered Dataset
f
Downregulated in Hypoxia
Bioprinted
Sphere
FDR q-value<0.001
Enrichment Score
0
-0.3
-0.7
Rank in Ordered Dataset
g
Mitochondrial Translation
Bioprinted
Sphere
FDR q-value<0.001
Enrichment Score
0
-0.3
-0.7
Rank in Ordered Dataset
h
Ribosome Biogenesis
Bioprinted
Sphere
FDR q-value=0.02
Enrichment Score
0
-0.3
-0.7
Rank in Ordered Dataset

Figure S4: 3D bioprinted models display upregulation of hypoxia, invasion, and extracellular matrix pathways.

- **a)** Pathway gene set enrichment connectivity diagram displaying pathways enriched among gene sets upregulated in GSCs in 3D bioprinted models (tri-culture or tetra-cultures) vs. standard sphere culture.
- **b)** Pathway gene set enrichment connectivity diagram displaying pathways enriched among gene sets downregulated in GSCs in 3D bioprinted models (tri-culture or tetra-cultures) vs. standard sphere culture.
- **c)** Gene set enrichment analysis (GSEA) of the Anastassiou multicancer invasiveness pathway between GSCs from 3D bioprinted models vs sphere culture. FDR q-value = 0.007.
- **d)** Gene set enrichment analysis (GSEA) of the extracellular matrix structural constituent pathway between GSCs from 3D bioprinted models vs sphere culture. FDR q-value < 0.001.
- **e)** Gene set enrichment analysis (GSEA) of an "upregulated in hypoxia" signature between GSCs from 3D bioprinted models vs sphere culture. FDR q-value = 0.02.
- **f)** Gene set enrichment analysis (GSEA) of an "downregulated in hypoxia" signature between GSCs from 3D bioprinted models vs sphere culture. FDR q-value < 0.001.
- **g)** Gene set enrichment analysis (GSEA) of a mitochondrial translation signature between GSCs from 3D bioprinted models vs sphere culture. FDR q-value < 0.001.
- **h)** Gene set enrichment analysis (GSEA) of a ribosome biogenesis signature between GSCs from 3D bioprinted models vs sphere culture. FDR q-value = 0.02.

Figure S5

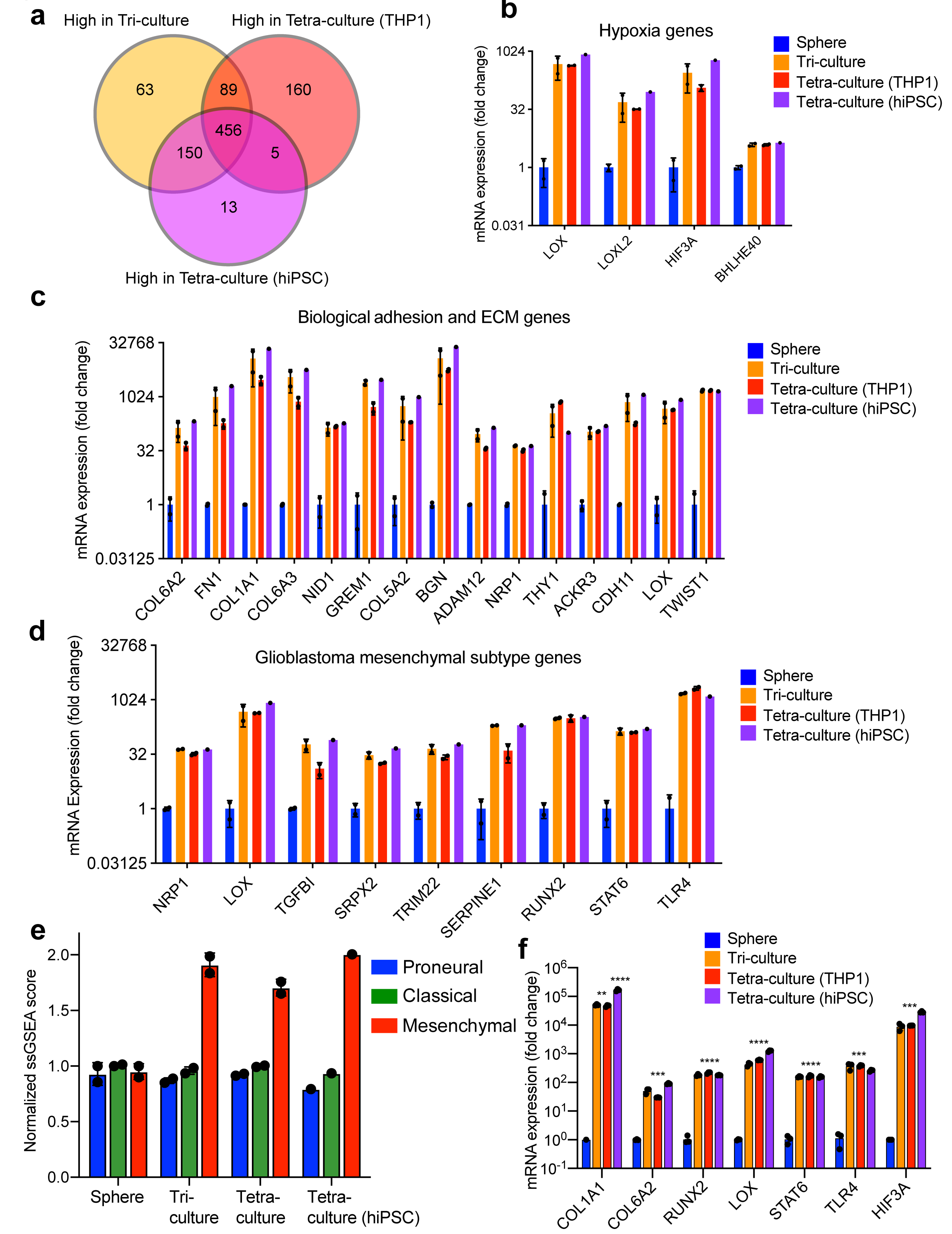
a
High in Tri-culture
High in Tetra-culture (THP1)
High in Tetra-culture (hiPSC)
63
89
160
456
150
5
13
b
Hypoxia genes
Sphere
Tri-culture
Tetra-culture (THP1)
Tetra-culture (hiPSC)
mRNA expression (fold change)
1024
32
1
0.031
LOX
LOXL2
HIF3A
BHLHE40
c
Biological adhesion and ECM genes
32768
1024
32
1
0.03125
COL6A2
FN1
COL1A1
COL6A3
NID1
GREM1
COL5A2
BGN
ADAM12
NRP1
THY1
ACKR3
CDH11
LOX
TWIST1
d
Glioblastoma mesenchymal subtype genes
mRNA Expression (fold change)
NRP1
LOX
TGFBI
SRPX2
TRIM22
SERPINE1
RUNX2
STAT6
TLR4
e
Normalized ssGSEA score
2.0
1.5
1.0
0.5
0.0
Proneural
Classical
Mesenchymal
Sphere
Tri-culture
Tetra-culture
Tetra-culture (hiPSC)
f
10^6
10^5
10^4
10^3
10^2
10^1
10^0
10^{-1}
**
****
***
****
****
****
***
***
COL1A1
COL6A2
RUNX2
LOX
STAT6
TLR4
HIF3A

Figure S5: Identification of broad concordance between bioprinting models containing different macrophage components.

- **a)** Overlap of genes highly expressed in GSCs derived from three bioprinting conditions vs. GSCs in sphere culture (Log2 Fold Change > 0.5, adjusted p-value < 1e-10).
- **b)** mRNA expression (TPM) normalized to sphere culture values of several hypoxia response genes in sphere culture and in three bioprinting models. Bars are centered at the mean and error bars indicate standard deviation. For all displayed genes, the mRNA expression Log2 fold change was greater than 0.5 and the adjusted p-value was less than 1e-10.
- **c)** mRNA expression (TPM) normalized to sphere culture values of biological adhesion and extracellular matrix genes in sphere culture and in three bioprinting models. Bars are centered at the mean and error bars indicate standard deviation. For all displayed genes, the mRNA expression Log2 fold change was greater than 0.5 and the adjusted p-value was less than 1e-10.
- **d)** mRNA expression (TPM) normalized to sphere culture values of glioblastoma mesenchymal subtype genes in sphere culture and in three bioprinting models. Bars are centered at the mean and error bars indicate standard deviation. For all displayed genes, the mRNA expression Log2 fold change was greater than 0.5 and the adjusted p-value was less than 1e-10.
- **e)** Normalized single sample GSEA (ssGSEA) score for the proneural, classical, and mesenchymal glioblastoma subtype transcriptional signatures in sphere cultured cells vs bioprinted models.

f) mRNA expression as assessed by qPCR normalized to sphere culture values of several hypoxia response, extracellular matrix, and glioblastoma mesenchymal subtype genes in sphere cultured cells vs bioprinted models. **, $p < 0.01$; ***, $p < 0.001$; ****, $p < 0.0001$. Bars are centered at the mean and error bars indicate standard deviation. Ordinary one-way ANOVA with Dunnett multiple comparisons test was used for statistical analysis.

Figure S6

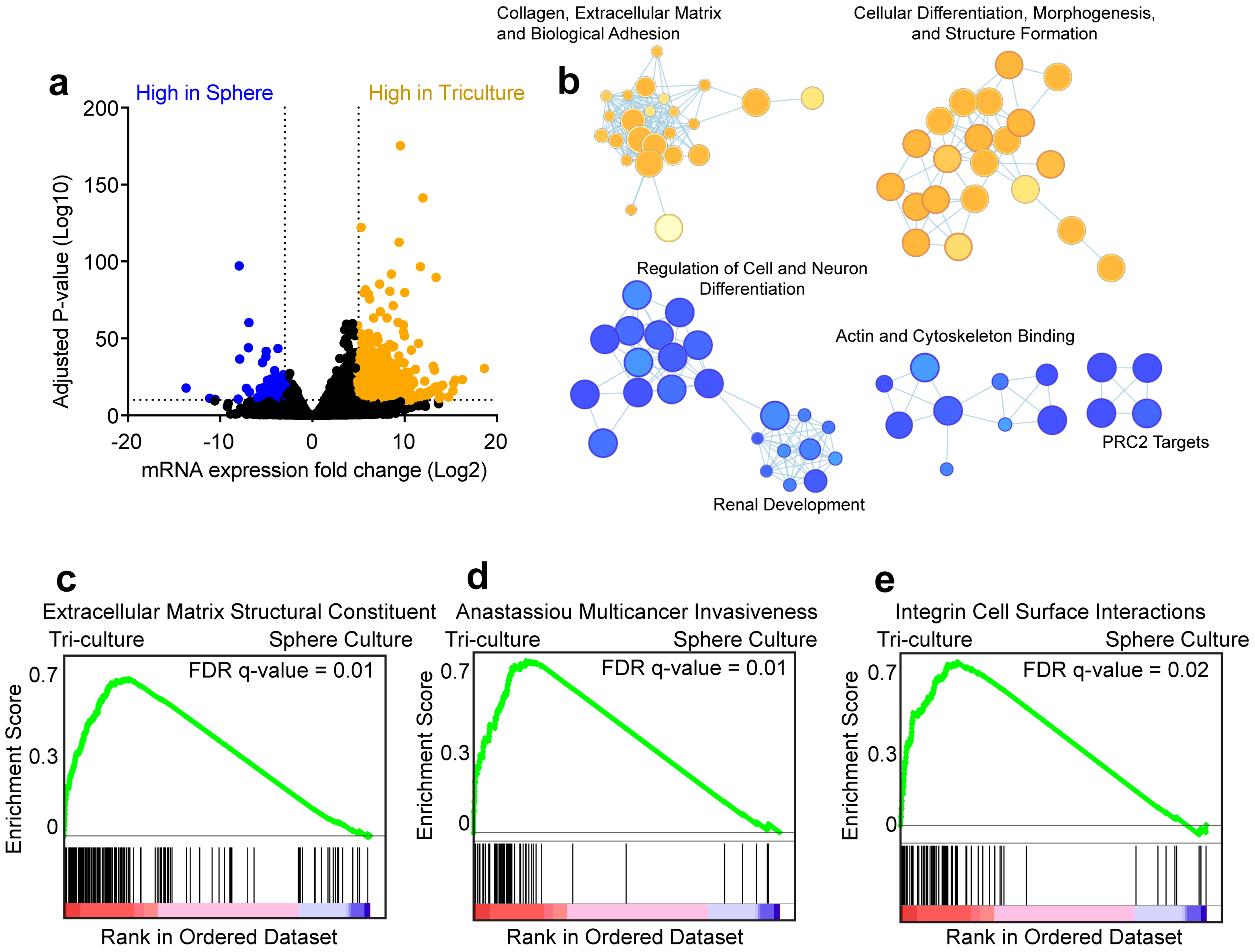

a
High in Sphere
High in Triculture
Adjusted P-value (Log10)
200
150
100
50
0
-20
-10
0
10
20
mRNA expression fold change (Log2)
b
Collagen, Extracellular Matrix and Biological Adhesion
Cellular Differentiation, Morphogenesis, and Structure Formation
Regulation of Cell and Neuron Differentiation
Actin and Cytoskeleton Binding
PRC2 Targets
Renal Development
c
Extracellular Matrix Structural Constituent
Tri-culture
Sphere Culture
FDR q-value = 0.01
Enrichment Score
0.7
0.3
0
Rank in Ordered Dataset
d
Anastassiou Multicancer Invasiveness
Tri-culture
Sphere Culture
FDR q-value = 0.01
Enrichment Score
0.7
0.3
0
Rank in Ordered Dataset
e
Integrin Cell Surface Interactions
Tri-culture
Sphere Culture
FDR q-value = 0.02
Enrichment Score
0.7
0.3
0
Rank in Ordered Dataset

Figure S6: 3D tri-culture models upregulate cell surface interaction, extracellular matrix, and biological adhesion signatures compared to sphere culture.

a) Volcano plot of transcriptional landscape profiled by RNA-sequencing comparing the CW468 GSC grown in standard sphere culture vs GSCs in the 3D tri-culture model. The x-axis depicts the log transformed fold change, while the y-axis shows the log transformed p-value adjusted for multiple test correction. n=2 technical replicates per condition.

b) Pathway gene set enrichment connectivity diagram displaying pathways enriched among gene sets (orange) upregulated or (blue) downregulated in GSCs in 3D tri-culture models) vs. standard sphere culture.

c) Gene set enrichment analysis (GSEA) of the extracellular matrix structural constituent pathway between GSCs from 3D tri-culture models vs sphere culture. FDR q-value = 0.01.

d) Gene set enrichment analysis (GSEA) of the Anastassiou multiculture invasiveness signature between GSCs from 3D tri-culture models vs sphere culture. FDR q-value = 0.01.

e) Gene set enrichment analysis (GSEA) of the integrin cell surface interaction signature between GSCs from 3D tri-culture models vs sphere culture. FDR q-value = 0.02.

Figure S7

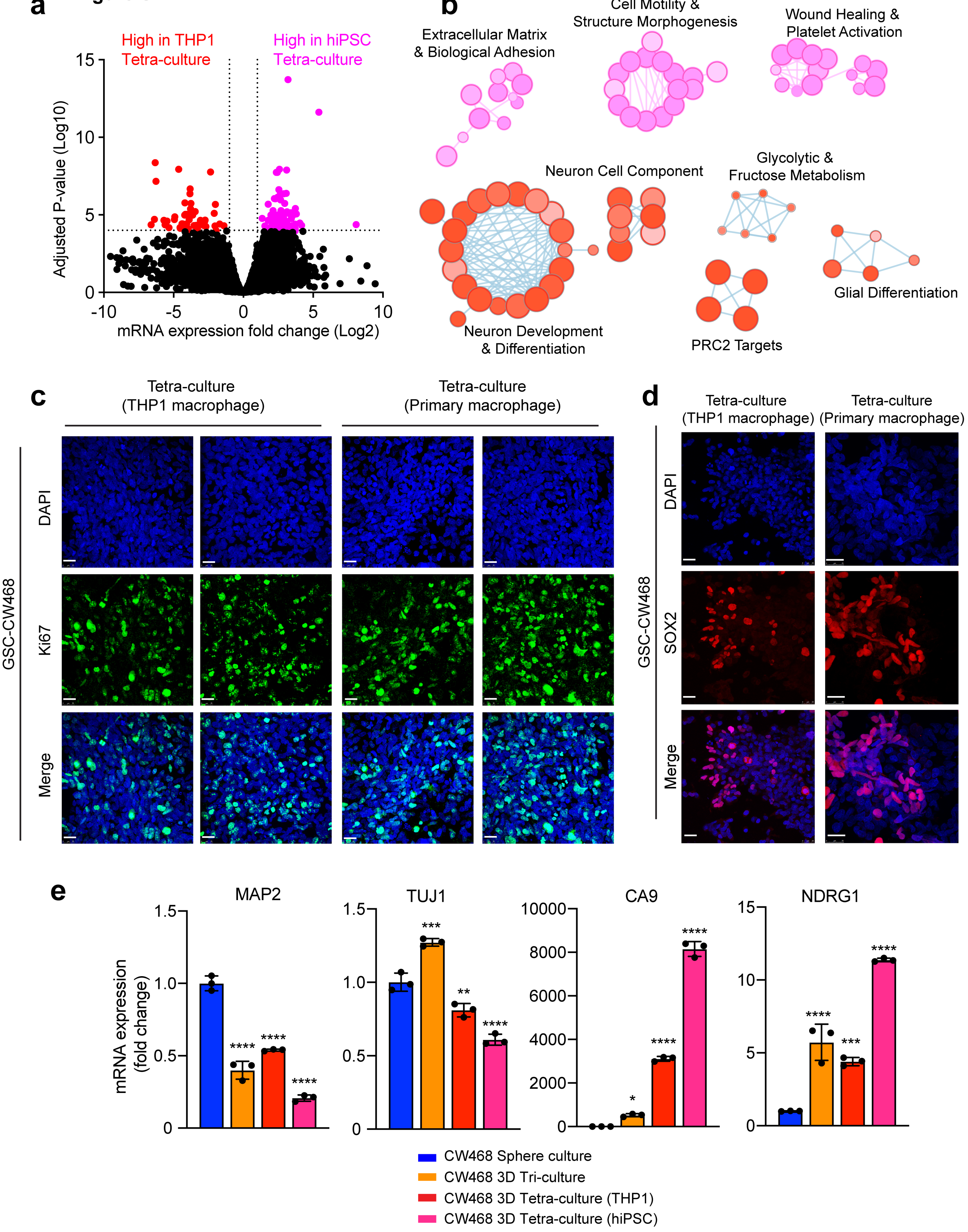
a
High in THP1 Tetra-culture
High in hiPSC Tetra-culture
Adjusted P-value (Log10)
mRNA expression fold change (Log2)
b
Extracellular Matrix & Biological Adhesion
Cell Motility & Structure Morphogenesis
Wound Healing & Platelet Activation
Neuron Cell Component
Glycolytic & Fructose Metabolism
Glial Differentiation
Neuron Development & Differentiation
PRC2 Targets
c
Tetra-culture (THP1 macrophage)
Tetra-culture (Primary macrophage)
GSC-CW468
DAPI
Ki67
Merge
d
Tetra-culture (THP1 macrophage)
Tetra-culture (Primary macrophage)
GSC-CW468
DAPI
SOX2
Merge
e
MAP2
TUJ1
CA9
NDRG1
mRNA expression (fold change)
CW468 Sphere culture
CW468 3D Tri-culture
CW468 3D Tetra-culture (THP1)
CW468 3D Tetra-culture (hiPSC)

Figure S7: Comparison of 3D tetra-cultures with macrophages derived from THP1 cells or hiPSCs.

- **a)** Volcano plot of transcriptional landscape profiled by RNA-sequencing comparing the CW468 GSC grown in tetra-cultures with THP1 derived macrophages vs tetra-cultures with human induced pluripotent stem cell (hiPSC) derived macrophages. The x-axis depicts the log transformed fold change, while the y-axis shows the log transformed p-value adjusted for multiple test correction.
- **b)** Pathway gene set enrichment connectivity diagram displaying pathways enriched among gene sets (purple) upregulated or (red) downregulated in GSCs from tetra-cultures with hiPSC-derived macrophages vs tetra-cultures with THP1-derived macrophages.
- **c)** Immunofluorescence imaging of Ki67 in CW468 GSCs in tetra-cultures containing THP1-derived macrophages or primary human macrophages. Scale bar, 25µm.
- **d)** Immunofluorescence imaging of SOX2 in CW468 GSCs in tetra-cultures containing THP1-derived macrophages or primary human macrophages. Scale bar, 25µm.
- **e)** qPCR analysis of mRNA expression of MAP2, TUJ1, CA9, and NDRG1 in GSCs derived from sphere culture, tri-cultures, tetra-cultures with THP1-derived macrophages, and tetra-cultures with hiPSC-derived macrophages. *, $p < 0.05$; ***, $p < 0.001$; ****, $p < 0.0001$. Ordinary one-way ANOVA with Dunnett multiple comparisons testing was used for statistical analysis.

Figure S8

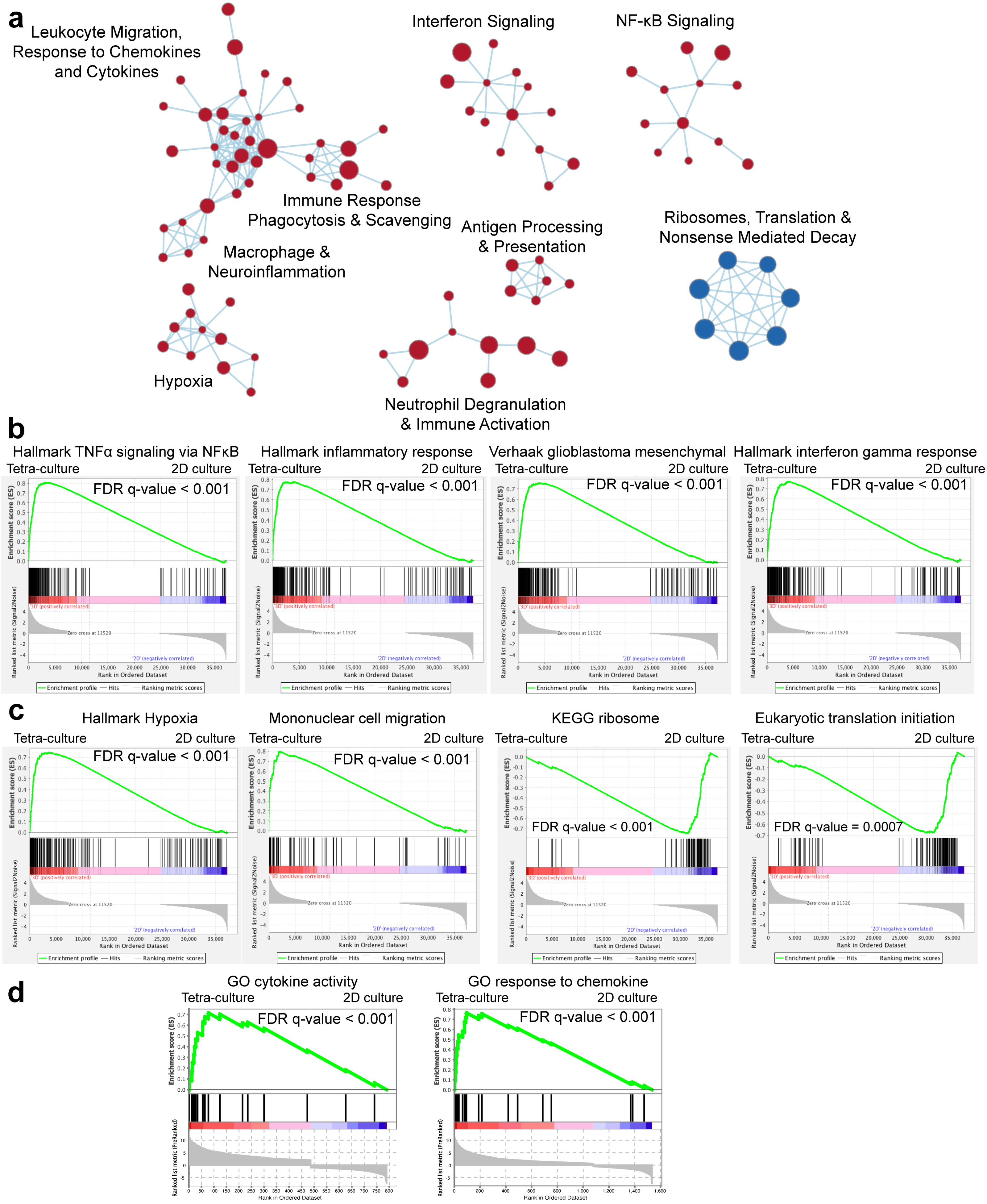
a
Leukocyte Migration, Response to Chemokines and Cytokines
Interferon Signaling
NF-κB Signaling
Immune Response Phagocytosis & Scavenging
Antigen Processing & Presentation
Ribosomes, Translation & Nonsense Mediated Decay
Macrophage & Neuroinflammation
Hypoxia
Neutrophil Degranulation & Immune Activation
b
Hallmark TNFα signaling via NFκB
Hallmark inflammatory response
Verhaak glioblastoma mesenchymal
Hallmark interferon gamma response
Tetra-culture
2D culture
FDR q-value < 0.001
c
Hallmark Hypoxia
Mononuclear cell migration
KEGG ribosome
Eukaryotic translation initiation
FDR q-value < 0.001
FDR q-value = 0.0007
d
GO cytokine activity
GO response to chemokine
Enrichment score (ES)
Ranked list metric (Signal2Noise)
Ranked list metric (PreRanked)
Rank in Ordered Dataset
'3D' (positively correlated)
'2D' (negatively correlated)
Zero cross at 11520
Enrichment profile
Hits
Ranking metric scores

Figure S8: Macrophages grown in 3D tetra-culture models upregulate immune activation signatures, increase M2 polarization, and promote GSC invasion.

- **a)** Pathway gene set enrichment connectivity diagram displaying pathways enriched among gene sets upregulated (red) and downregulated (blue) in macrophages in the 3D tetra-culture system vs. standard sphere culture.
- **b)** Gene set enrichment analysis (GSEA) plots showing pathways enriched in macrophages in the 3D tetra-culture system compared with sphere cell culture.
- **c)** Gene set enrichment analysis (GSEA) plots showing pathways enriched (or depleted) in macrophages in the 3D tetra-culture system compared with sphere cell culture.
- **d)** Gene set enrichment analysis (GSEA) plots showing pathways enriched in macrophages in the 3D tetra-culture system compared with sphere cell culture.

Figure S9

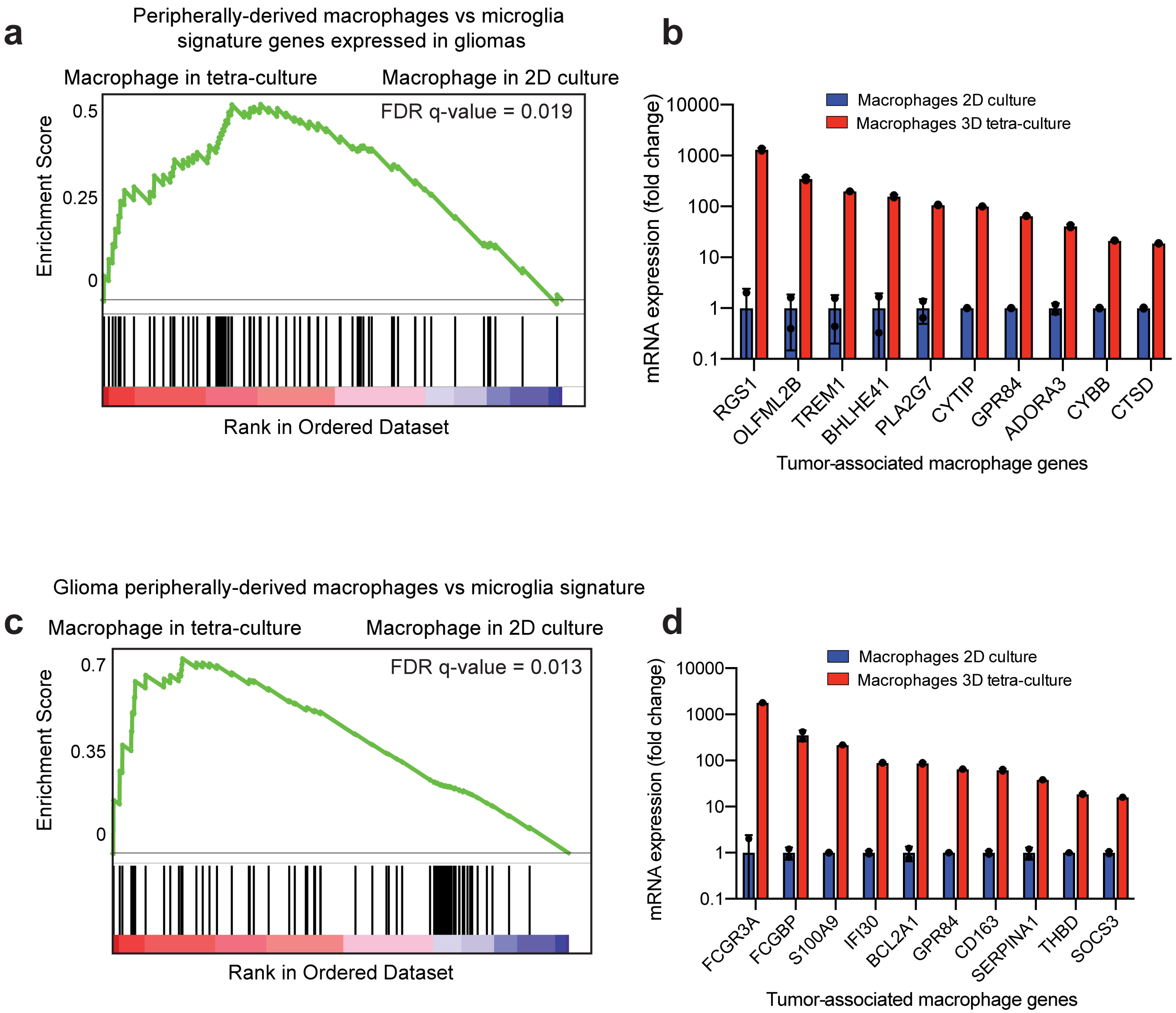
a
Peripherally-derived macrophages vs microglia
signature genes expressed in gliomas
Macrophage in tetra-culture
Macrophage in 2D culture
FDR q-value = 0.019
Enrichment Score
0.5
0.25
0
Rank in Ordered Dataset
b
Macrophages 2D culture
Macrophages 3D tetra-culture
mRNA expression (fold change)
10000
1000
100
10
1
0.1
RGS1
OLFML2B
TREM1
BHLHE41
PLA2G7
CYTIP
GPR84
ADORA3
CYBB
CTSD
Tumor-associated macrophage genes
Glioma peripherally-derived macrophages vs microglia signature
c
Macrophage in tetra-culture
Macrophage in 2D culture
FDR q-value = 0.013
Enrichment Score
0.7
0.35
0
Rank in Ordered Dataset
d
Macrophages 2D culture
Macrophages 3D tetra-culture
mRNA expression (fold change)
10000
1000
100
10
1
0.1
FCGR3A
FCGBP
S100A9
IFI30
BCL2A1
GPR84
CD163
SERPINA1
THBD
SOCS3
Tumor-associated macrophage genes

Figure S9: Expression of glioma macrophage signatures in 3D tetra-culture-derived macrophages.

a) Gene set enrichment analysis of "glioma peripherally-derived macrophages vs microglia" signature genes derived from Muller et al.

b) mRNA expression (TPM) fold change of selected genes from the signature defined in (a) in macrophages derived from 2D culture or the 3D tetra-culture model.

c) Gene set enrichment analysis of "glioma peripherally derived macrophages vs microglia" signature genes derived from Venteicher et al.

d) mRNA expression (TPM) fold change of selected genes from the signature defined in (c) in macrophages derived from 2D culture or the 3D tetra-culture model.

Figure S10

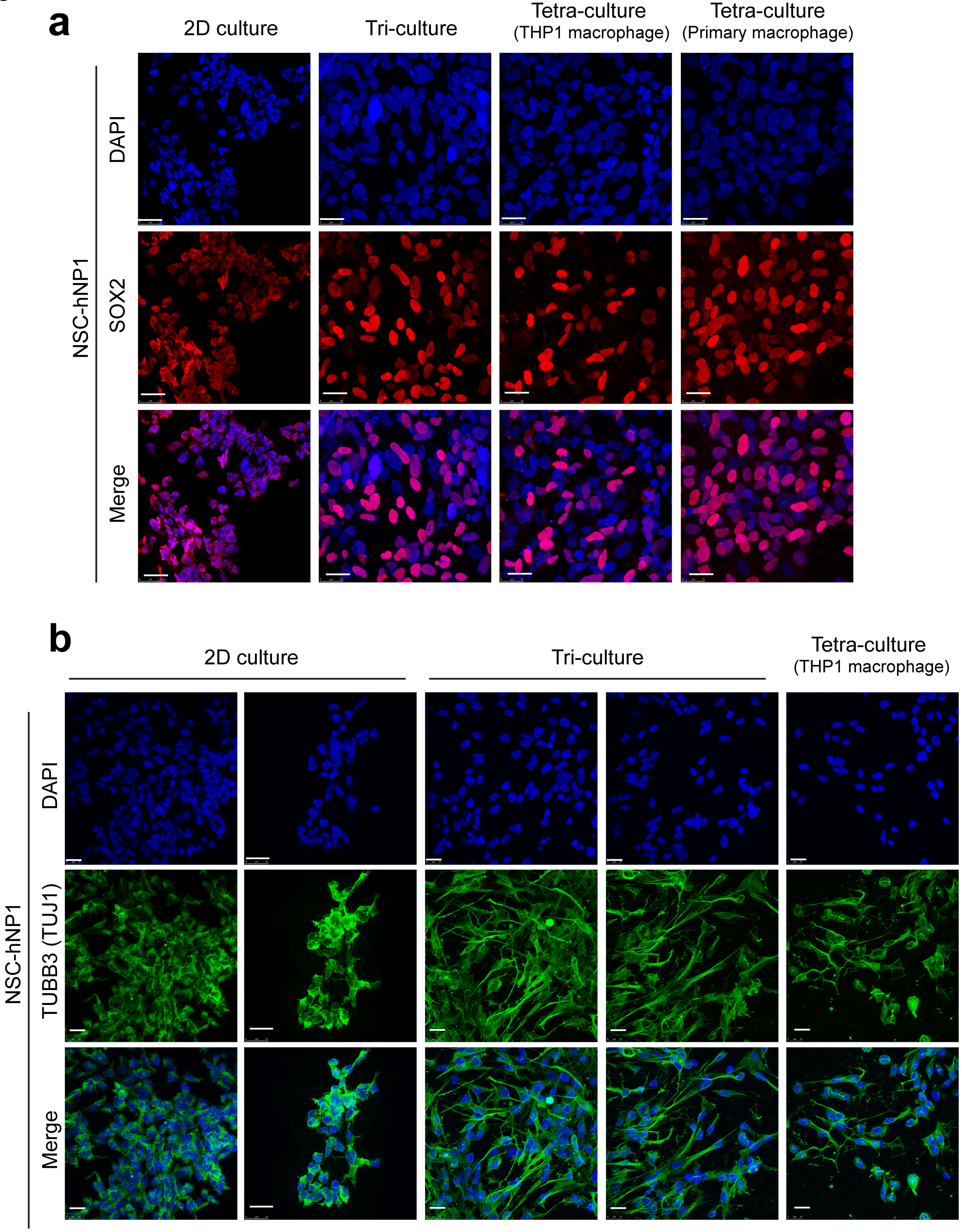

a
2D culture
Tri-culture
Tetra-culture
(THP1 macrophage)
Tetra-culture
(Primary macrophage)
NSC-hNP1
DAPI
SOX2
Merge
b
2D culture
Tri-culture
Tetra-culture
(THP1 macrophage)
NSC-hNP1
DAPI
TUBB3 (TUJ1)
Merge

Figure S10: NPCs retain stem markers in bioprinted culture but adopt some neuron-like morphologies.

a) Immunofluorescence analysis of SOX2 in NPCs (hNP1) in 2D culture, tri-culture, tetra-culture containing THP1-derived macrophages, and tetra-culture containing primary human macrophages. Scale bar, 25µm.

b) Immunofluorescence analysis of TUBB3 (TUJ1) in NPCs (hNP1) in 2D culture, tri-culture, tetra-culture containing THP1-derived macrophages, and tetra-culture containing primary human macrophages. Scale bar, 25µm.

Figure S11

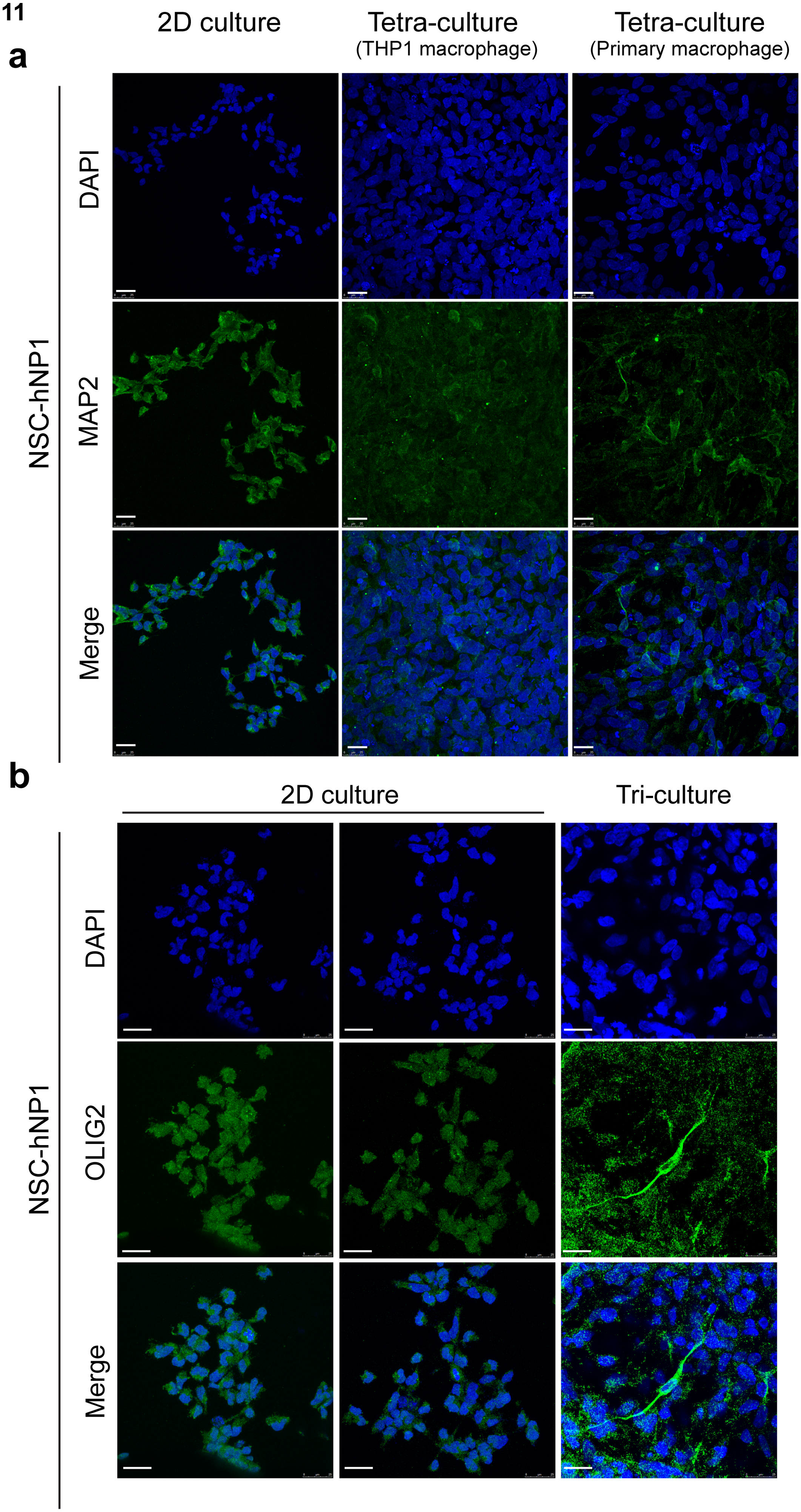
a
2D culture
Tetra-culture
(THP1 macrophage)
Tetra-culture
(Primary macrophage)
NSC-hNP1
DAPI
MAP2
Merge
b
2D culture
Tri-culture
NSC-hNP1
DAPI
OLIG2
Merge

Figure S11: Rare NPCs may differentiate down an oligodendrocytic lineage.

a) Immunofluorescence analysis of MAP2 in NPCs (hNP1) in 2D culture, tetra-culture containing THP1-derived macrophages, and tetra-culture containing primary human macrophages. Scale bar, 25µm.

b) Immunofluorescence analysis of OLIG2 in NPCs (hNP1) in 2D culture and tri-culture. Scale bar, 25µm.

Figure S12

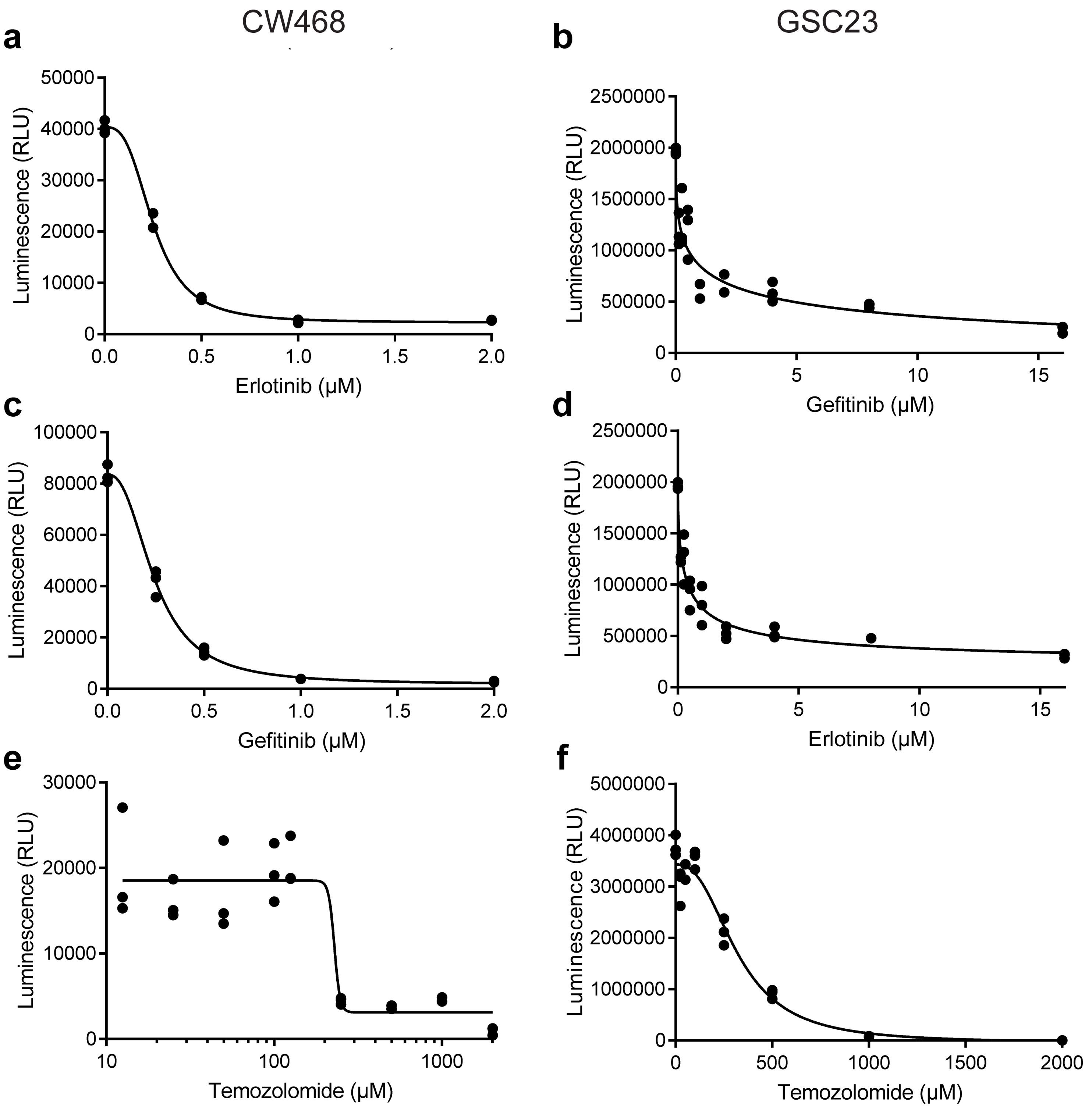
CW468
GSC23
a
b
c
d
e
f
Luminescence (RLU)
Erlotinib (μM)
Gefitinib (μM)
Temozolomide (μM)
50000
40000
30000
20000
10000
0
0.0
0.5
1.0
1.5
2.0
2500000
2000000
1500000
1000000
500000
5
10
15
100000
80000
60000
40000
20000
30000
100
1000
5000000
4000000
3000000
500
1500
2000

Figure S12: Drug response evaluation for sphere suspension culture.

- **a)** Luciferase labeled CW468 were seeded in 96-well plate with erlotinib. Drug responses were evaluated after 72 hours. IC_{50} was 0.2538 µM.
- **b)** Luciferase labeled GSC23 were seeded in 96-well plate with gefitinib. Drug responses were evaluated after 72 hours. IC_{50} was 0.9284 µM.
- **c)** Luciferase labeled CW468 were seeded in 96-well plate with gefitinib. Drug responses were evaluated after 72 hours. IC_{50} was 0.2947 µM.
- **d)** Luciferase labeled GSC23 were seeded in 96-well plate with erlotinib. Drug responses were evaluated after 72 hours. IC_{50} was 0.3177 µM.
- **e)** Luciferase labeled CW468 were seeded in 96-well plate with temozolomide. Drug responses were evaluated after 144 hours. IC_{50} was 228.3 µM.
- **f)** Luciferase labeled GSC23 were seeded in 96-well plate with temozolomide. Drug responses were evaluated after 144 hours. IC_{50} was 322.5 µM.

Figure S13

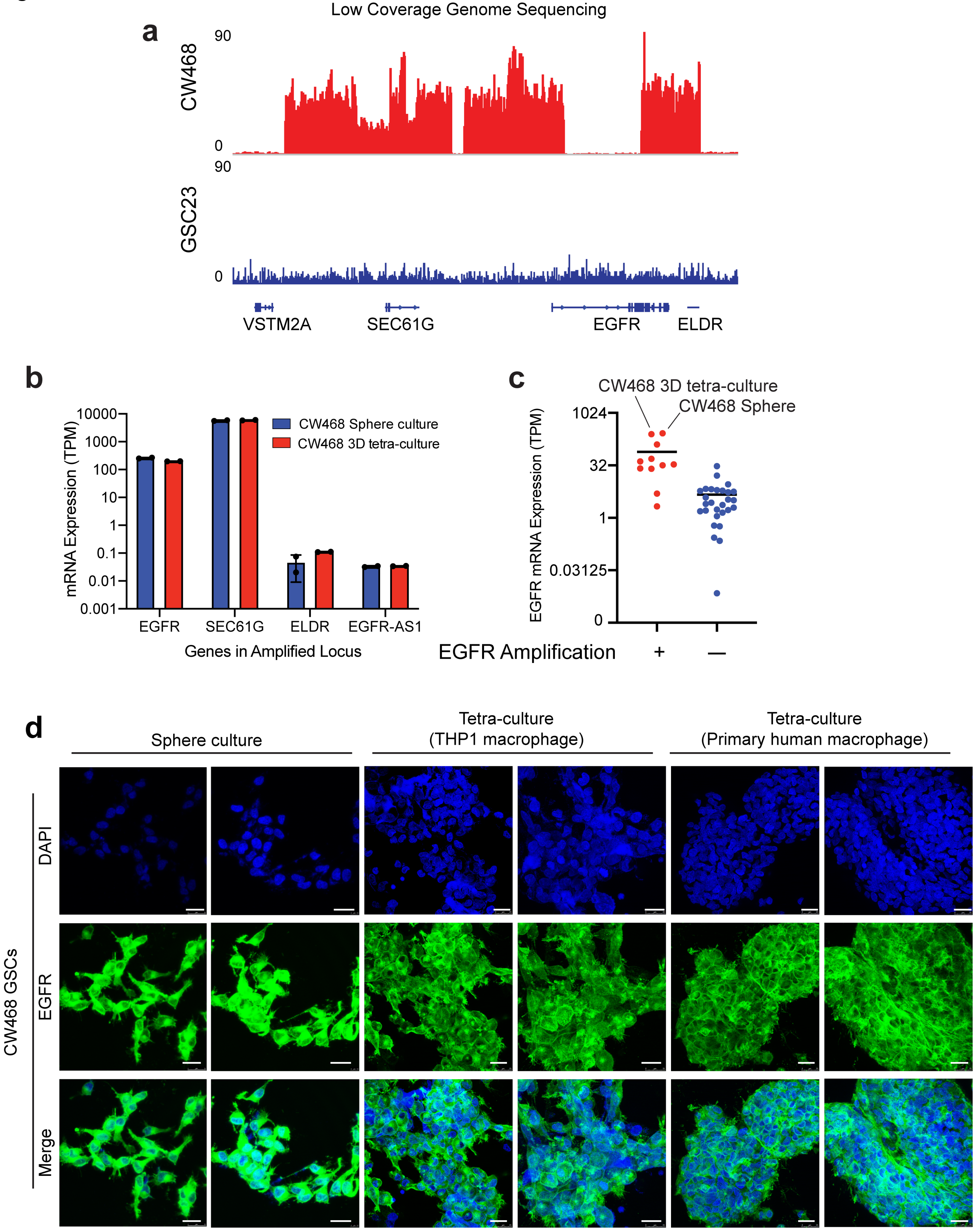

a
Low Coverage Genome Sequencing
CW468
90
0
GSC23
90
0
VSTM2A
SEC61G
EGFR
ELDR
b
mRNA Expression (TPM)
10000
1000
100
10
1
0.1
0.01
0.001
CW468 Sphere culture
CW468 3D tetra-culture
EGFR
SEC61G
ELDR
EGFR-AS1
Genes in Amplified Locus
c
CW468 3D tetra-culture
CW468 Sphere
EGFR mRNA Expression (TPM)
1024
32
1
0.03125
0
EGFR Amplification
+
—
d
Sphere culture
Tetra-culture (THP1 macrophage)
Tetra-culture (Primary human macrophage)
CW468 GSCs
DAPI
EGFR
Merge

Figure S13: EGFR amplification and expression are maintained in GSCs in tetra-cultures

- **a)** Low coverage whole genome sequencing data showing the amplification status of EGFR in the CW468 and GSC23 cells in sphere culture.
- **b)** mRNA expression (TPM, transcripts per million) for genes in the genomic region of EGFR amplification in CW468 cells grown in sphere culture and in the tetra-culture model.
- **c)** EGFR mRNA expression (TPM, transcripts per million) in GSCs based on the EGFR amplification status. GSC RNA-seq data was derived from Mack *et al.*
- **d)** Immunofluorescence of total EGFR expression in GSCs in sphere culture, tetra-cultures containing THP1-derived macrophages, and tetra-cultures containing primary human macrophages. Scale bar, 25µm.

Figure S14

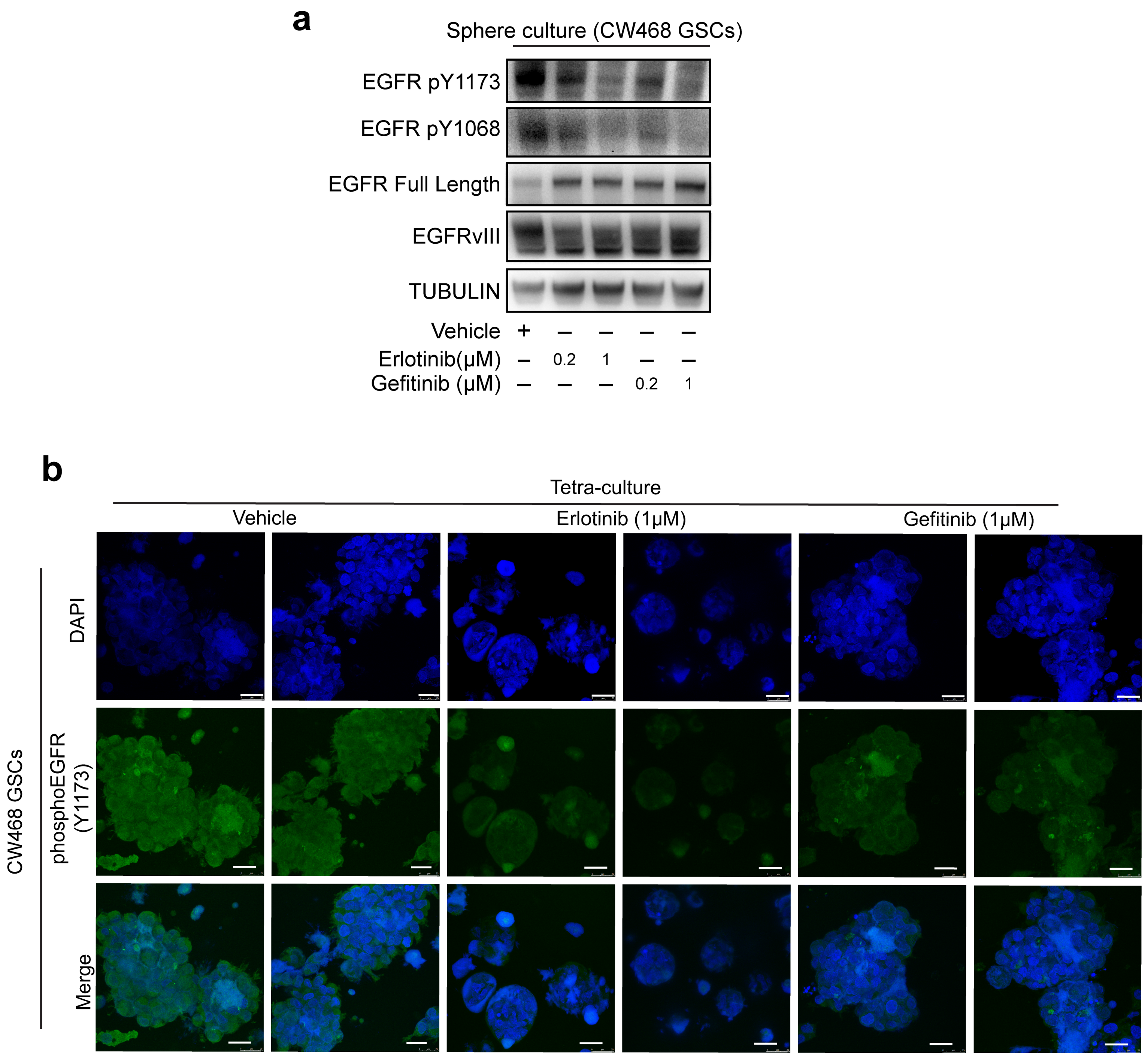
a
Sphere culture (CW468 GSCs)
EGFR pY1173
EGFR pY1068
EGFR Full Length
EGFRvIII
TUBULIN
Vehicle + – – – –
Erlotinib(μM) – 0.2 1 – –
Gefitinib (μM) – – – 0.2 1
b
Tetra-culture
Vehicle
Erlotinib (1μM)
Gefitinib (1μM)
CW468 GSCs
DAPI
phosphoEGFR (Y1173)
Merge

Figure S14: Erlotinib and gefitinib display on-target effects in sphere and tetra-culture models.

a) Western blot showing EGFR phosphorylation (Y1173 and Y1068) as a biomarker of EGFR activation following treatment with erlotinib or gefitinib for 24 hours in sphere culture at the indicated concentrations.

b) Immunofluorescence showing phospho-EGFR (Y1173) expression in GSCs in 3D tetra-cultures following treatment with 1µM of erlotinib or gefitinib for 24 hours. Scale bar, 25µm.

Figure S15

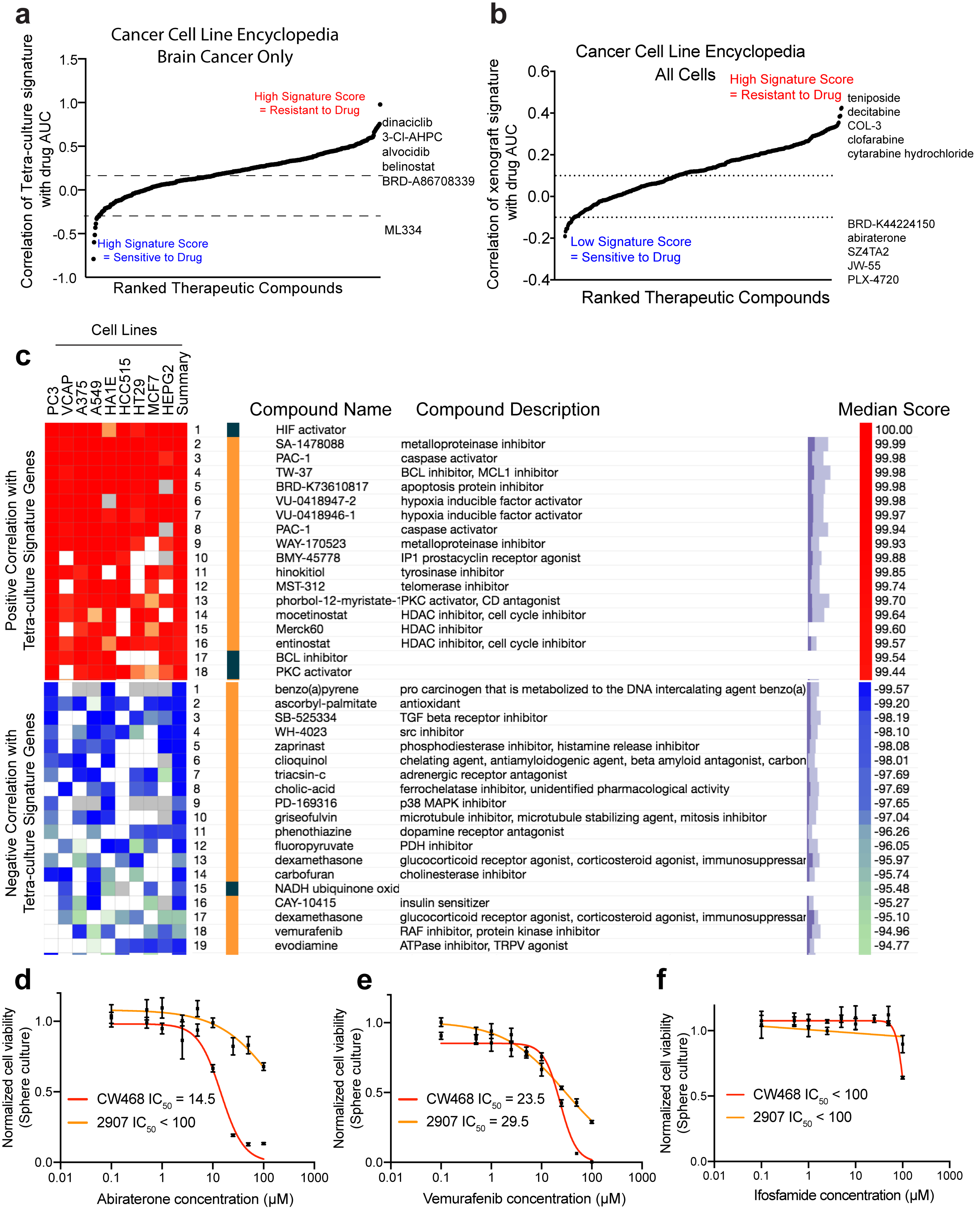
a
Cancer Cell Line Encyclopedia
Brain Cancer Only
Correlation of Tetra-culture signature with drug AUC
1.5
1.0
0.5
0.0
-0.5
-1.0
High Signature Score
= Resistant to Drug
dinaciclib
3-Cl-AHPC
alvocidib
belinostat
BRD-A86708339
ML334
High Signature Score
= Sensitive to Drug
Ranked Therapeutic Compounds
b
Cancer Cell Line Encyclopedia
All Cells
Correlation of xenograft signature with drug AUC
0.6
0.4
0.2
0.0
-0.2
-0.4
High Signature Score
= Resistant to Drug
teniposide
decitabine
COL-3
clofarabine
cytarabine hydrochloride
BRD-K44224150
abiraterone
SZ4TA2
JW-55
PLX-4720
Low Signature Score
= Sensitive to Drug
Ranked Therapeutic Compounds
c
Cell Lines
PC3
VCAP
A375
A549
HA1E
HCC515
HT29
MCF7
HEPG2
Summary
Compound Name
Compound Description
Median Score
Positive Correlation with Tetra-culture Signature Genes
1 HIF activator 100.00
2 SA-1478088 metalloproteinase inhibitor 99.99
3 PAC-1 caspase activator 99.98
4 TW-37 BCL inhibitor, MCL1 inhibitor 99.98
5 BRD-K73610817 apoptosis protein inhibitor 99.98
6 VU-0418947-2 hypoxia inducible factor activator 99.98
7 VU-0418946-1 hypoxia inducible factor activator 99.97
8 PAC-1 caspase activator 99.94
9 WAY-170523 metalloproteinase inhibitor 99.93
10 BMY-45778 IP1 prostacyclin receptor agonist 99.88
11 hinokitiol tyrosinase inhibitor 99.85
12 MST-312 telomerase inhibitor 99.74
13 phorbol-12-myristate-1 PKC activator, CD antagonist 99.70
14 mocetinostat HDAC inhibitor, cell cycle inhibitor 99.64
15 Merck60 HDAC inhibitor 99.60
16 entinostat HDAC inhibitor, cell cycle inhibitor 99.57
17 BCL inhibitor 99.54
18 PKC activator 99.44
Negative Correlation with Tetra-culture Signature Genes
1 benzo(a)pyrene pro carcinogen that is metabolized to the DNA intercalating agent benzo(a) -99.57
2 ascorbyl-palmitate antioxidant -99.20
3 SB-525334 TGF beta receptor inhibitor -98.19
4 WH-4023 src inhibitor -98.10
5 zaprinast phosphodiesterase inhibitor, histamine release inhibitor -98.08
6 clioquinol chelating agent, antiamyloidogenic agent, beta amyloid antagonist, carbon -98.01
7 triacsin-c adrenergic receptor antagonist -97.69
8 cholic-acid ferrochelatase inhibitor, unidentified pharmacological activity -97.69
9 PD-169316 p38 MAPK inhibitor -97.65
10 griseofulvin microtubule inhibitor, microtubule stabilizing agent, mitosis inhibitor -97.04
11 phenothiazine dopamine receptor antagonist -96.26
12 fluoropyruvate PDH inhibitor -96.05
13 dexamethasone glucocorticoid receptor agonist, corticosteroid agonist, immunosuppressar -95.97
14 carbofuran cholinesterase inhibitor -95.74
15 NADH ubiquinone oxid -95.48
16 CAY-10415 insulin sensitizer -95.27
17 dexamethasone glucocorticoid receptor agonist, corticosteroid agonist, immunosuppressar -95.10
18 vemurafenib RAF inhibitor, protein kinase inhibitor -94.96
19 evodiamine ATPase inhibitor, TRPV agonist -94.77
d
Normalized cell viability (Sphere culture)
1.0
0.5
0.0
CW468 IC50 = 14.5
2907 IC50 < 100
0.01 0.1 1 10 100 1000
Abiraterone concentration (μM)
e
Normalized cell viability (Sphere culture)
1.0
0.5
0.0
CW468 IC50 = 23.5
2907 IC50 = 29.5
0.01 0.1 1 10 100 1000
Vemurafenib concentration (μM)
f
Normalized cell viability (Sphere culture)
1.0
0.5
0.0
CW468 IC50 < 100
2907 IC50 < 100
0.01 0.1 1 10 100 1000
Ifosfamide concentration (μM)

Figure S15: Therapeutic efficacy prediction in glioblastoma tetra-culture and xenograft models.

a) Therapeutic efficacy prediction of drugs in brain cancer cells in the CTRP dataset based on differentially expressed genes between the 3D tetra-culture model and GSCs grown in sphere culture as defined by RNA-seq. Therapeutic compounds are ranked based on the correlation between tetra-culture signature expression with drug area under the curve (AUC).

b) Therapeutic efficacy prediction of drugs in all cancer cells in the CTRP dataset based on differentially expressed genes between orthotopic glioblastoma xenografts and GSCs grown in sphere culture as defined by RNA-seq. Therapeutic compounds are ranked based on the correlation between xenograft signature expression with drug area under the curve (AUC).

c) Perturbational database identifies compounds that recapitulate or reverse tetra-culture expression signatures in cancer cell lines. Analysis of the Library of Integrated Network Based Cellular Signatures (LINCS) database (http://www.lincsproject.org) demonstrating the compounds that generate expression signatures that are positively (red) or negatively (blue) correlated with the 3D tetra-culture signature genes.

d) Normalized cell viability of CW468 and 2907 GSCs following treatment with varying doses of abiraterone in sphere culture. IC_{50} values were calculated using a variable slope nonlinear regression model.

e) Normalized cell viability of CW468 and 2907 GSCs following treatment with varying doses of vemurafenib in sphere culture. IC_{50} values were calculated using a variable slope nonlinear regression model.

f) Normalized cell viability of CW468 and 2907 GSCs following treatment with varying doses of ifosfamide in sphere culture. IC_{50} values were calculated using a variable slope nonlinear regression model.

Figure S16

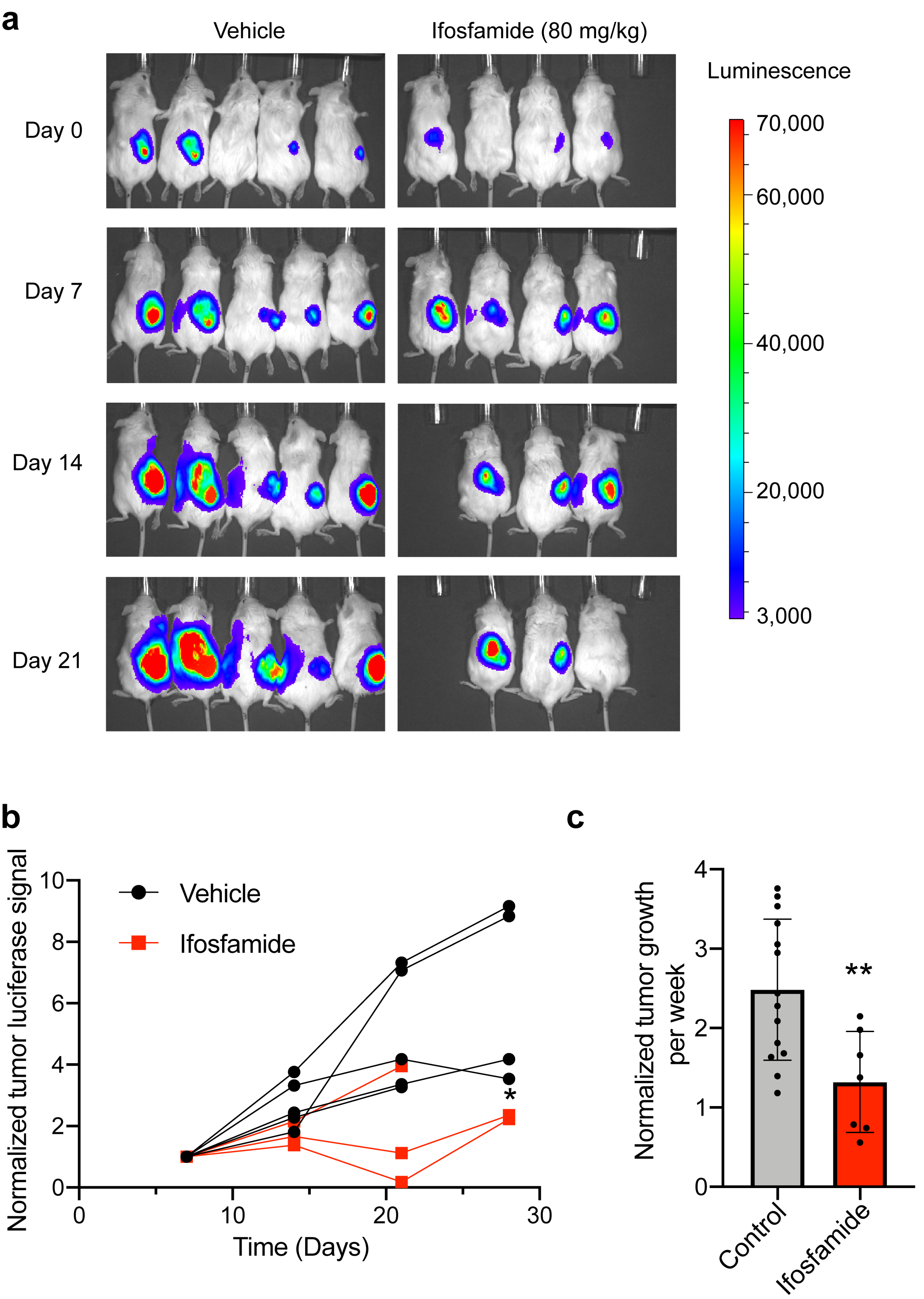
a
Vehicle
Ifosfamide (80 mg/kg)
Day 0
Day 7
Day 14
Day 21
Luminescence
70,000
60,000
40,000
20,000
3,000
b
Normalized tumor luciferase signal
Vehicle
Ifosfamide
Time (Days)
*
c
Normalized tumor growth per week
**
Control
Ifosfamide

Figure S16: Ifosfamide reduces tumor growth in a subcutaneous glioblastoma xenograft model in vivo.

a) Bioluminescence imaging of mice bearing subcutaneous glioblastoma tumors derived from CW468 GSCs over a time course of treatment with ifosfamide (80 mg/kg) or vehicle.

b) Normalized bioluminescent signal for each mouse over a time course of treatment with ifosfamide (80 mg/kg) or vehicle. Tumor growth for each mouse was plotted over time and normalized to the day 7 reading. *, p = 0.02. Two way mixed-effects ANOVA was used for statistical analysis.

c) Normalized tumor growth rate per week plotted for each mouse over a time course of treatment with ifosfamide (80 mg/kg) or vehicle. **, p=0.006. Unpaired two-way t-test was used for statistical analysis.

Figure S17

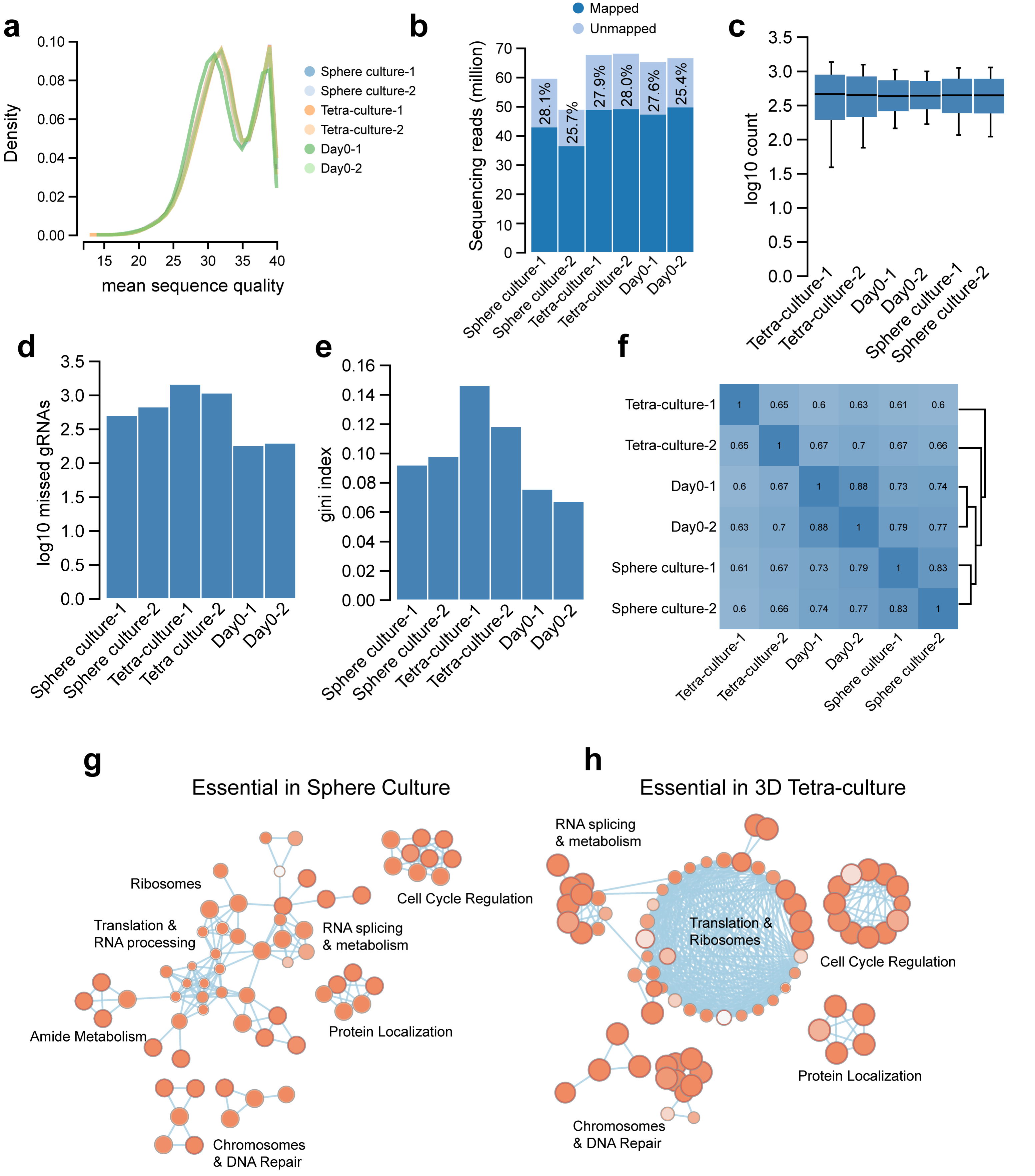
a
Density
mean sequence quality
Sphere culture-1
Sphere culture-2
Tetra-culture-1
Tetra-culture-2
Day0-1
Day0-2
b
Mapped
Unmapped
Sequencing reads (million)
28.1%
25.7%
27.9%
28.0%
27.6%
25.4%
c
log10 count
d
log10 missed gRNAs
e
gini index
f
Tetra-culture-1
Tetra-culture-2
Day0-1
Day0-2
Sphere culture-1
Sphere culture-2
g
Essential in Sphere Culture
Ribosomes
Translation & RNA processing
RNA splicing & metabolism
Cell Cycle Regulation
Protein Localization
Amide Metabolism
Chromosomes & DNA Repair
h
Essential in 3D Tetra-culture
RNA splicing & metabolism
Translation & Ribosomes
Cell Cycle Regulation
Protein Localization
Chromosomes & DNA Repair

Figure S17: Whole genome CRISPR-Cas9 screen reveals context-dependent functional dependencies.

- **a)** Mean sequence quality control metric from whole genome CRISPR-Cas9 loss-of-function screen in 2D culture and the 3D tetra-culture system.
- **b)** Frequency of mapped and unmapped reads from the whole genome CRISPR-Cas9 loss-of-function screen.
- **c)** Read count metrics from the whole genome CRISPR-Cas9 loss-of-function screen.
- **d)** Missing sgRNAs from the whole genome CRISPR-Cas9 loss-of-function screen for each sample.
- **e)** GINI index, measuring the evenness of sgRNA read count across samples from the whole genome CRISPR-Cas9 loss-of-function screen.
- **f)** Pairwise sample correlations (Pearson) for each sample from the whole genome CRISPR-Cas9 loss-of-function screen.
- **g)** Pathway gene set enrichment connectivity diagram displaying pathways enriched among genes essential in sphere cell culture.
- **h)** Pathway gene set enrichment connectivity diagram displaying pathways enriched among genes essential in the 3D tetra-culture model.

Figure S18

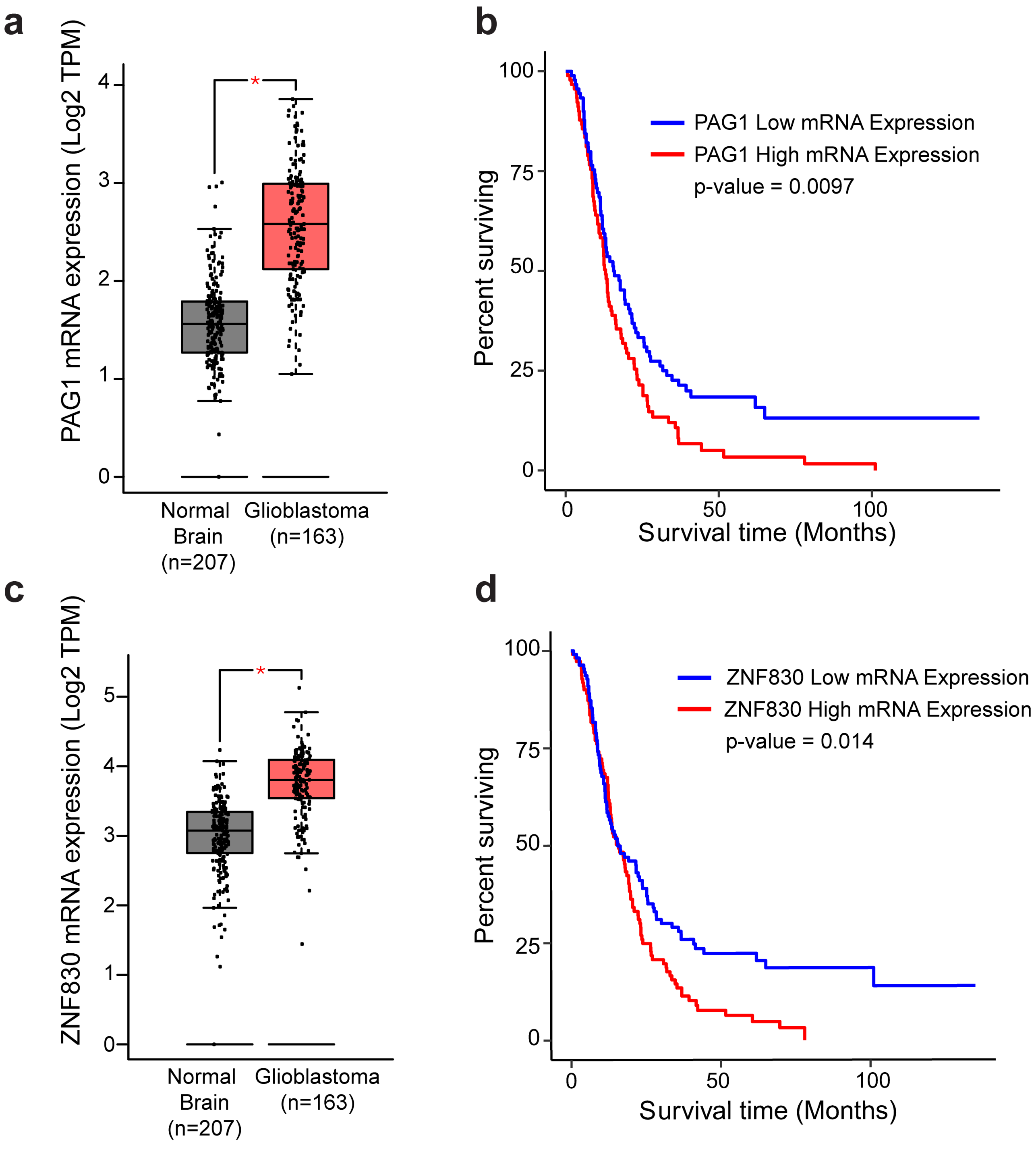

a
PAG1 mRNA expression (Log2 TPM)
0
1
2
3
4
*
Normal Brain (n=207)
Glioblastoma (n=163)
b
Percent surviving
0
25
50
75
100
PAG1 Low mRNA Expression
PAG1 High mRNA Expression
p-value = 0.0097
0
50
100
Survival time (Months)
c
ZNF830 mRNA expression (Log2 TPM)
0
1
2
3
4
5
*
Normal Brain (n=207)
Glioblastoma (n=163)
d
Percent surviving
0
25
50
75
100
ZNF830 Low mRNA Expression
ZNF830 High mRNA Expression
p-value = 0.014
0
50
100
Survival time (Months)

Figure S18: PAG1 and ZNF830 are potential therapeutic targets in glioblastoma.

a) mRNA expression Transcript Per Million (TPM) values in normal brain (GTEx, n=207) and glioblastoma (TCGA, n=163) from RNA-seq data for PAG1. Data were derived from GEPIA (http://gepia.cancer-pku.cn/index.html). Four-way ANOVA controlling for sex, age, and ethnicity with Benjamini and Hochberg false discovery rate (FDR) method was used for statistical analysis. *, $p = 2.61e-33$.

b) Kaplan-Meier survival curve of IDH wild-type primary glioblastoma patients in the Chinese Glioma Genome Atlas dataset based on median mRNA expression of PAG1. PAG1 low expression (n=90), PAG1 high expression (n=90). Log rank test was used for statistical analysis. *, $p = 0.0097$.

c) mRNA expression Transcript Per Million (TPM) values in normal brain (GTEx, n=207) and glioblastoma (TCGA, n=163) from RNA-seq data for ZNF830. Data were derived from GEPIA (http://gepia.cancer-pku.cn/index.html). Four-way ANOVA controlling for sex, age, and ethnicity with Benjamini and Hochberg false discovery rate (FDR) method was used for statistical analysis. *, $p = 2.46e-21$.

d) Kaplan-Meier survival curve of primary glioblastoma patients in the Chinese Glioma Genome Atlas dataset based on median mRNA expression of PAG1. ZNF830 low expression (n=110), ZNF830 high expression (n=110). Log rank test was used for statistical analysis, * $p = 0.014$.

Supplementary Table 1: Immunofluorescence Antibodies

Immunofluorescence Antibody	Species	Dilution	Manufacturer
SOX2	rabbit anti human	1:100	Abcam 97959
GFAP	mouse anti human	1:100	MilliporeSigma G3893
CA9	rabbit anti human	1:100	Novus Biologicals NB100-417
Ki67	rabbit anti human	1:100	Abcam ab16667
MAP2	mouse anti human	1:100	R&D systems MAB304
TUBB3 (TUJ1)	mouse anti human	1:100	R&D systems MAB1195
OLIG2	mouse anti-human	1:100	Millipore MABN50
EGFR (D38B1)	rabbit anti-human	1:50	Cell Signaling Technologies #4267
PhosphoEGFR Y1173	mouse anti-human	1:200	MilliporeSigma 16244

Supplementary Table 2: qPCR Primers

Gene	Accession Number	Forward Primer (5'->3')	Reverse Primer (5'->3')
**GAPDH**	NM_002046.7	ACAACTTTGGTATCGTGGAAGG	GCCATCACGCCACAGTTTC
**SOX2**	NM_003106.4	TACAGCATGTCCTACTCGCAG	GAGGAAGAGGTAACCACAGGG
**OLIG2**	NM_005806.4	TGGCTTCAAGTCATCCTCGTC	ATGGCGATGTTGAGGTCGTG
**MAP2**	XM_017004138.2	CTCAGCACCGCTAACAGAGG	CATTGGCGCTTCGGACAAG
**TUJ1**	NM_006086.4	GGCCAAGGGTCACTACACG	GCAGTCGCAGTTTTCACACTC
**CD163**	NM_203416.3	AAAAAGCCACAACAGGTCGC	CTTGAGGAAACTGCAAGCCG
**IL10**	NM_000572.3	TACGGCGCTGTCATCGATTT	TAGAGTCGCCACCCTGATGT
**IL4Ra**	NM_001257406.1	GACCTGGAGCAACCCGTATC	AATCTGCCGGGTCGTTTTCA
**TNFa**	NM_000594.4	AGAACTCACTGGGGCCTACA	GCTCCGTGTCTCAAGGAAGT
**NOS2**	NM_000625.4	CGCATGACCTTGGTGTTTGG	CATAGACCTTGGGCTTGCCA
**CA9**	NM_001216	GGATCTACCTACTGTTGAGGCT	CATAGCGCCAATGACTCTGGT
**NDRG1**	NM_006096	CTCCTGCAAGAGTTTGATGTCC	TCATGCCGATGTCATGGTAGG
**COL6A2**	NM_058174	TACGGAGAGTGCTACAAGGTG	GGTCCTGGGAATCCAATGGG
**COL1A1**	NM_000088	GAGGGCCAAGACGAAGACATC	CAGATCACGTCATCGCACAAC
**LOX**	NM_001178102	CGGCGGAGGAAAACTGTCT	TCGGCTGGGTAAGAAATCTGA
**RUNX2**	NM_001015051	TGGTTACTGTCATGGCGGGTA	TCTCAGATCGTTGAACCTTGCTA
**STAT6**	NM_001178080	GTTCCGCCACTTGCCAATG	TGGATCTCCCCTACTCGGTG
**TLR4**	NM_138557	AGACCTGTCCCTGAACCCTAT	CGATGGACTTCTAAACCAGCCA
**HIF3A**	NM_152795	ATGCGGTCAGCAAGAGCATC	AGACGATACTCTCCGACTGGG

Supplementary Table 3: CRISPR-Cas9 sgRNA Oligonucleotide Sequences

Gene Targeted	Guide Designation	Short Name	Forward sgRNA Sequence	Reverse sgRNA Sequence
**Non-targeting Control**	sgCONT	sgCONT	CACCGCTCTGCTGCGGAAGGATTCG	AAACCGAATCCTTCCGCAGCAGAGC
**PAG1**	PAG1_43495	sgPAG1.1	CACCGAACTGTGAAAGAGATCAAGG	AAACCCTTGATCTCTTTCACAGTTC
**PAG1**	PAG1_43498	sgPAG1.9	CACCGTGAGTTTGCTGAATATGCCT	AAACAGGCATATTCAGCAAACTCAC
**ZNF830**	ZNF830_55900	sgZNF830.3	CACCGTAAATCAGGAAGAATTGCGG	AAACCCGCAATTCTTCCTGATTTAC
**ZNF830**	ZNF830_55899	sgZNF830.1	CACCGGGAAAGGAGAGAAAACACCG	AAACCGGTGTTTTCTCTCCTTTCCC
**RNF19A**	RNF19A_34020	sgRNF19A.20	CACCGAATATCAAGCGAATATCATG	AAACCATGATATTCGCTTGATATTC
**RNF19A**	RNF19A_34019	sgRNF19A.19	CACCGAAGACACAACCCAAGCATAG	AAACCTATGCTTGGGTTGTGTCTTC
**ATP5H**	ATP5H_28215	sgATP5H.15	CACCGTCAGAAACATACTGACCTGG	AAACCCAGGTCAGTATGTTTCTGAC
**ATP5H**	ATP5H_28213	sgATP5H.13	CACCGGAATCCACCAGCTATCGACT	AAACAGTCGATAGCTGGTGGATTCC

Supplementary Table 4: Western Blot Antibodies

Western Blot Antibody	Species	Dilution	Manufacturer
**PAG1**	Rabbit anti human	1:1000	Abcam ab14989
**ZNF830**	Rabbit anti human	1:2000	Novus Biologicals NB100-68229
**ATP5H**	Rabbit anti human	1:1000	Proteintech 17589-1-AP
**RNF19A**	Rabbit anti human	1:1000	Bethyl Laboratories A303-105A
**FLAG**	Mouse anti human	1:2000	Sigma F1804
**Tubulin**	Mouse anti-human	1:2000	Sigma T6074
**EGFR (D38B1)**	Rabbit anti human	1:1000	Cell Signaling Technologies #4267
**Phospho-EGFR Y1173 (53A5)**	Rabbit anti human	1:1000	Cell Signaling Technologies 4407
**Phospho-EGFR Y1068 (D7A5)**	Rabbit anti human	1:1000	Cell Signaling Technologies #3777
